# Comparative Analysis of Individual Carotenoid Profiles in Yellow- and White-Fleshed Potatoes (*Solanum tuberosum* L.) During Tuber Development

**DOI:** 10.3390/foods13223691

**Published:** 2024-11-20

**Authors:** Haicui Suo, Jitao Liu, Li Wang, Chengchen Li, Jianwei Shan, Kang An, Kun Yang, Xiaobo Li

**Affiliations:** Crops Research Institute, Guangdong Academy of Agricultural Sciences, Guangdong Provincial Key Laboratory of Crop Genetic Improvement, Guangzhou 510640, China

**Keywords:** potato, individual carotenoid, carotenoid esters, accumulation patterns

## Abstract

Individual carotenoids provide significant health benefits to humans, and potatoes are recognized as some of the most stable crops, distinguished by their substantial carotenoid content. To elucidate the accumulation patterns of individual carotenoids in potatoes, we quantified the carotenoid content in yellow- and white-fleshed genotypes across five developmental stages using LC-MS/MS. A total of 22 carotenoids were identified in yellow potatoes, whereas 18 were detected in white potatoes. The yellow-fleshed genotype was characterized by high levels of individual carotenoids and xanthophyll esters, with violaxanthin as the dominant component. The white-fleshed genotype exhibited low concentrations of individual carotenoids and xanthophyll esters, with lutein as the predominant compound. Notably, lutein, violaxanthin, zeaxanthin, antheraxanthin, neoxanthin, violaxanthin myristate, and lutein myristate were consistently detected during the developmental period in both genotypes. Violaxanthin myristate was identified as the dominant xanthophyll ester in both genotypes, showing an increasing trend throughout the tuber development stages. In contrast, xanthophyll esters maintained stable low levels in white-fleshed potatoes while exhibiting increasing types and contents in yellow-fleshed potatoes as the tubers continued to grow. Additionally, violaxanthin exhibited a significant correlation with b*, H°, and C*, suggesting that it may play an important role in forming yellow flesh.

## 1. Introduction

The contemporary lifestyle of society has evolved towards rapid mobilization and advanced technology, leading to poor dietary patterns characterized by excessive intake of macronutrients while neglecting micronutrients. This imbalance results in micronutrient deficiencies, particularly among children and pregnant women [1]. Carotenoids, which are required in trace amounts, have garnered attention for their beneficial contributions to human health, including enhanced immune function and a reduced risk of developing degenerative chronic diseases such as age-related macular degeneration, type 2 diabetes, obesity, certain cancers, and cardiovascular diseases [2,3,4,5,6]. Naturally occurring carotenoids predominantly consist of C40 hydrocarbons that may contain oxygenated functional groups; these compounds are essential for photoprotection and serve as precursors to phytohormones. As secondary metabolites, carotenoids play significant roles in the communication between plants and animals. They act as natural colorants and aroma precursors that attract pollinators and seed-dispersing animals.

Carotenoids are abundant in fruits, vegetables, and cereal grains. Up to 2018, approximately 850 types of carotenoids have been identified. Potatoes, one of the most important staple crops globally, are recognized as a primary source of micronutrients like carotenoids due to their widespread consumption [7]. The carotenoid composition within potato tubers is influenced by genetic factors as well as environmental conditions and geographical location [8]. Various species of potato tubers are cultivated worldwide, including *Stenotomum*, *Andigena*, and *Phureja*; among them is the group *Tuberosum*, which is the most widely distributed [9]. Notably, high levels of total carotenoids have been identified in the flesh of potatoes from the *Phureja* group [10,11]. Xanthophyll, like lutein, violaxanthin, zeaxanthin, and antheraxanthin, was the dominant carotenoid, while carotene was in lower levels [11,12,13]. Furthermore, earlier studies also revealed that yellow-fleshed potato genotypes typically exhibit higher levels of carotenoids, with violaxanthin being predominant, while white- or cream-fleshed potato genotypes show lower levels of carotenoids, with lutein predominating [7,12,14].

Esterification with fatty acids provides stable storage by enhancing the efficiency of xanthophyll packaging into plastoglobules while likely protecting xanthophylls from catabolism through steric hindrance against enzymes responsible for catalyzing carotenoid cleavage, a hypothesis yet to be fully substantiated [15]. The first report on carotenoid esters derived from *S. tuberosum* was published in 1980 [16]. It noted that yellow coloration arises from xanthophyll accumulation, which directly correlates with esterification processes [16]. Individual xanthophyll esters were identified specifically within yellow-fleshed varieties in recent studies [7,12]. Additionally, studies on rapeseed flowers (*Brassica juncea* L.) suggest an integrated model governing yellow petal coloration regulated by xanthophyll esterification and plastoglobule formation [17]. However, mechanisms underlying esterification across other vital plant tissues, including fruits (e.g., bananas, oranges, papayas, and peppers) and tubers (e.g., potatoes), remain largely unexplored [15]. The color quality trait exhibited by edible flesh significantly influences consumer preferences regarding potatoes. To date, only a limited number of researchers have documented the changes in total carotenoid content and main individual carotenoids during tuber development [8,13,18]. Furthermore, comprehensive data detailing accumulation patterns of individual carotenoids throughout maturation stages remain scarce, especially for more commercial varieties of *S. tuberosum* with yellow or white flesh. Consequently, a detailed comparative analysis was conducted on individual carotenoid profiles across two *S. tuberosum* potato genotypes with yellow and white flesh, respectively, at five developmental stages. The accumulation patterns of carotenoids, including carotene, xanthophyll, and xanthophyll esters, are illustrated here, and the coloration of potato flesh is also discussed.

## 2. Materials and Methods

### 2.1. Plant Materials

Two potato genotypes, LY1835-4 and LY1835-2, with yellow and white flesh colors, respectively, were used for carotenoid analysis and were planted using the standard production practices at the Baiyun experimental station (23°23′ N, 113°26′ E, 20 m above sea level) at the Guangdong Academy of Agricultural Sciences, Guangzhou, China. Samples were taken in triplicate at the developmental stages of tuber weight in the range of 0.5–1.5 g, 5–10 g, 50–70 g, 100–150 g, and 200–220 g. After rapid peeling, the potatoes were cut into 0.5 × 0.5 pieces, frozen in liquid nitrogen for 2 min, and then transferred to a refrigerator at −80 °C for storage.

### 2.2. Color Measurement

The samples for color measurement were ground to powder and then lyophilized. The lyophilized powder was scanned using an A3 Unis Scanner (Uniscan^®^ M1 Plus, Unisplendour Corporation Limited, Beijing, China), and the International Commission on Illumination CIE 1976 (L*, a*, b*) color space values were measured using the Tomato Analyzer 3.0 software [19]. Before measuring the color, the software was standardized with a color calibration card. The average of the three corresponding readings of each genotype was considered and analyzed statistically as the final values. The results were described as L* (brightness or lightness, positive towards white and negative towards black), a* (red–green, positive towards red and negative towards green), b* (yellow–blue, positive towards yellow and negative towards blue), H° (hue angle, calculated from the arctangent of b*/a*, 0° or 360° towards red, 90° towards yellow, 180° towards green, and 270° towards blue), and C* (chroma, calculated as $\sqrt{a^{*2}+b^{*2}}$) [20].

### 2.3. Determination of Carotenoids

#### 2.3.1. Chemicals and Reagents

HPLC-grade methanol (MeOH), ethanol (EtOH), and acetonitrile (ACN) were obtained from Merck, located in Darmstadt, Germany. Butylated hydroxytoluene (BHT) was sourced from Aladdin, while acetone was supplied by Sinopharm. Methyl tert-butyl ether (MTBE) came from Anpel (CNW), sodium chloride (NaCl) was acquired from Rhawn, and potassium hydroxide (KOH) was provided by Hushi. For all experimental procedures, MilliQ water supplied by Millipore in Bradford, USA, was used. All standards were purchased from Sigma-Aldrich, based in St. Louis, MO, USA, and BOC Group Inc. (BOC), situated in New York, NY, USA. Additionally, formic acid was also obtained from Sigma-Aldrich. Stock solutions of the standards were created at a concentration of 1 mg/mL using MTBE/MeOH as the solvent and stored at −20 °C for preservation purposes.

#### 2.3.2. Sample Preparation and Extraction

The sample was subjected to freeze-drying and then ground into a powder using a frequency of 30 Hz for 1.5 min, after which it was stored at −80 °C until required. A quantity of 50 mg of the powder was measured and extracted with 0.5 mL of a mixed solution consisting of n-hexane, acetone, and ethanol in a ratio of 1:1:1 (*v*/*v*/*v*). The mixture was vortexed for 20 min at room temperature. Following this, the supernatants were collected after centrifugation at 12,000 rpm for 5 min at 4 °C. The remaining residue underwent re-extraction by repeating the aforementioned steps under identical conditions. Subsequently, the extract was evaporated to dryness and reconstituted in a mixed solution of MeOH/MTBE (1:1, *v*/*v*). Finally, the resulting solution was filtered through a membrane filter with a pore size of 0.22 μm prior to liquid chromatography–tandem mass spectrometry (LC-MS/MS) analysis.

#### 2.3.3. Ultra-Performance Liquid Chromatography (UPLC) Conditions

The sample extracts were analyzed using an ultra-performance liquid chromatography–atmospheric pressure chemical ionization tandem mass spectrometry (UPLC-APCI-MS/MS) system, specifically the ExionLC™ AD (SCIEX, Foster City, CA, USA) for UPLC and the Applied Biosystems 6500 Triple Quadrupole mass spectrometer (SCIEX, Foster City, CA, USA). The analytical parameters were as follows: the LC column used was a Yantai Zhenghai Electronic Mask Corporation Limited (YMC) C30 (Yantai, China) (3 μm, 100 mm × 2.0 mm i.d.); the solvent system comprised methanol and acetonitrile in a ratio of 1:3 (*v*/*v*), supplemented with 0.01% BHT and 0.1% formic acid (designated as A), while methyl tert-butyl ether containing 0.01% BHT served as solvent B; the gradient program commenced at 0% B for the first three minutes, increased to 70% B from minutes three to five, then escalated to 95% B between five and nine minutes, before returning to 0% B from ten to eleven minutes; flow rate was maintained at 0.8 mL/min; temperature was set at 28 °C; injection volume was fixed at 2 μL [21].

#### 2.3.4. APCI-Q Trap-MS/MS

MS/MS detection was carried out using the AB 6500 Triple Quadrupole LC-MS/MS System (SCIEX, Foster City, CA, USA), which features an atmospheric pressure chemical ionization (APCI) heated nebulizer interface. The operational parameters for the APCI source included an ion source set to APCI+, a source temperature of 350 °C, and curtain gas (CUR) maintained at 25.0 psi. Declustering potential (DP) and collision energy (CE) for each multiple reaction monitoring (MRM) transition were optimized further as needed. A designated set of MRM transitions was monitored during specific time periods based on the carotenoids that eluted at those times. Carotenoid analysis utilized the Metware database (MWDB, Wuhan, China), developed from standards to qualitatively assess mass spectrometry data. LC-MS/MS data pertaining to carotenoids were analyzed with Analyst 1.6.3 software from AB Sciex (SCIEX, Foster City, CA, USA) using default settings for automatic identification of changes and integration in the MRM process. Retention times and ion pair information for carotenoids were employed to adjust chromatographic peaks across different samples (Figure S1). Standard curves for various carotenoids were created by plotting the concentration ratio of the external standard against the internal standard on the *x*-axis versus peak area ratios on the *y*-axis. Subsequently, the integral peak area ratio of each detected carotenoid in the samples was substituted into these standard curves to determine concentrations accurately. Finally, absolute carotenoid content in actual samples was calculated using this formula: carotenoid content (mg/g) = c*V/1000/m, where c represents the concentration derived from substituting integrated peak area ratios into standard curves (mg/mL), V is the resuspension volume (mL), and m denotes the sample weight (g).

### 2.4. Data Processing

All data are presented as the averages of three replicates. Duncan’s test was conducted at a significance level of 0.05 using SPSS v19.0 software (SPSS Inc., Chicago, IL, USA). Pearson’s correlation coefficients were calculated to assess relationships between variables. Principal component analysis (PCA) was carried out using R software, R vision 4.4.1 (14 June 2024).

## 3. Results

### 3.1. Color Assessment

**Figure 1:**
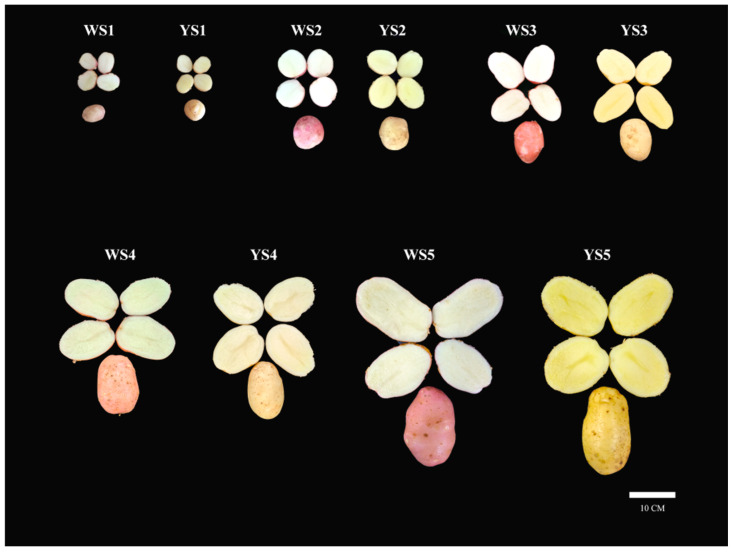
The flesh color of two genotypes at five development stages. WS1, WS2, WS3, WS4, and WS5 represent the white-fleshed potato genotype LY1835-2 at the development stage of a fresh weight of 0.5–1.5 g, 5–10 g, 50–70 g, 100–150 g, and 200–220 g, respectively. YS1, YS2, YS3, YS4, and YS5 represent the yellow-fleshed potato genotype LY1835-4 at the development stage of a fresh weight of 0.5–1.5 g, 5–10 g, 50–70 g, 100–150 g, and 200–220 g, respectively.

Two potato genotypes with yellow and white flesh at five developmental stages named YS1 to YS5 and WS1 to WS5, respectively, were used in this study (Figure 1). To understand the color difference between these two genotypes, the color parameters (L*, a*, b*, H°, and C*) of flesh were measured and illustrated (Table 1). The parameter values exhibited significant variation between the two potato genotypes. Notably, the parameters b*, C*, and H° demonstrated a consistent tendency across the genotypes; the values of b*, C*, and H° were significantly higher in yellow-flesh potatoes compared to white-flesh ones, with YS2 exhibiting the highest levels, followed by other developmental stages of yellow flesh. Conversely, WS5 displayed the lowest levels. Interestingly, the parameters L* and a* showed an opposing trend; the values of L* and a* were higher in white-fleshed potatoes than in yellow-fleshed ones, with WS5 presenting the highest levels.

### 3.2. Carotenoids Composition

In the present study, 25 carotenoids were identified in the nonsaponified extracts from two potato genotypes across five developmental stages (Table 2). Among these, 10 were classified as free carotenoids or intermediates involved in the carotenoid biosynthetic pathway, comprising 3 carotenes and 7 xanthophylls, while an additional 15 xanthophylls were found in esterified forms.

**Figure 2:**
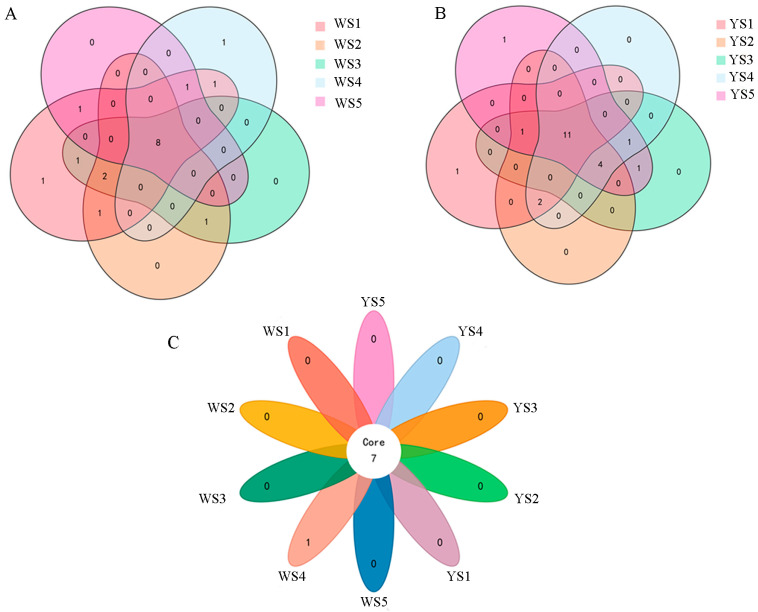
Venn diagram analysis of the carotenoids in two genotypes at five developmental stages. (**A**) Venn diagram of the carotenoids in the white-fleshed genotype at five developmental stages. (**B**) Venn diagram of the carotenoids in the yellow-fleshed genotype at five developmental stages. (**C**) Venn diagram of the carotenoids in two genotypes at five developmental stages. The numbers in nonoverlapping regions represent the quantities of carotenoids detected specific at a certain developmental stage, and the numbers in graphically overlapping regions represent the quantites of carotenoids detected shared at two or more developmental stages.

In the white-fleshed genotype, 18 carotenoids were detected, including 3 carotenes, 7 xanthophylls, and 8 xanthophyll esters, with 8 carotenoids characterized in five developmental stages (Figure 2A, Table S1). Conversely, in the yellow-fleshed genotype, 22 carotenoids were identified, consisting of 2 carotenes, 7 xanthophylls, and 13 xanthophyll esters, with 11 carotenoids characterized in five developmental stages (Figure 2B, Table S1). Notably, lutein, violaxanthin, zeaxanthin, antheraxanthin, neoxanthin, and both violaxanthin myristate and lutein myristate were consistently detected at all five developmental stages in both genotypes; however, α-carotene was exclusively identified in the white-fleshed genotype (Figure 2C, Table S1). This suggests minimal differences in the composition of carotenes and free lutein between white- and yellow-fleshed genotypes. In contrast to this finding regarding free compounds, six specific xanthophyll esters (lutein dimyristate, lutein myristate, lutein palmitate, violaxanthin myristate, violaxanthin-myristate-laurate, and violaxanthin laurate) were observed in both genotypes. Additionally, seven xanthophyll esters (lutein dilaurate, violaxanthin dimyristate, violaxanthin palmitate, violaxanthin-myristate-palmitate, violaxanthin dipalmitate, violaxanthin dilaurate, and zeaxanthin dimyristate) were identified solely within yellow-fleshed potatoes, whereas two esters (violaxanthin dibutyrate and zeaxanthin dipalmitate) were characterized only within white-fleshed potatoes, which indicated significant disparities concerning esterification intensity between these two genotypes (Table S1).

**Figure 3:**
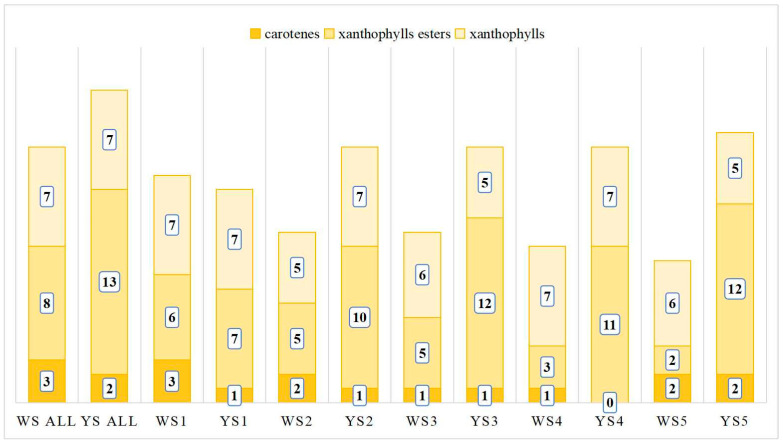
Carotenoid composition of the development of tubers from yellow- and white-fleshed genotypes measured by LC-MS/MS. The numbers in the light yellow color represent the quantities of carotenes detected, the numbers in the middle yellow color represent the quantities of xanthophy esters detected, and the numbers in the dark yellow color represent the quantites of xanthophy detected.

**Figure 4:**
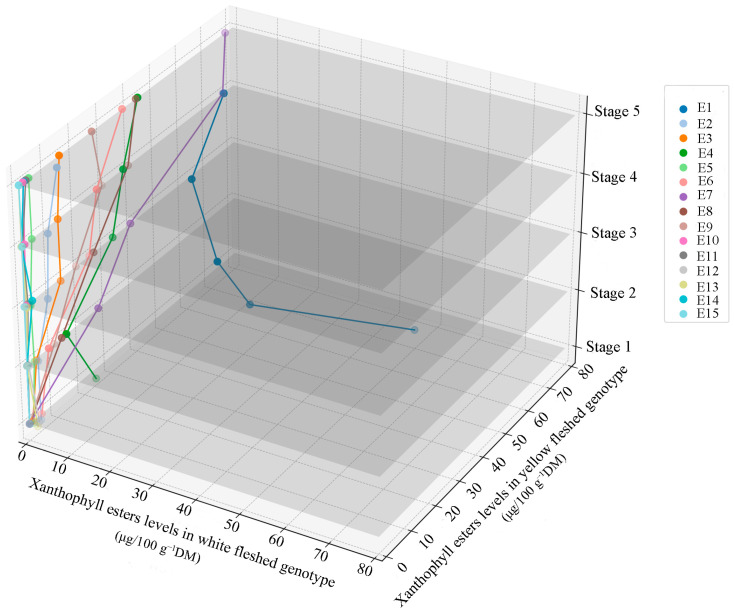
The accumulation pattern of xanthophyll esters during the tuber development. E1 to E15 represent violaxanthin myristate, lutein myristate, lutein dimyristate, violaxanthin-myristate-laurate, lutein palmitate, violaxanthin dimyristate, violaxanthin palmitate, violaxanthin-myristate-palmitate, violaxanthin dipalmitate, zeaxanthin dimyristate, lutein dilaurate, violaxanthin laurate, violaxanthin dibutyrate, violaxanthin dilaurate, and zeaxanthin dipalmitate, respectively.

The carotenoids identified across the five developmental stages of these two genotypes exhibited significant variation. Specifically, 16, 12, 12, 11, and 10 carotenoids were detected in the white-fleshed genotype, while the yellow-fleshed genotype revealed counts of 15, 18, 18, 18, and 19 from the first to fifth developmental stages, respectively. Notably, there was minimal difference between these two genotypes at the initial developmental stage. However, during the second through fifth stages of development, the quantity of carotenoids observed in the yellow-fleshed genotype was significantly greater than that found in its white-fleshed counterpart. This disparity was particularly pronounced for xanthophyll esters; specifically, from the second to fifth developmental stages, there were 5, 6, 9, and 10 more xanthophyll esters present in the yellow-fleshed genotype compared to the white-fleshed genotype (Figure 3 and Figure 4).

### 3.3. Individual Carotenoid Contents

In the white-fleshed genotype, lutein made up the predominant part of the carotenoid profile at every stage of the tuber development; its abundance reached the peak at the first developmental stage and the minimum value at the fourth stage, with an average value range of 148.79–371.93 μg/100 g^−1^DM. The second dominant carotenoid in the white-fleshed genotype was violaxanthin, with an average value range from 39.12 to 197.26 μg/100 g^−1^DM, and its levels reached the maximum in early tuber development, later continually decreasing as tuber development progressed to mature. Antheraxanthin and neoxanthin, with average levels of 7.33–17.02 μg/100 g^−1^DM and 5.6–41.35 μg/100 g^−1^DM, were ranked third and fourth, respectively. Zeaxanthin, with an average value of 3.34–6.537.33–17.02 μg/100 g^−1^DM, is the fifth major carotenoid. In contrast, in the yellow-fleshed genotype, violaxanthin was the major carotenoid, with an average value range of 399.08–679.97 μg/100 g^−1^DM, and the levels were highest in the first development stage and decreased in subsequent development stages. This is followed by lutein, with an average value ranging from 286.75 to 430.06 μg/100 g^−1^DM; it peaked in the first developmental stage, then went down and rose again, but did not return to the maximum level. Also, antheraxanthin and neoxanthin were the third and fourth carotenoids, with average levels ranging from 29.33 to 94.95 μg/100 g^−1^DM and 32.96 to 87.63 μg/100 g^−1^DM, respectively. Zeaxanthin is still in fifth place with an average value of 4.32–10.99 μg/100 g^−1^DM. In both varieties, canthaxanthin and echinenone were the minor constituents and were not detected in many stages. Notably, throughout the tuber developmental process, the levels of lutein, violaxanthin, antheraxanthin, zeaxanthin, and neoxanthin in the yellow-fleshed genotype were significantly higher than those in the white-fleshed genotype (Table 3).

Among xanthophyll esters, violaxanthin myristate was the most abundant in both genotypes, ranging from 14.13 to 49.42 μg/100 g^−1^DM in the white-fleshed and 34.32 to 70.80 μg/100 g^−1^DM in the yellow-fleshed genotypes, showing an increasing trend with the tuber development stage. What is more, the average value of violaxanthin myristate was significantly higher in yellow-fleshed than white-fleshed potatoes at each developmental stage. In white-fleshed potatoes, lutein myristate played a second dominant role, with an average content of 0.89–2.38 μg/100 g^−1^DM. In addition to these two types of xanthophyll esters, others were present in trace amounts or undetectable. In contrast, in the yellow-fleshed genotype, the levels of violaxanthin-myristate-laurate and violaxanthin dimyristate were next only to the violaxanthin myristate, ranging from 14.21 to 42.79 μ g 100g^−1^DM and 4.41 to 36.99 μg 100g^−1^DM, respectively, and their levels increased with the development stage. In addition, in yellow-fleshed tubers, lutein myristate, lutein dimyristate, and lutein palmitate were also detected in the five developmental stages with average levels of 2.41–9.7 μg/100 g^−1^DM, 1.19–14.1 μg/100 g^−1^DM, and 0.63–3.41 μg/100 g^−1^DM, respectively. Furthermore, violaxanthin palmitate, violaxanthin myristate palmitate, and violaxanthin dipalmitate were undetectable in the first developmental stage and the levels increased from the second to fifth stages, with contents ranging from 26 to 77.03 μg/100 g^−1^DM, 12.48 to 42.10 μg/100 g^−1^DM, and 9.83 to 25.80 μg/100 g^−1^DM, respectively, in the yellow-fleshed genotype. Other xanthophyll esters, such as zeaxanthin dimyristate, lutein dilaurate, violaxanthin laurate, and violaxanthin dibutyrate, appeared in minor amounts and were detected only at specific development stages in the yellow-flesh genotype (Table 3, Figure 4).

**Figure 5:**
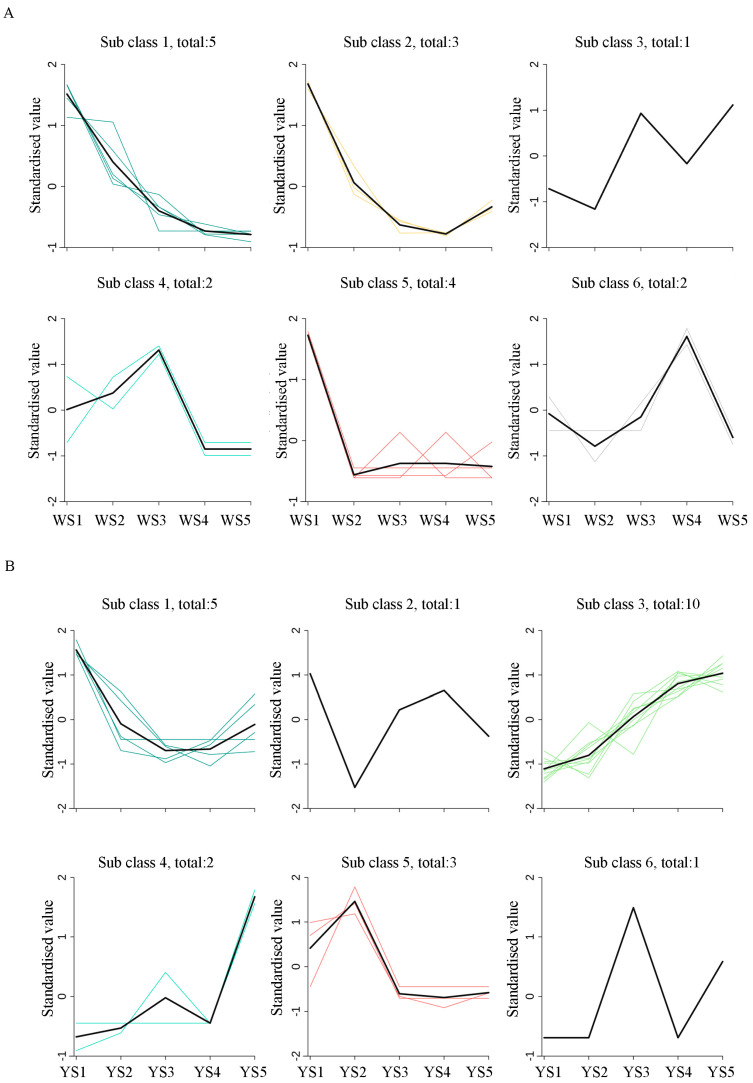
K-means cluster analysis of carotenoids in white flesh potatoes (**A**) and yellow flesh potatoes (**B**). Sub-class represents the carotenoid category number with the same change trend, and the total number represents the number of carotenoids in this category. Each colored line illustrates the trend in the content of a specific carotenoid throughout the developmental period, while the black line illustrates the overall trend in carotenoid content in each category throughout the developmental period.

The analysis of the change trends in carotenoid content revealed that the patterns varied between the two potato genotypes with the exception of lutein, which had the same trend in both genotypes, showing a sharp decline from the second stage and a slight rise in the mature stage. Notably, in the white-fleshed genotype, five carotenoids, including α-carotene, violaxanthin, neoxanthin, violaxanthin myristate, and violaxanthin laurate, demonstrated a declining trend as classified in subclass 1. Similarly, four others, including γ-carotene and echinenone, followed a downward trajectory within subclass 5. Meanwhile, three substances (γ-carotene, lutein, and zeaxanthin) initially decreased but then showed slight rebounds as categorized in subclass 2 (Figure 5A). In contrast, in the yellow-fleshed genotype, most carotenoids exhibited a consistent upward trend as tubers developed; this included antheraxanthin and nine xanthophyll esters categorized under subclass 3. Conversely, five carotenoids, α-carotene, lutein, neoxanthin, violaxanthin myristate, and violaxanthin laurate, initially decreased before experiencing a slight increase and were classified under subclass 1. Additionally, three carotenoids, violaxanthin, canthaxanthin, and echinenone, peaked during the second stage before undergoing continuous decline (Figure 5B).

### 3.4. Detection of Fatty Acids

We conducted a comprehensive analysis of the fatty acid content profiles in two potato tubers across various developmental stages, revealing slight differences in both the types and concentrations of fatty acids between the two genotypes, with palmitic acid identified as the predominant component. As the potato tubers developed, the concentration of palmitic acid decreased from 270.14 to 151.16 μg/g in the white-fleshed genotype and 238.58 to 151.98 μg/g in the yellow-fleshed one. Following this trend, stearic acid also exhibited a decline; its concentration decreased from 130.82 to 92.12 μg/g in the white-fleshed genotype and from 105.2 to 92.76 μg/g in the yellow-fleshed genotype as the tuber development progressed. Additionally, other fatty acids were detected at lower levels throughout the development phases. While palmitic acid and myristic acid served as the primary donor fatty acids for esterification processes, myristic acid remained present at relatively low concentrations during all stages of development (Table 4).

### 3.5. Correlation Analysis

**Figure 6:**
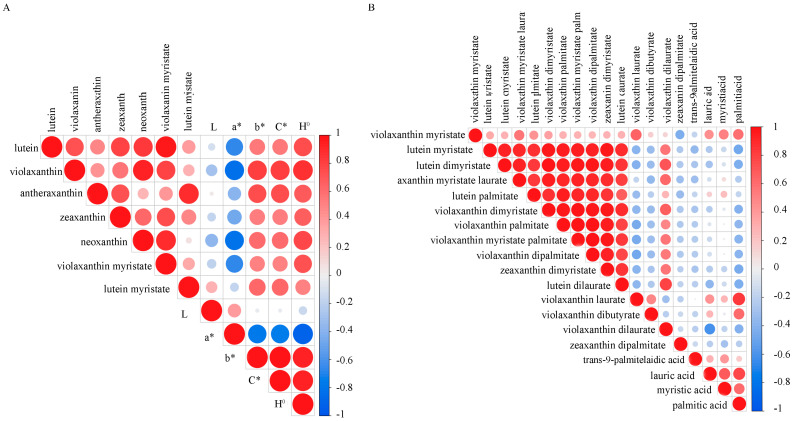
Correlation analysis of the main carotenoids and the potato color parameters (**A**) and the main fatty acids and their xanthophyll esters (**B**).

Seven carotenoids (lutein, violaxanthin, zeaxanthin, antheraxanthin, neoxanthin, and both violaxanthin myristate and lutein myristate) identified in both genotypes across the five developmental stages were selected to conduct a correlation analysis with potato color, and the carotenoid content showed a great correlation with the color (L*, a*, b*, H°, and C*). There was no correlation between carotenoids and L* value, but a negative correlation between these seven carotenoids and a* value, particularly for violaxanthin and neoxanthin, which demonstrated highly significant negative correlations with a*. The color parameters b*, H°, and C* generally reflected the same correlation trends with several carotenoids. Notably, violaxanthin showed a strong, significant positive correlation with b*, H°, and C*. Furthermore, lutein, zeaxanthin, antheraxanthin, violaxanthin myristate, and lutein myristate exhibited certain correlations with b*, H°, and C* values, respectively (Figure 6A).

To verify the relationship between fatty acids and esterification, a correlation analysis between the main donor fatty acids (trans-9-palmitelaidic acid, lauric acid, myristic acid, and palmitic acid) and the xanthophyll esters was performed. It showed that the lutein myristate, lutein dimyristate, violaxanthin-myristate-laurate, lutein palmitate, violaxanthin dimyristate, violaxanthin palmitate, violaxanthin-myristate-palmitate, violaxanthin dipalmitate, zeaxanthin dimyristate, and lutein dilaurate exhibited a highly significant correlation with each other. However, none of the xanthophyll esters showed any correlation with trans-9-palmitelaidic acid. Palmitic acid was the most abundant fatty acid in both genotypes; violaxanthin laurate showed a very significant positive correlation with palmitic acid, while violaxanthin dibutyrate and violaxanthin myristate were moderately correlated with palmitic acid. Additionally concerning lauric and myristic acids, there were also moderate correlations observed for these two fatty acids alongside violaxanthin myristate (Figure 6B).

## 4. Discussion

In the present study, 25 carotenoids were identified in the nonsaponified extracts from potato tubers at five developmental stages. Among these carotenoids, β-carotene, zeaxanthin, violaxanthin, neoxanthin, lutein, and antheraxanthin were identified in earlier studies by HPLC-DAD [11,13,14,22,23,24] and LC-MS [10,12,25,26]. Notably in our study, carotenes (α-carotene and ε-carotene) and xanthophylls (canthaxanthin and echinenone) were first identified by LC-MS/MS.

Carotenoids accumulate in many flowers, fruits, vegetables, and grains, and their quantity and composition exhibit significant species- and variety-specific variations [15,20,27,28,29,30,31,32]. Several studies have determined the carotenoid concentration in potato tubers, consisting of mainly violaxanthin, lutein, zeaxanthin, and antheraxanthin. Lutein and zeaxanthin serve as major pigments of the yellow spot in the human retina [33,34], indicating that potatoes represent a valuable source of these compounds. It is noteworthy that carotenoid content closely correlates with factors such as potato ploidy, taxonomy, and flesh color [12,35,36,37], as well as environmental and geographical conditions of their cultivation [8]. In *S. phureja* genotypes characterized by deep yellow flesh, they often display elevated levels of total carotenoid content, predominantly comprising zeaxanthin, antheraxanthin, and lutein, in contrast to those with yellow or light-yellow flesh, which show moderate levels featuring violaxanthin and antheraxanthin as the principal components. White-fleshed genotypes typically exhibit very low tuber carotenoid concentrations; lutein, violaxanthin, and β-carotene are the predominant carotenoids [14]. In addition, in a collection of ten tetraploid (*Solanum tuberosum*) and diploid (*S. phureja* and *S. chacoense*) genotypes, yellow flesh has high levels of epoxy-xanthophylls (antheraxanthin, violaxanthin, and neoxanthin) and xanthophyll esters, while white flesh has very low tuber carotenoid content, comprising tetraploid Daifla and diploid *S. chacoense* [12]. In sixty potato cultivars, including *S. tuberosum* and *S. phureja*, where most of the flesh is yellow, the dominant carotenoid was violaxanthin [10]. In *S. tuberosum* accessions, lutein and violaxanthin were the major carotenoids, and yellow-fleshed cultivars are reported to contain 58–175 mg 100 g^−1^ FW, while white-fleshed cultivars have 38–62 mg 100 g^−1^ FW total carotenoids [26]. However, it is difficult to differentiate between white- and yellow-fleshed cultivars [14]. In our study, two genotypes of *S. tuberosum* revealed that yellow-fleshed genotypes were characterized by high levels of individual carotenoid and xanthophyll esters with violaxanthin as the dominant component, whereas white-fleshed types exhibited low concentrations of individual carotenoid and xanthophyll esters with lutein as the predominant compound. Furthermore, it was shown that the different patterns of individual carotenoid accumulation between these two genotypes existed in the early stage of tuber development as well as throughout the whole development period. Additionally, there was a decline in the average values of main carotenoids, including violaxanthin, antheraxanthin, lutein, and zeaxanthin, throughout the tuber development. In contrast, xanthophyll esters displayed markedly different accumulation patterns: white-fleshed types maintained stable low levels throughout the tuber development, while yellow-fleshed types exhibited an increase in type and content of xanthophyll esters as the tuber continued to grow.

The esterification of xanthophylls to fatty acids positively influences total carotenoid accumulation by enhancing their packaging into special structures within plastids, called plastoglobules [15]. Identifying individual xanthophyll ester species remains a notoriously challenging task, and progress in this area has been slow [38]. The first report on carotenoid esters was in 1980 from *S. tuberosum* by Fishwick and Wright, who found 17–22% of the carotenoids to be present in an esterified form [16]. Breithaupt and Bamedi (2002) identified violaxanthin, lutein, and zeaxanthin as the main esterified carotenoids in potato tubers (*S. tuberosum* cv. Nicola), while the individual xanthophyll esters were not investigated [26]. In other studies, xanthophyll esters were identified as monoesters and diesters, and the individual xanthophyll esters remained uncharacterized [10,22]. Recently, by LC-MS methods, 12 individual xanthophyll esters have been identified in ten potato genotypes in tetraploid (*S. tuberosum*) and diploid (*S. phureja* and *S. chacoense*) [12]. In our study, we successfully identified 15 individual xanthophyll esters in potatoes. Among them, lutein dilaurate, lutein palmitate, violaxanthin dibutyrate, violaxanthin dilaurate, violaxanthin dipalmitate, violaxanthin laurate, violaxanthin palmitate, violaxanthin-myristate-laurate, and violaxanthin-myristate-palmitate were identified for the first time. Additionally, similar to the findings by Breithaupt and Bamedi (2002) [26], lutein, zeaxanthin, and antheraxanthin were found to be the main parent carotenoids. Previous reports have indicated that myristic and palmitic acids are the main fatty acids bound to carotenoids [12,26]. However, in this study, in addition to myristic and palmitic acids, laurate and butyrate were also found to be bound to the carotenoids. Furthermore, the content profiles of fatty acids were analyzed, and a correlation between the main fatty acid and xanthophyll esters was investigated. There were no relations between the fatty acids and individual xanthophyll esters, which also indicates that the discrepancy in the amount of fatty acids in yellow- and white-fleshed potatoes is not the determinant of xanthophyll esterification.

Recently, an experiment conducted by Li et al [17]. proposed an integrated model of rapeseed (*Brassica juncea* L.) yellow petal coloration, which is regulated by both xanthophyll esterification and plastoglobule formation. Potatoes rank among the most important staple crops globally, providing significant caloric intake for millions of people. In potatoes, the yellow color is attributed to xanthophylls, with their esterification directly correlating to accumulation levels [10]. Our study found that xanthophyll esterification in yellow-fleshed potatoes was more pronounced than in white-fleshed potatoes; as the tuber developed, both the variety and content increased. The number of xanthophyll esters in yellow-fleshed potatoes was 5, 6, 9, and 10 times more than in white-fleshed potatoes through the second to fifth development stage, respectively. This indicates that yellow-fleshed genotypes tend to store carotenoids in a more stable form. These findings align with our visual perception.

Flesh color plays a significant role in determining the flavor and aroma of potatoes, which in turn captures consumer interest in the marketplace. At present, yellow-fleshed potatoes are more favored among consumers within the vegetable market. A direct correlation between the carotenoid content and the xanthophyll esterified fraction was observed, suggesting that the esterification process facilitates the accumulation of these lipophilic compounds within the plastids [10]. In potatoes, the yellow color is given by xanthophylls, with esterification directly correlating to their accumulation [10]. As we know, the color parameter a* represents red and green, b* represents yellow and blue, C* represents saturation, and H° represents hue angle. Thus, the color measurement is an appropriate method for rapid estimation [39]. In some studies, the correlation between the content of carotenoids and tuber color was analyzed [40,41]. The a*, b*, H°, and CCI values showed a certain correlation with the content of some carotenoids [40]. Notably, our results indicated that violaxanthin had significant negative correlations with a* while showing strong positive correlations with b*, H°, and C*, which may suggest it plays an important role in forming yellow flesh. In addition, lutein, zeaxanthin, antheraxanthin, violaxanthin myristate, and lutein myristate exhibited moderate correlations with b*, H°, and C*; these findings imply their contribution to the yellow flesh coloration of potatoes.

## 5. Conclusions

In our study, 22 carotenoids were identified in yellow-fleshed potatoes whereas 18 were detected in white-fleshed potatoes by LC-MS/MS. Lutein, violaxanthin, zeaxanthin, antheraxanthin, neoxanthin, and both violaxanthin myristate and lutein myristate were consistently detected at all five developmental stages in both genotypes, and their levels were significantly higher in the yellow-fleshed genotype compared to those in the white-fleshed genotype. Furthermore, xanthophyll esterification in yellow-fleshed potatoes was more pronounced than in white-fleshed potatoes as the tubers developed. Beyond that, violaxanthin, lutein, zeaxanthin, antheraxanthin, violaxanthin myristate, and lutein myristate exhibited significant or moderate correlations with b*, H°, and C*, which demonstrated their contributions to the yellow flesh coloration of potatoes. Our findings imply that, compared to white-fleshed potatoes, yellow-fleshed potatoes contain a higher level of individual carotenoids in a more stable form, indicating their potential advantages for human health by providing enhanced carotenoid availability.

## Figures and Tables

**Table 1:** The color indexes (L*, a*, b*, C*, H°) of two potato genotypes in five developmental stages.

	L*	a*	b*	C*	H°
WS1	54.79 ± 1.13 bc	0.56 ± 0.08 cd	5.06 ± 0.04 f	5.09 ± 0.05 f	83.66 ± 0.79 c
WS2	52.76 ± 0.95 cd	0.78 ± 0.05 b	4.19 ± 0.05 h	4.26 ± 0.06 h	79.44 ± 0.57 d
WS3	57.06 ± 0.72 ab	0.82 ± 0.06 ab	4.69 ± 0.12 g	4.76 ± 0.12 g	80.12 ± 0.48 d
WS4	49.96 ± 0.86 e	0.79 ± 0.01 b	4.37 ± 0.05 h	4.44 ± 0.05 h	79.78 ± 0.21 d
WS5	57.73 ± 0.83 a	0.95 ± 0.04 a	5.04 ± 0.07 f	5.12 ± 0.06 f	79.32 ± 0.62 d
YS1	51.01 ± 0.36 de	0.56 ± 0.02 cd	6.97 ± 0.09 e	6.99 ± 0.09 e	85.38 ± 0.12 b
YS2	52.81 ± 1.04 cd	0.36 ± 0.06 e	9.83 ± 0.09 a	9.84 ± 0.09 a	87.90 ± 0.34 a
YS3	56.10 ± 0.82 ab	0.63 ± 0.04 cd	8.32 ± 0.12 c	8.34 ± 0.12 c	85.69 ± 0.18 b
YS4	52.32 ± 0.55 cde	0.54 ± 0.02 d	8.62 ± 0.05 b	8.63 ± 0.05 b	86.38 ± 0.12 b
YS5	56.77 ± 0.64 ab	0.69 ± 0.01 bc	7.97 ± 0.07 d	8.00 ± 0.07 d	85.03 ± 0.10 b

Values are the means ± SE of three determinations. A one-way balanced ANOVA followed by Duncan’s test was performed. Lowercase letters represent significance at *p* ≤ 0.05.

**Table 2:** Carotenoids in yellow and white fleshed potatoes at five developmental stages identified by LC-MS/MS.

Peak Number	Retention Time (min)	Tentative Assignment	Q1 (Da)	Q3 (Da)	Ionization Model	Molecular Weight
1	1.58	violaxanthin	601.4	221	[M+H]+	600.4179
2	1.95	neoxanthin	601.4	565.5	[M+H]+	600.4179
3	2.88	antheraxanthin	585.5	175.4	[M+H]+	584.4229
4	4.06	lutein	551.5	175.4	[M+H-18]+	568.428
5	4.64	zeaxanthin	569.4	477.5	[M+H]+	568.428
6	4.75	canthaxanthin	565.5	203.3	[M+H]+	564.8
7	5.53	ε-carotene	537.6	123.2	[M+H]+	536.438232
8	5.55	echinenone	551.6	203.1	[M+H]+	550.9
9	5.69	violaxanthin laurate	783.7	583.4	[M+H-18]+	800.7
10	5.92	α-carotene	537.5	123.2	[M+H]+	536.438232
11	5.99	violaxanthin myristate	811.8	793.7	[M+H]+	810.8
12	6.22	violaxanthin dibutyrate	741.6	653.5	[M+H]+	740.6
13	6.28	β-carotene	537.6	177.1	[M+H]+	536.4
14	6.28	violaxanthin palmitate	839.8	821.8	[M+H]+	838.8
15	6.59	lutein myristate	761.8	533.5	[M+H-18]+	778.8
16	6.64	violaxanthin dilaurate	966.7	948.8	[M+H]+	965.7
17	6.82	violaxanthin-myristate-laurate	993.8	975.7	[M+H]+	992.8
18	6.95	lutein palmitate	789.8	533.5	[M+H-18]+	806.8
19	7.01	violaxanthin dimyristate	1021.8	793.7	[M+H]+	1020.8
20	7.18	lutein dilaurate	733.5	533.3	[M+H-201]+	933.5
21	7.22	violaxanthin-myristate-palmitate	1050	793.8	[M+H]+	1049
22	7.35	lutein dimyristate	761.8	533.5	[M+H-228]+	988.8
23	7.39	violaxanthin dipalmitate	1077.9	821.7	[M+H]+	1076.9
24	7.63	zeaxanthin dimyristate	990	761.8	[M+H]+	989
25	7.95	zeaxanthin dipalmitate	789.5	533.5	[M+H-256]+	1045.1

**Table 3:** Individual carotenoid contents of the yellow- and white-fleshed genotypes at five developmental stages.

Compound Number	Individual Carotenoid	WS1	WS2	WS3	WS4	WS5	YS1	YS2	YS3	YS4	YS5
1	α-carotene	4.04 ± 1.45 a	nd	nd	nd	0.96 ± 0.24 b	nd	nd	nd	nd	2.43 ± 0.62 ab
2	β-carotene	18.24 ± 1.06 a	10.17 ± 2.69 b	3.12 ± 0.54 c	3.14 ± 0.55 c	5.37 ± 0.42 c	9.91 ± 1.37 b	5.71 ± 1.27 c	1.71 ± 0.27 c	nd	2.93 ± 0.74 c
3	ε-carotene	0.37 ± 0.07	0.35 ± 0.08	nd	nd	nd	nd	nd	nd	nd	nd
4	lutein	371.93 ± 34.5 ab	215.55 ± 23.21 d	165.66 ± 8.97 d	148.79 ± 18.22 d	184.80 ± 21.65 d	430.06 ± 4.31 a	298.03 ± 32.09 c	286.75 ± 9.26 c	311.64 ± 26.15 bc	375.10 ± 22.38 ab
5	violaxanthin	197.26 ± 13.19 c	139.40 ± 27.43 cd	77.44 ± 4.14 cd	46.83 ± 7.61 d	39.12 ± 3.27 d	679.97 ± 84.14 a	714.58 ± 55.96 a	386.02 ± 14.00 b	340.61 ± 24.12 b	399.08 ± 52.74 b
6	antheraxanthin	12.71 ± 1.76 a	7.33 ± 0.97 a	12.18 ± 2.81 a	17.02 ± 3.50 a	8.78 ± 0.96 a	39.35 ± 5.47 b	29.33 ± 12.27 bc	75.99 ± 9.06 a	94.95 ± 4.4 a	81.68 ± 11.45 a
7	zeaxanthin	6.53 ± 0.99 cd	4.20 ± 0.91 de	3.67 ± 0.24 e	3.34 ± 0.58 e	4.08 ± 1.48 de	10.99 ± 0.44 a	4.32 ± 0.83 de	8.88 ± 0.60 abc	10.01 ± 0.97 ab	7.33 ± 0.46 bc
8	neoxanthin	41.35 ± 3.86 c	19.93 ± 3.52 d	10.24 ± 1.11 de	7.99 ± 0.54 e	5.60 ± 0.23 e	87.63 ± 6.06 a	67.36 ± 6.88 b	37.96 ± 0.44 c	32.96 ± 3.07 c	34.56 ± 3.55 c
9	canthaxanthin	nd	nd	nd	nd	nd	nd	0.05 ± 0.02	nd	nd	nd
10	echinenone	0.18 ± 0.03	nd	nd	0.06 ± 0.02	nd	0.08 ± 0.04	0.12 ± 0.08	nd	nd	nd
11	violaxanthin myristate	49.42 ± 5.33 b	26.07 ± 3.04 de	23.59 ± 2.82 de	14.25 ± 2.07 e	14.13 ± 0.15 e	70.80 ± 3.09 a	42.77 ± 5.16 bc	34.32 ± 6.32 cd	40.09 ± 3.12 bc	53.09 ± 3.22 b
12	lutein myristate	1.18 ± 0.26 d	0.89 ± 0.15 d	2.26 ± 0.39 cd	1.54 ± 0.62 cd	2.38 ± 0.61 cd	2.41 ± 0.29 cd	2.65 ± 0.82 cd	4.95 ± 1.63 bc	6.93 ± 0.35 ab	9.70 ± 2.10 a
13	lutein dimyristate	nd	0.39 ± 0.01 b	0.57 ± 0.265 b	nd	nd	1.19 ± 0.21 b	2.76 ± 0.92 b	12.11 ± 2.96 a	12.76 ± 0.47 a	14.10 ± 2.62 a
14	violaxanthin-myristate-laurate	2.14 ± 0.67 d	nd	nd	nd	nd	21.19 ± 0.88 bc	14.21 ± 5.66 cd	32.15 ± 7.18 ab	36.72 ± 1.97 a	42.79 ± 4.16 a
15	lutein palmitate	0.59 ± 0.19 c	0.35 ± 0.11 c	0.76 ± 0.09 bc	nd	nd	0.63 ± 0.17 c	2.07 ± 0.54 b	1.13 ± 0.45 bc	3.56 ± 0.4 a	3.41 ± 0.74 a
16	violaxanthin dimyristate	nd	nd	nd	nd	nd	4.41 ± 0.06 b	7.78 ± 3.52 b	23.38 ± 5.59 a	26.87 ± 3.24 a	36.99 ± 5.62 a
17	violaxanthin palmitate	nd	nd	nd	nd	nd	nd	26.00 ± 8.52 b	38.85 ± 2.85 b	75.76 ± 8.72 a	77.03 ± 1.33 a
18	violaxanthin-myristate-palmitate	nd	nd	nd	nd	nd	nd	12.48 ± 5.16 c	25.04 ± 0.44 b	38.62 ± 1.20 a	42.10 ± 5.51 a
19	violaxanthin dipalmitate	nd	nd	nd	nd	nd	nd	9.83 ± 1.78 c	18.46 ± 1.11 b	28.82 ± 0.25 a	25.80 ± 2.02 a
20	zeaxanthin dimyristate	nd	nd	nd	nd	nd	nd	nd	0.62 ± 0.16 b	1.07 ± 0.13 ab	1.42 ± 0.55 a
21	lutein dilaurate	nd	nd	nd	nd	nd	nd	0.29 ± 0.02 b	1.27 ± 0.22 b	0.45 ± 0.14 b	2.39 ± 0.17 a
22	violaxanthin laurate	1.26 ± 0.77 ab	0.45 ± 0.07 b	0.21 ± 0.08 b	nd	nd	1.61 ± 0.06 a	nd	nd	nd	nd
23	violaxanthin dibutyrate	2.00 ± 1.04	nd	0.65 ± 0.03	nd	nd	nd	nd	nd	nd	nd
24	violaxanthin dilaurate	nd	nd	nd	nd	nd	nd	nd	2.81 ± 1.75	nd	1.64 ± 0.79
25	zeaxanthin dipalmitate	nd	nd	nd	0.15 ± 0.03	nd	nd	nd	nd	nd	nd

Values are the means ± SE of three determinations. A one-way balanced ANOVA followed by Duncan’s test was performed. Lowercase letters represent significance at *p* ≤ 0.05. nd, not detected.

**Table 4:** Fatty acid contents of yellow and white flesh potato genotypes at five developmental stages.

Index	Compounds	WS1	WS2	WS3	WS4	WS5	YS1	YS2	YS3	YS4	YS5
C10-0	decanoic acid	0.07 ± 0.006 a	0.05 ± 0.003 bc	0.04 ± 0.001 c	0.04 ± 0.001 c	0.04 ± 0.002 c	0.05 ± 0.006 bc	0.06 ± 0.007 ab	0.04 ± 0.003 c	0.05 ± 0.01 bc	0.04 ± 0.002 c
C16-1T	trans-9-palmitelaidic acid	nd	0.20 ± 0.20	nd	nd	nd	nd	0.06 ± 0.06	nd	nd	nd
C18-2n6t	linolelaidic acid	0.50 ± 0.09 a	0.14 ± 0.005 b	0.14 ± 0.02 b	0.13 ± 0.002 b	0.14 ± 0.003 b	0.54 ± 0.02 a	0.15 ± 0.01 b	0.17 ± 0.01 b	0.16 ± 0.006 b	0.16 ± 0.004 b
C9-0	nonanoic acid	0.49 ± 0.11 a	0.15 ± 0.01 b	0.12 ± 0.009 b	0.13 ± 0.003 b	0.13 ± 0.006 b	0.15 ± 0.01 b	0.18 ± 0.01 b	0.11 ± 0.02 b	0.17 ± 0.04 b	0.11 ± 0.006 b
C8-0	octanoic acid	0.48 ± 0.03 a	0.19 ± 0.007 cd	0.14 ± 0.01 de	0.12 ± 0.02 e	0.11 ± 0.006 e	0.21 ± 0.03 bc	0.24 ± 0.03 b	0.15 ± 0.001 de	0.17 ± 0.02 cde	0.11 ± 0.009 e
C11-0	hendecanoic acid	0.03 ± 0.002 abc	0.02 ± 0.002 bc	0.02 ± 0.004 bc	0.03 ± 0.002 abc	0.02 ± 0.002 c	0.02 ± 0.005 bc	0.04 ± 0.01 a	0.02 ± 0.003 bc	0.04 ± 0.01 ab	0.03 ± 0.002 abc
C12-0	lauric acid	0.22 ± 0.01 ab	0.20 ± 0.008 abcd	0.16 ± 0.001 cd	0.17 ± 0.03 bcd	0.18 ± 0.02 abcd	0.21 ± 0.03 abc	0.23 ± 0.02 a	0.15 ± 0.001 d	0.21 ± 0.02 abc	0.17 ± 0.01 bcd
C13-0	tridecanoic acid	0.07 ± 0.004 a	0.06 ± 0.003 b	0.06 ± 0.002 b	0.06 ± 0.001 b	0.06 ± 0.004 b	0.06 ± 0.003 a	0.06 ± 0.001 b	0.06 ± 0.003 b	0.06 ± 0.004 b	0.06 ± 0.001 b
C14-0	myristic acid	3.23 ± 0.03 bc	3.50 ± 0.16 b	2.91 ± 0.01 bc	2.69 ± 0.43 bc	2.32 ± 0.40 c	3.69 ± 0.37 bc	4.63 ± 0.64 a	2.99 ± 0.26 bc	3.14 ± 0.18 bc	2.92 ± 0.2 bc
C15-0	pentadecanoic acid	1.06 ± 0.01 a	0.50 ± 0.02 c	0.56 ± 0.03 c	0.46 ± 0.04 c	0.49 ± 0.02 c	0.78 ± 0.12 b	0.58 ± 0.07 c	0.42 ± 0.03 c	0.46 ± 0.05 c	0.45 ± 0.02 c
C16-0	palmitic acid	270.14 ± 4.57 a	200.86 ± 4.43 cd	167.23 ± 5.11 a	164.80 ± 3.73 a	151.16 ± 4.96 a	238.58 ± 10.3 b	218.28 ± 16.70 bc	158.63 ± 5.01 a	177.08 ± 17.62 de	151.98 ± 7.20 a
C17-0	heptadecanoic acid	1.91 ± 0.06 a	0.98 ± 0.03 cd	0.93 ± 0.03 a	0.86 ± 0.01 a	0.82 ± 0.02 a	1.64 ± 0.13 b	1.18 ± 0.17 c	0.80 ± 0.03 a	0.93 ± 0.07 a	0.78 ± 0.03 a
C18-0	stearic acid	130.82 ± 1.75 a	106.66 ± 5.78 bc	101.56 ± 0.79 bc	103.22 ± 3.10 bc	92.12 ± 3.81 c	105.20 ± 6.35 bc	125.68 ± 7.30 a	92.97 ± 1.76 c	110.76 ± 8.63 b	92.76 ± 3.71 c
C18-1n9c	cis-9-octadecenoic acid	8.40 ± 1.08 b	3.40 ± 0.74 c	2.43 ± 0.38 c	2.48 ± 0.11 c	2.23 ± 0.26 c	12.56 ± 1.86 a	3.83 ± 0.90 c	4.39 ± 0.66 c	4.17 ± 0.59 c	3.34 ± 0.61 c
C18-1n9t	trans-9-octadecenoic acid	2.96 ± 0.46 a	1.45 ± 0.05 bc	1.06 ± 0.13 c	1.12 ± 0.07 bc	1.25 ± 0.07 bc	3.30 ± 0.15 a	1.73 ± 0.22 b	1.61 ± 0.17 bc	1.74 ± 0.13 b	1.44 ± 0.10 bc
C18-2n6c	linoleic acid	21.74 ± 7.01 b	2.92 ± 0.23 c	5.55 ± 0.58 c	3.86 ± 0.20 c	4.23 ± 0.19 c	34.52 ± 6.43 a	5.10 ± 0.45 c	6.87 ± 0.88 c	7.08 ± 0.55 c	7.49 ± 0.43 c
C18-3n3	α-linolenic acid	11.04 ± 3.09a	1.23 ± 0.08 b	1.52 ± 0.25 b	1.08 ± 0.06 b	1.32 ± 0.04 b	12.86 ± 2.18 a	1.39 ± 0.15 b	1.74 ± 0.23 b	1.92 ± 0.23 b	2.10 ± 0.19 b
C19-0	nonadecylic acid	0.35 ± 0.02	0.23 ± 0.005	0.26 ± 0.009	0.23 ± 0.002	0.25 ± 0.001	0.27 ± 0.02	0.25 ± 0.007	0.23 ± 0.004	0.24 ± 0.01	0.26 ± 0.005
C19-1(cis-10)	cis-10-carboenoic acid	0.57 ± 0.08 b	0.68 ± 0.12 ab	0.64 ± 0.07 ab	0.71 ± 0.03 ab	0.70 ± 0.05 ab	0.69 ± 0.02 ab	0.76 ± 0.09 ab	0.58 ± 0.03 b	0.87 ± 0.10 a	0.69 ± 0.07 ab
C20-0	arachidic acid	8.30 ± 0.08 a	4.27 ± 0.16 cd	4.27 ± 0.26 cd	3.53 ± 0.06 de	3.50 ± 0.12 de	7.06 ± 0.46 b	5.15 ± 0.64 c	3.45 ± 0.24 de	3.74 ± 0.32 de	3.22 ± 0.10 e
C21-0	heneicosanoic acid	0.98 ± 0.03 a	0.67 ± 0.003 c	0.72 ± 0.02 c	0.67 ± 0.005 c	0.71 ± 0.01 c	0.84 ± 0.05 b	0.71 ± 0.02 c	0.69 ± 0.005 c	0.69 ± 0.02 c	0.73 ± 0.01 c
C22-0	behenic acid	3.34 ± 0.06 a	1.47 ± 0.06 bc	1.26 ± 0.04 c	1.17 ± 0.004 c	1.15 ± 0.02 c	3.48 ± 0.29 a	1.82 ± 0.18 b	1.32 ± 0.05 c	1.49 ± 0.11 c	1.33 ± 0.02 bc
C22-1n9	erucic acid	1.23 ± 0.17 a	1.24 ± 0.15 a	1.08 ± 0.09 a	1.00 ± 0.12 a	0.95 ± 0.04 a	1.40 ± 0.35 a	1.21 ± 0.04 a	1.11 ± 0.14 a	1.46 ± 0.42 a	1.19 ± 0.02 a
C23-0	tricosanoic acid	1.65 ± 0.08 a	1.04 ± 0.02 d	1.17 ± 0.05 cd	1.07 ± 0.008 d	1.12 ± 0.006 cd	1.47 ± 0.06 b	1.24 ± 0.05 c	1.11 ± 0.02 cd	1.21 ± 0.03 c	1.22 ± 0.02 c
C24-0	lignoceric acid	4.89 ± 0.29 b	2.65 ± 0.17 d	1.97 ± 0.17 efg	1.57 ± 0.02 g	1.65 ± 0.05 fg	5.87 ± 0.07 a	3.36 ± 0.40 c	2.24 ± 0.13 def	2.32 ± 0.18 de	2.05 ± 0.04 defg
C6-0	hexanoic acid	0.56 ± 0.007 a	0.36 ± 0.003 b	0.26 ± 0.005 c	0.24 ± 0.004 c	0.25 ± 0.007 c	0.53 ± 0.04 a	0.42 ± 0.06 b	0.27 ± 0.02 c	0.27 ± 0.22 c	0.24 ± 0.006 c

Values are the means ± SE of three determinations. A one-way balanced ANOVA followed by Tukey’s test was performed. Lowercase letters represent significance at *p* ≤ 0.05. nd, not detected.

## Data Availability

The original contributions presented in this study are included in the article/Supplementary Materials. Further inquiries can be directed to the corresponding author.

## Supplementary Materials

The following supporting information can be downloaded at: https://www.mdpi.com/article/10.3390/foods13223691/s1, Figure S1: The mass spectrum of 25 carotenoids analyzed by LC-MS/MS; Figure S2: Total ion chromatogram of the WS and YS samples at five developmental stages; Table S1: Carotenoids identified in WS, YS, and in both WS and YS.
