# Supplementary material for: Comparative Analysis of Individual Carotenoid Profiles in Yellow- and White-Fleshed Potatoes (Solanum tuberosum L.) During Tuber Development
Source: Foods. 2024 Nov 20;13(22):3691. doi: 10.3390/foods13223691 (PMC11593604; doi:10.3390/foods13223691)
Supplement: Supplementary file 1 [file foods-13-03691-s001.zip › Supplementary Figure S1.pdf]

**Supplementary Figure S1. The mass spectum of 25 carotenoids analyzed by LC-MS/MS.**

α -Carotene

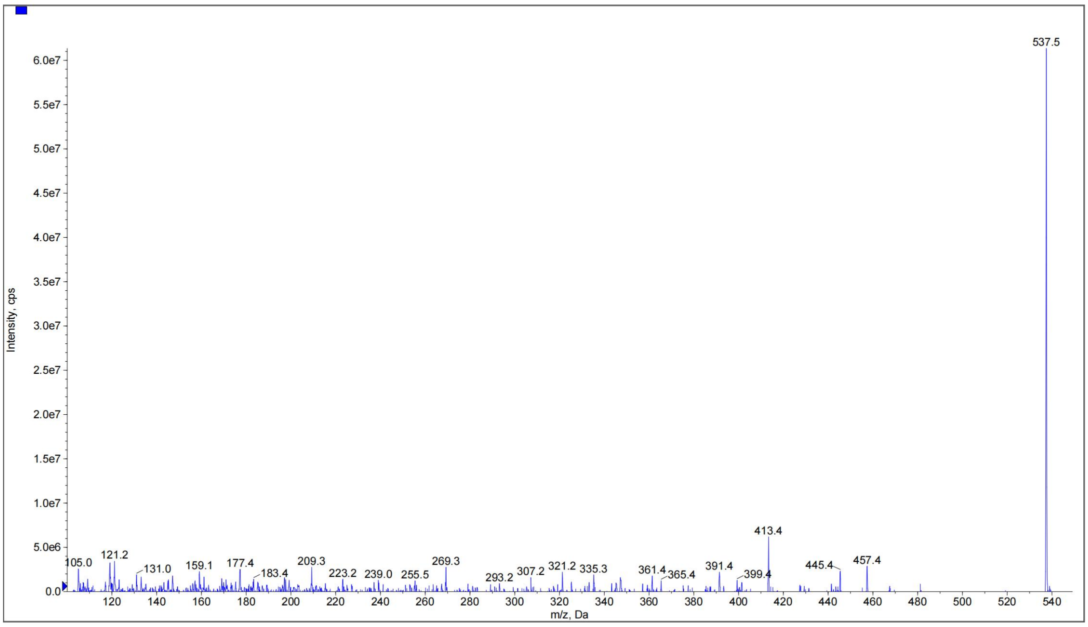


β -Carotene

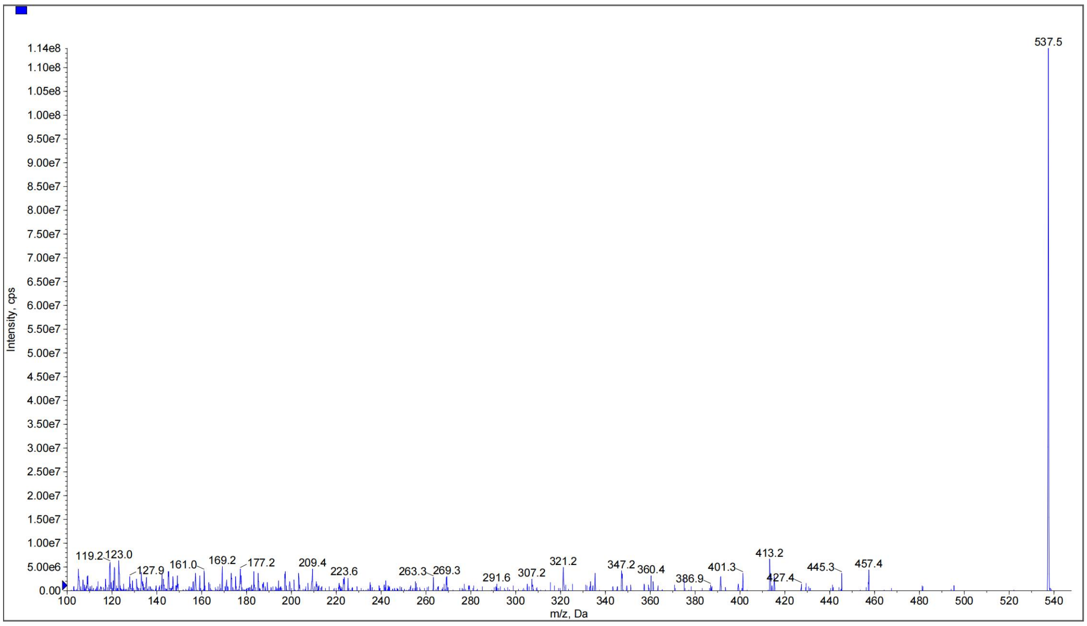

ε -Carotene

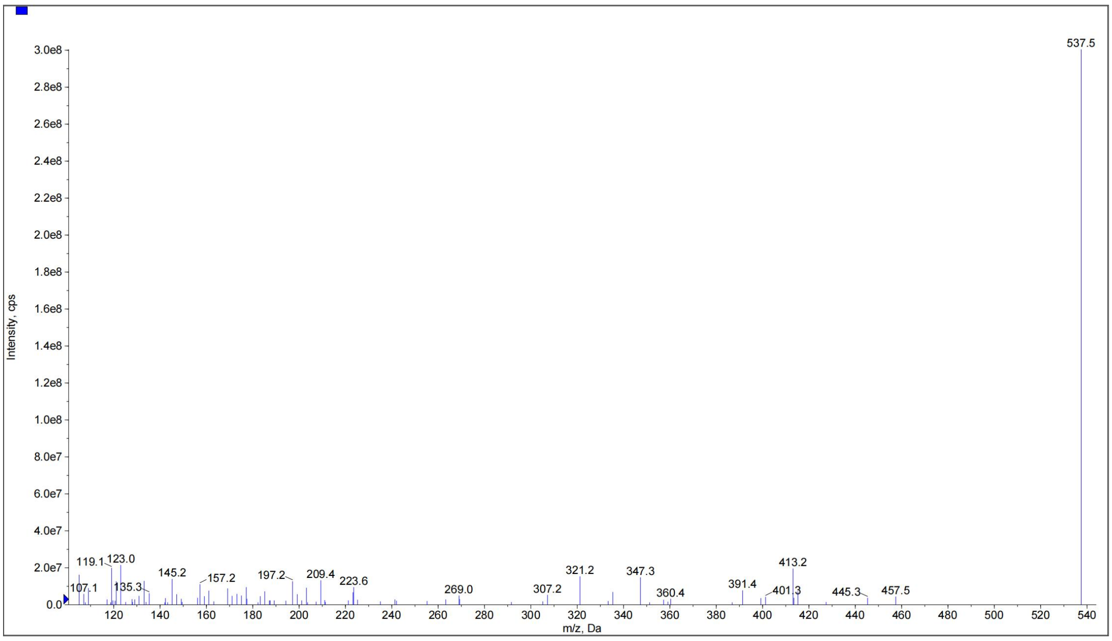


Lutein-myristate

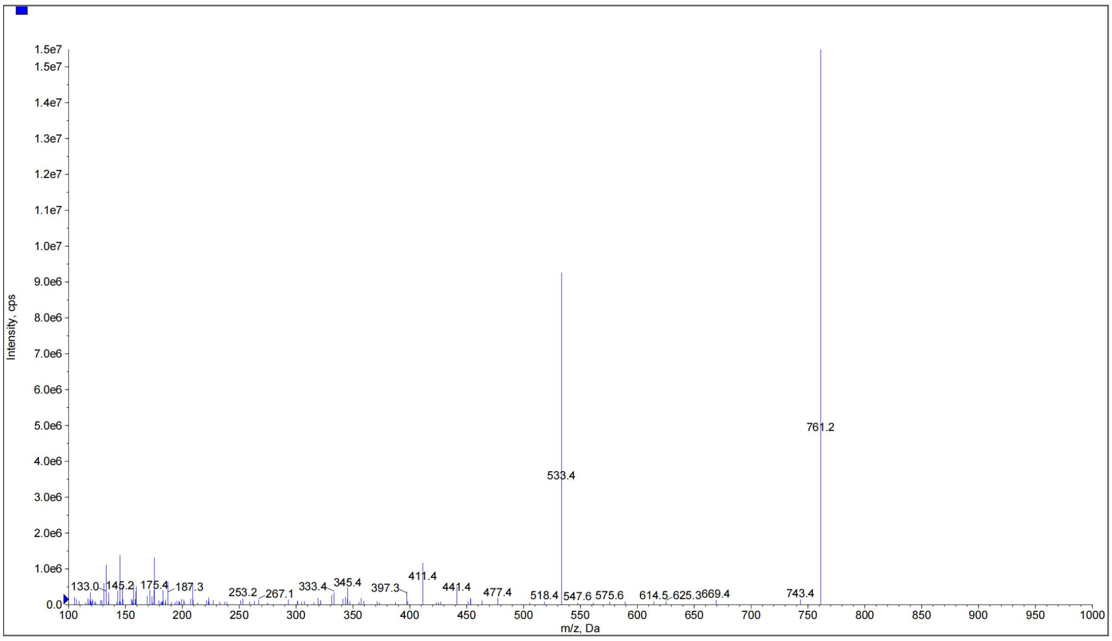

Lutein-palmitate

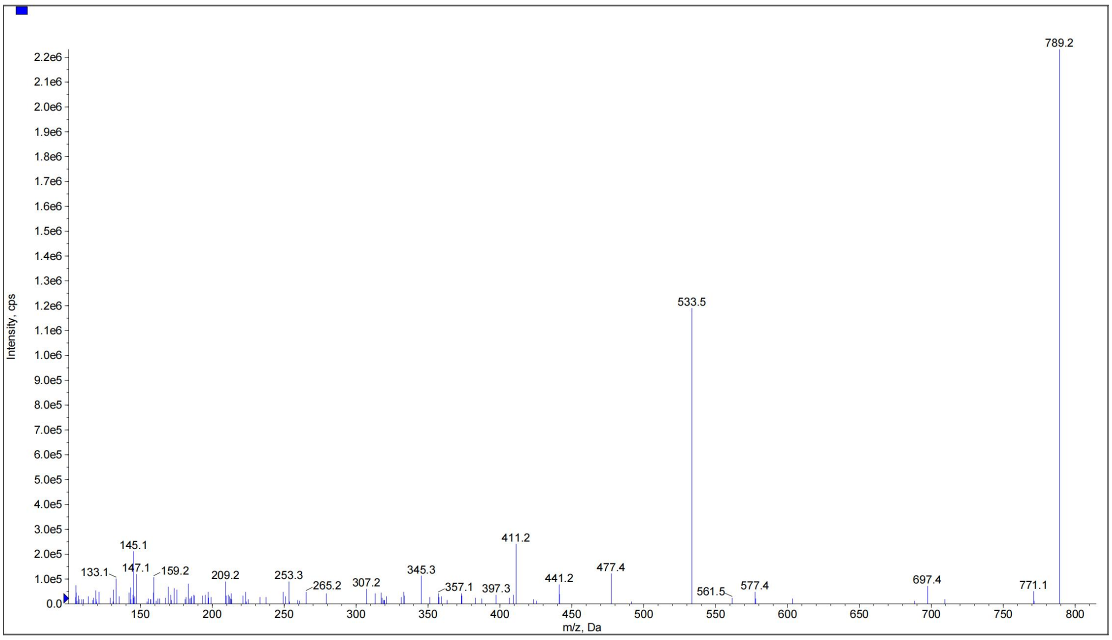


Lutein-dilaurate

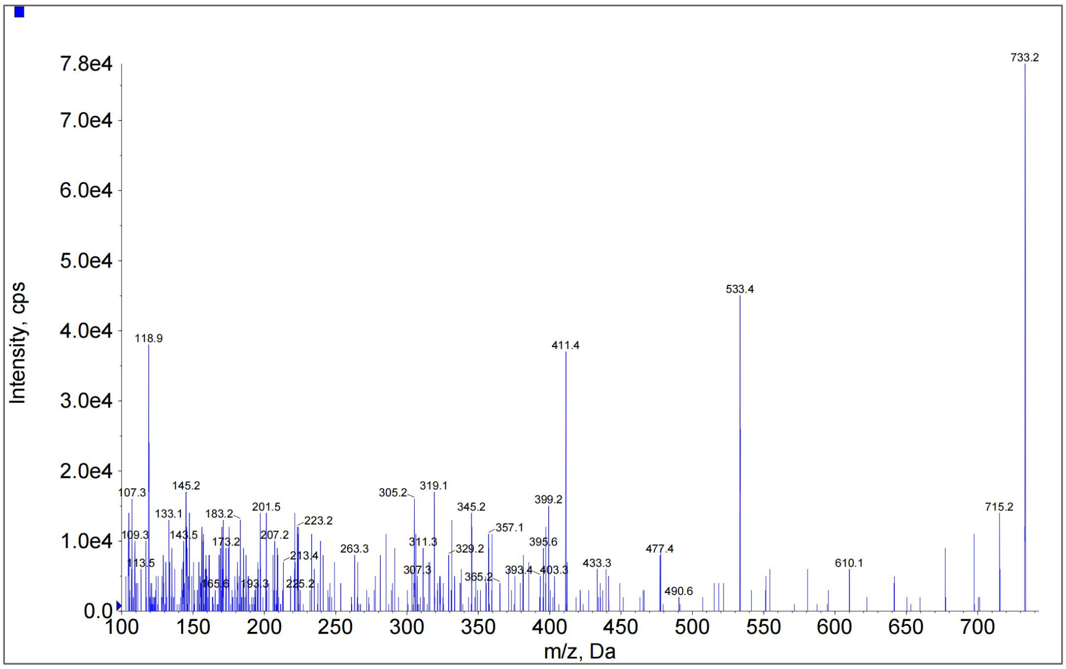

Violaxanthin-dibutyrate

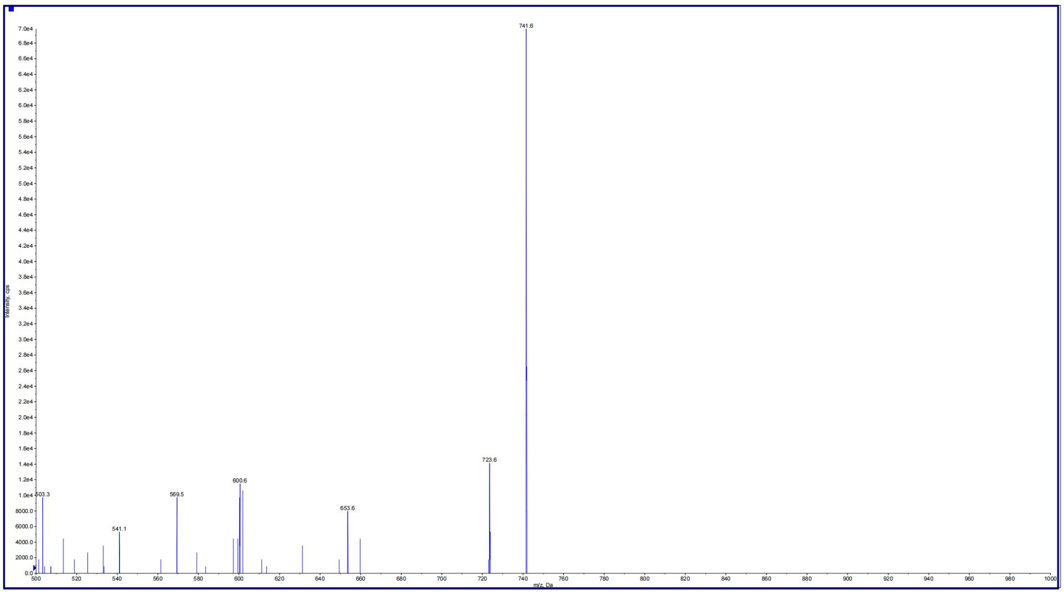


Violaxanthin-laurate

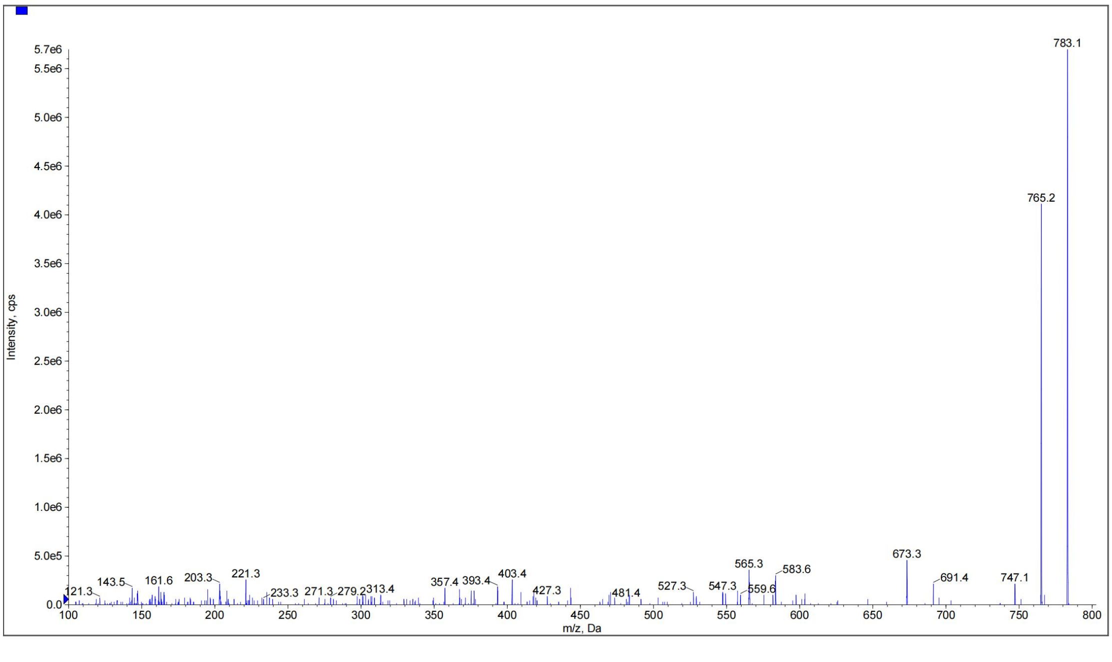

Lutein-dimyristate

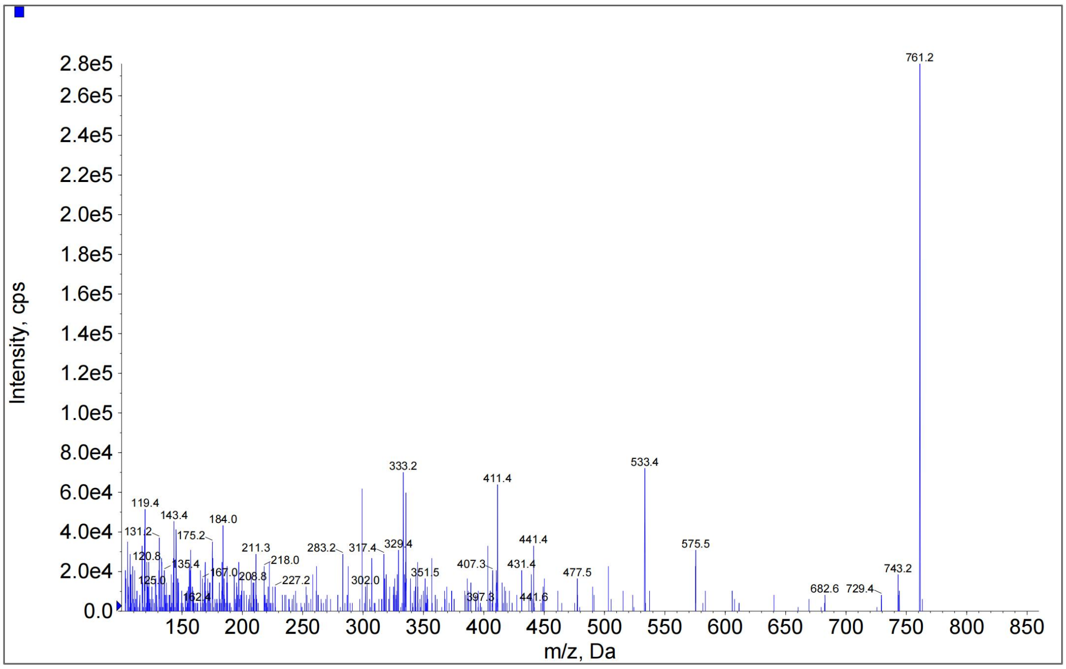


Violaxanthin-dimyristate

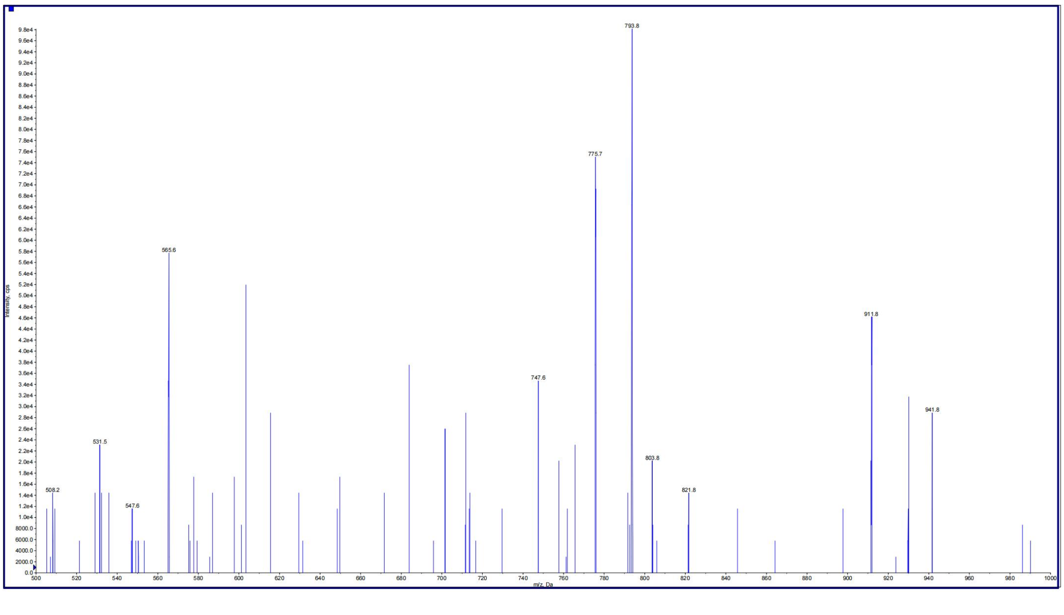

Violaxanthin-myristate

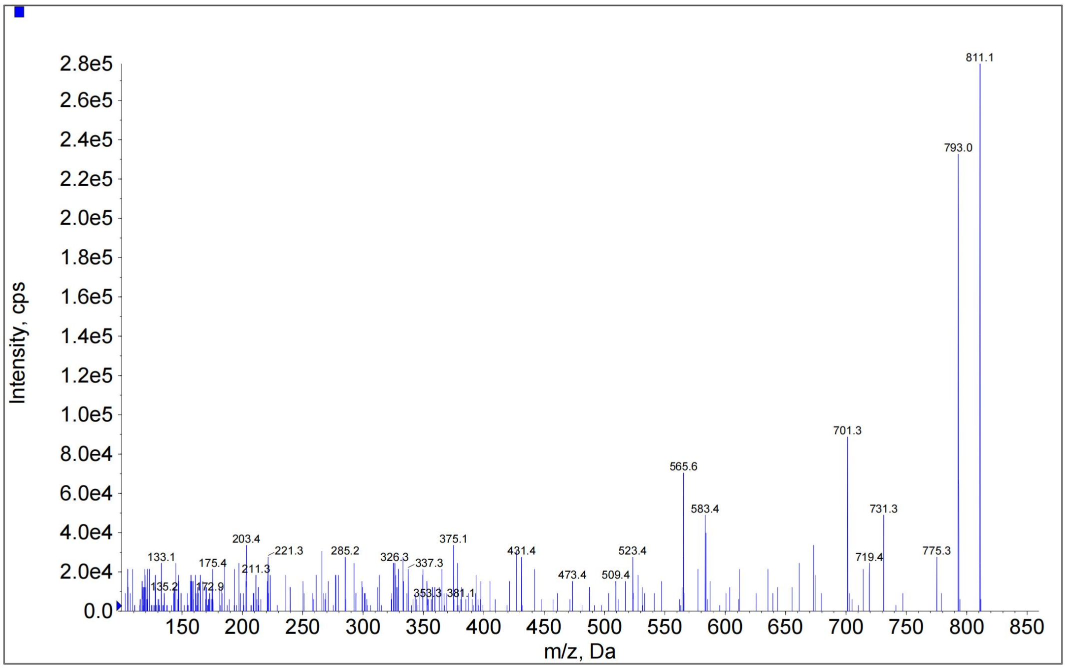


Violaxanthin-palmitate

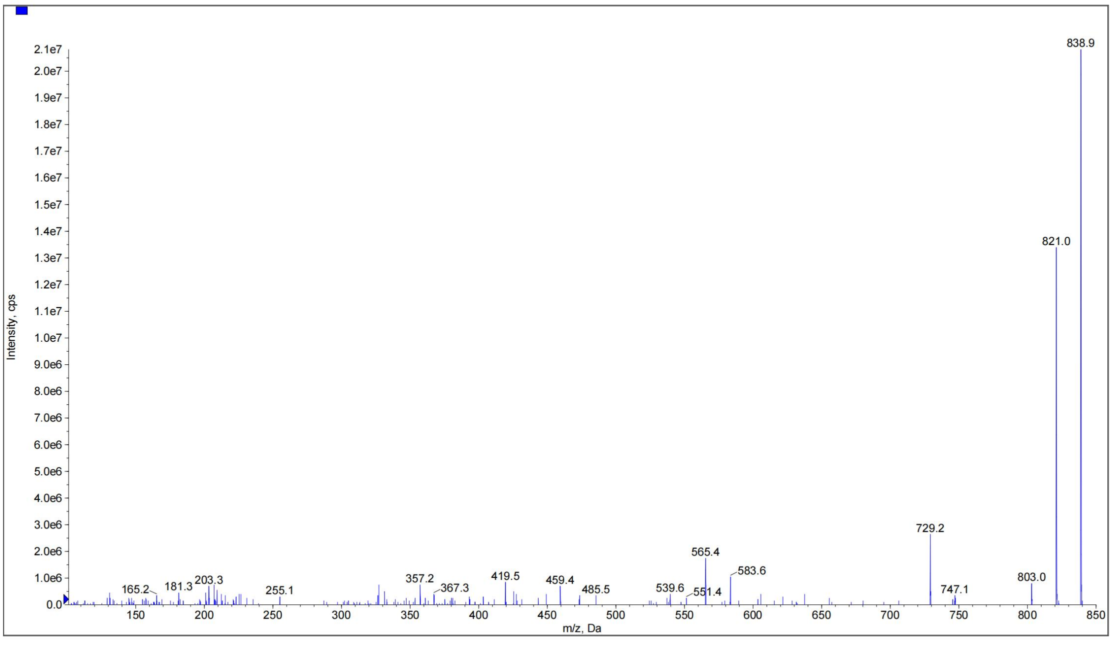

Violaxanthin-dilaurate

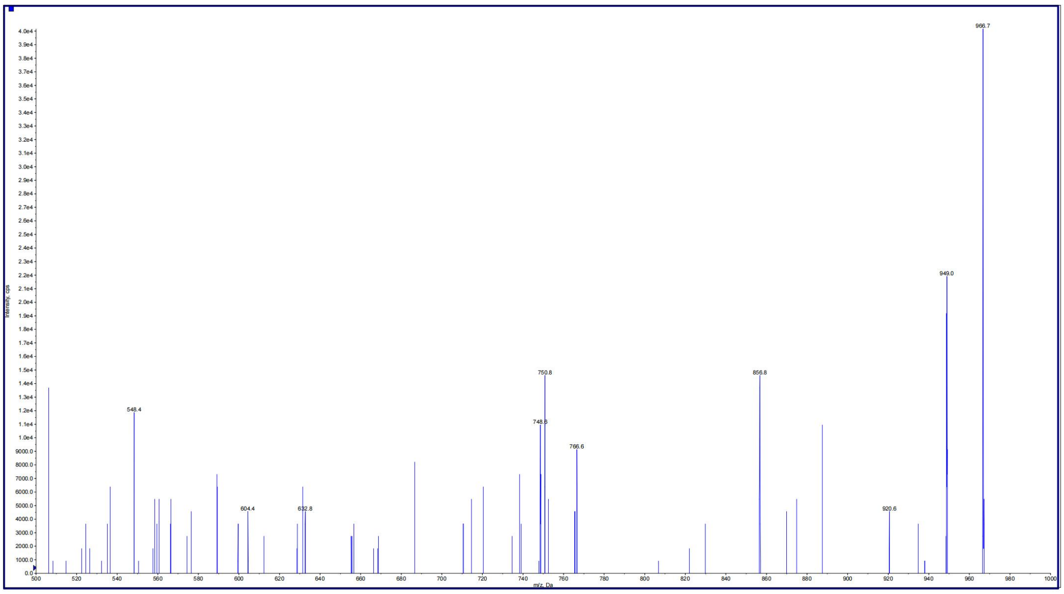


Violaxanthin-myristate-laurate

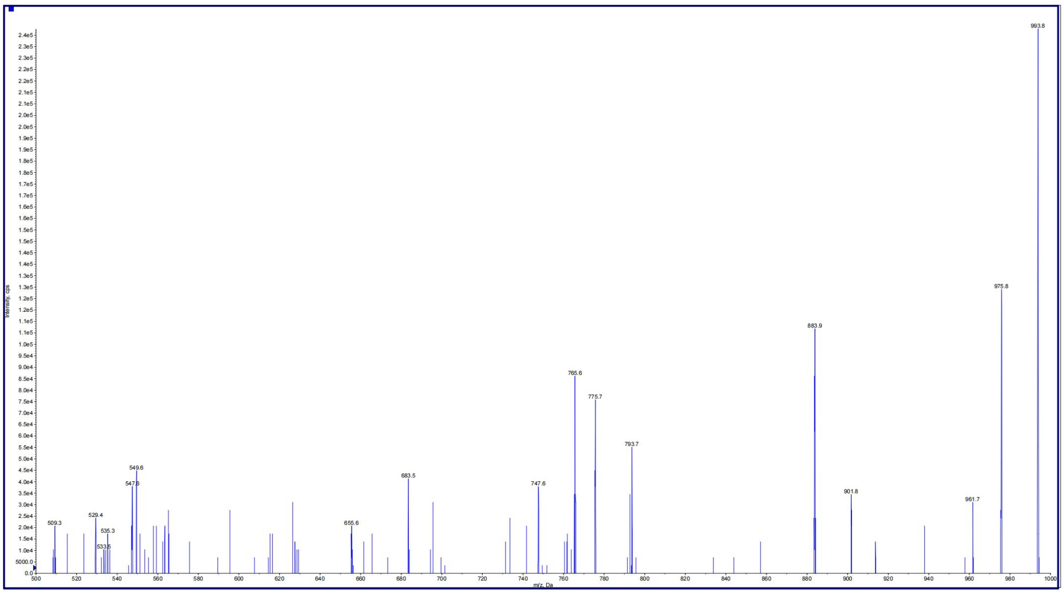

Violaxanthin-myristate-palmitate

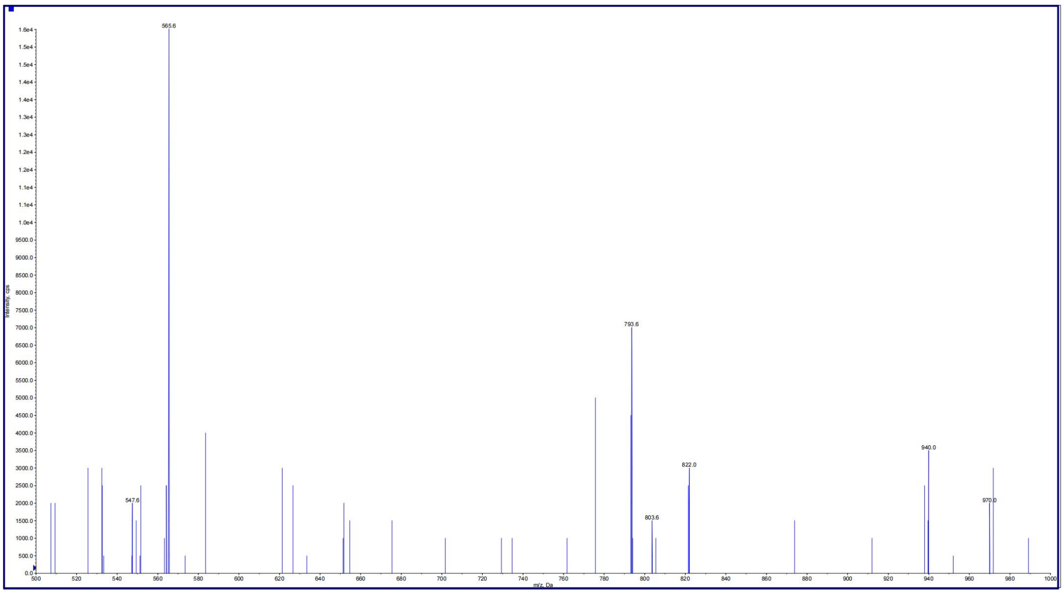


Violaxanthin-dipalmitate

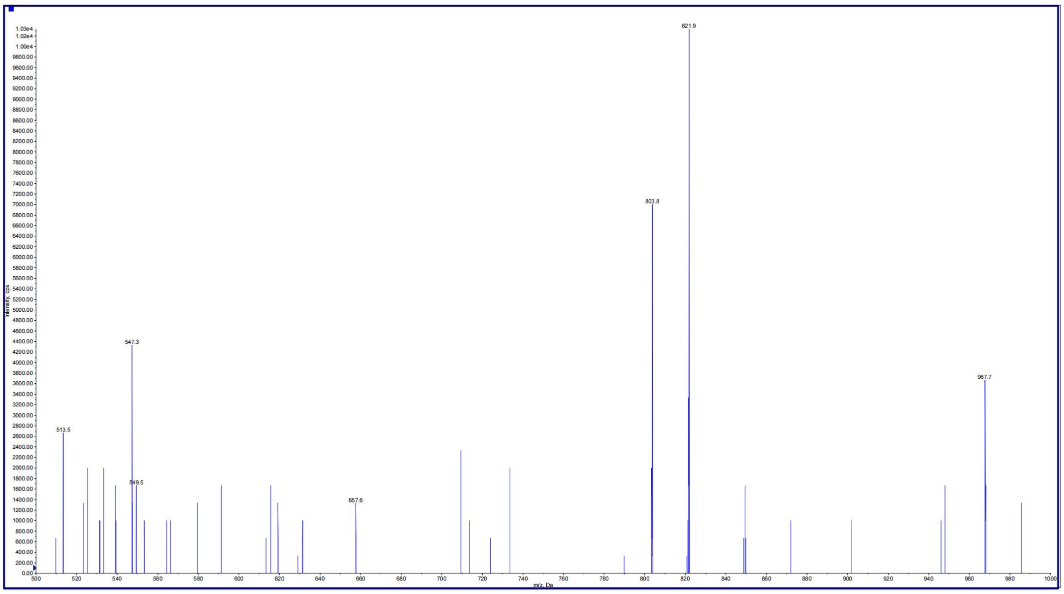

Zeaxanthin-dimyristate

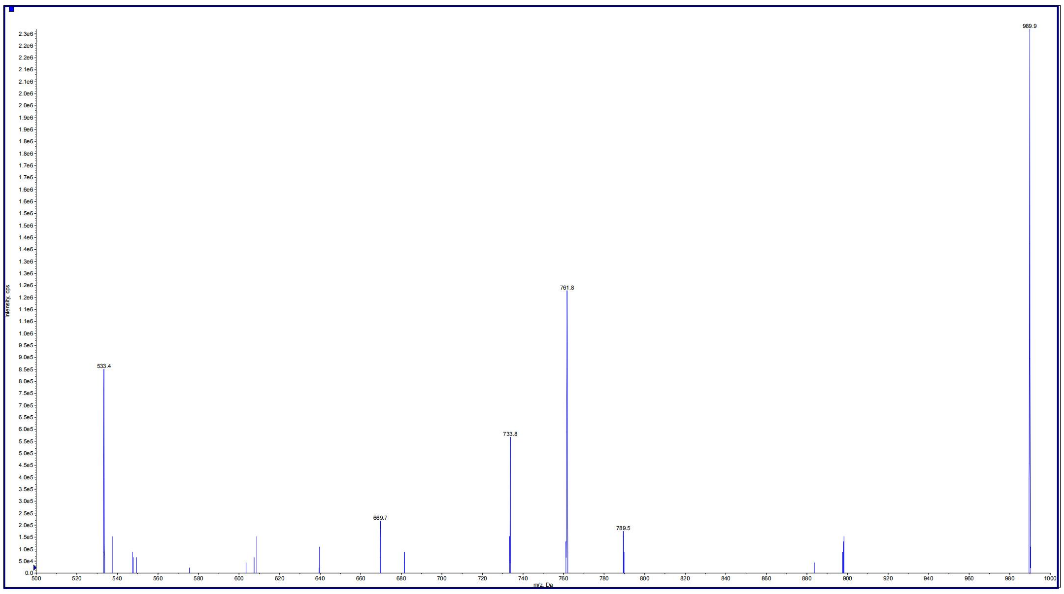


Zeaxanthin-dipalmitate_

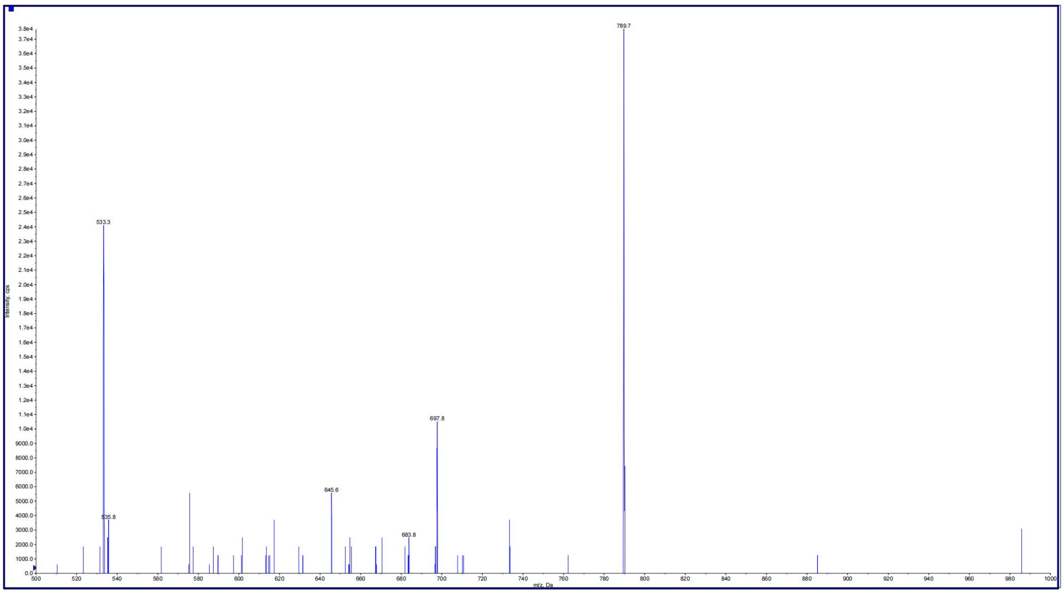

Antheraxanthin

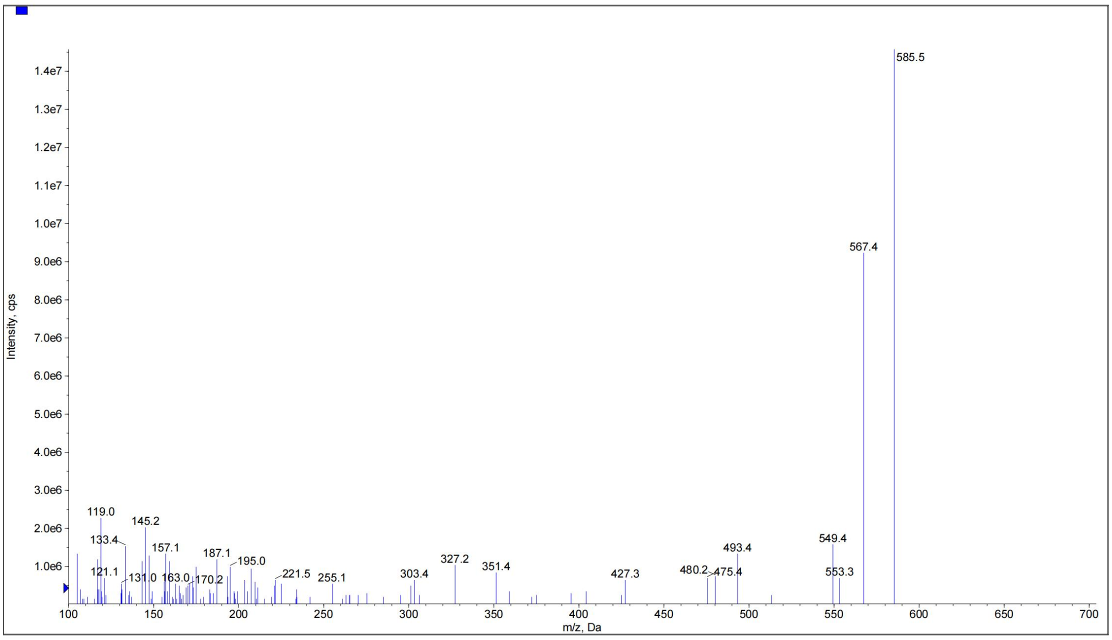


Zeaxanthin

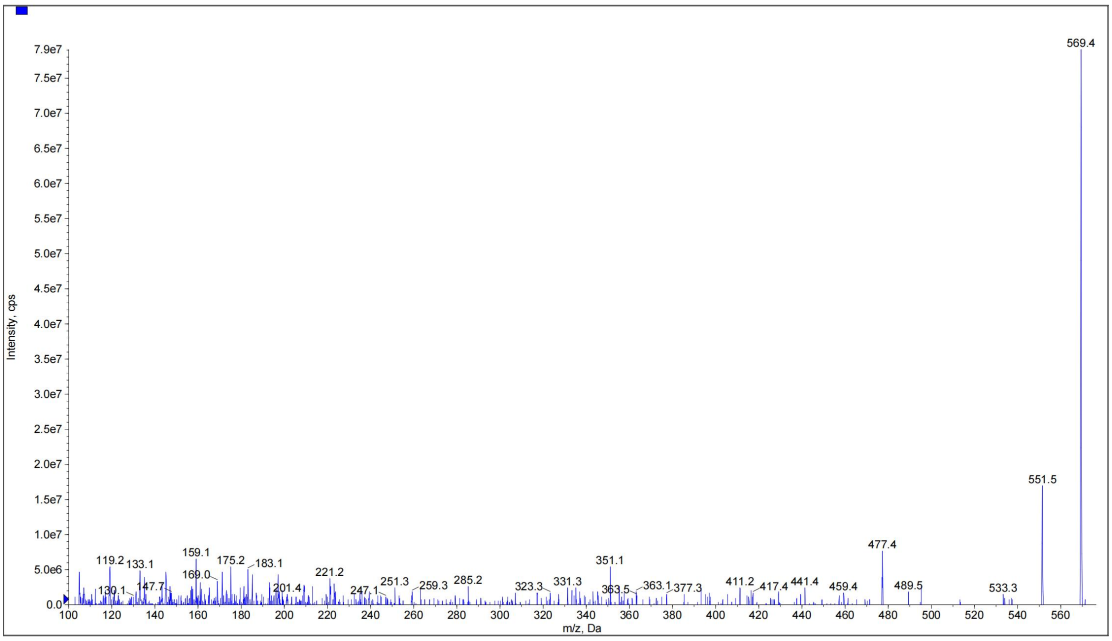

Violaxanthin

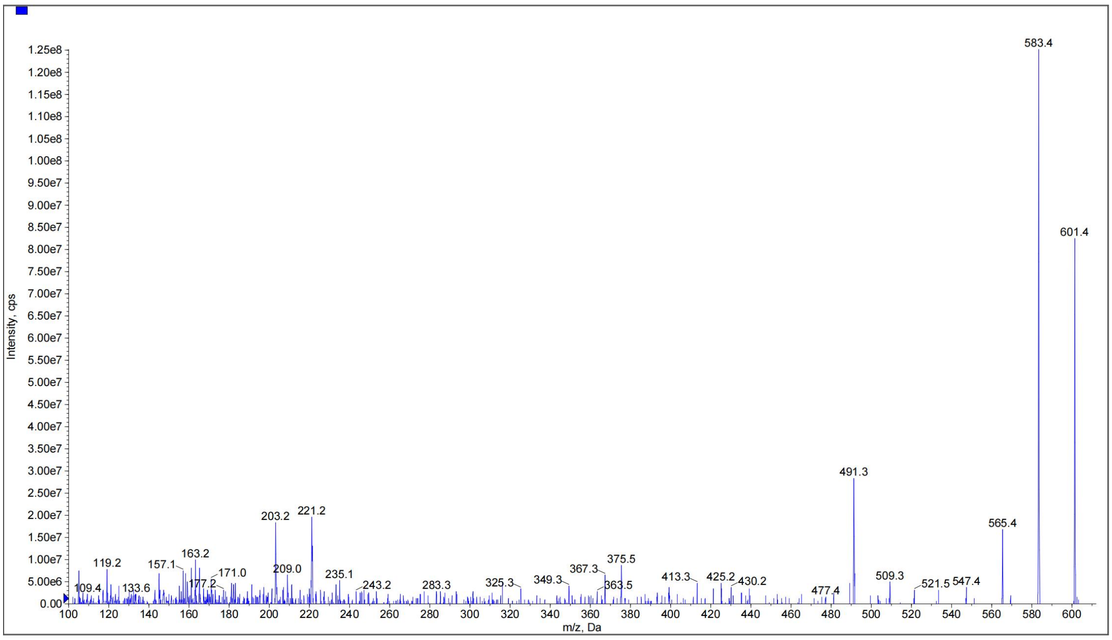


Neoxanthin

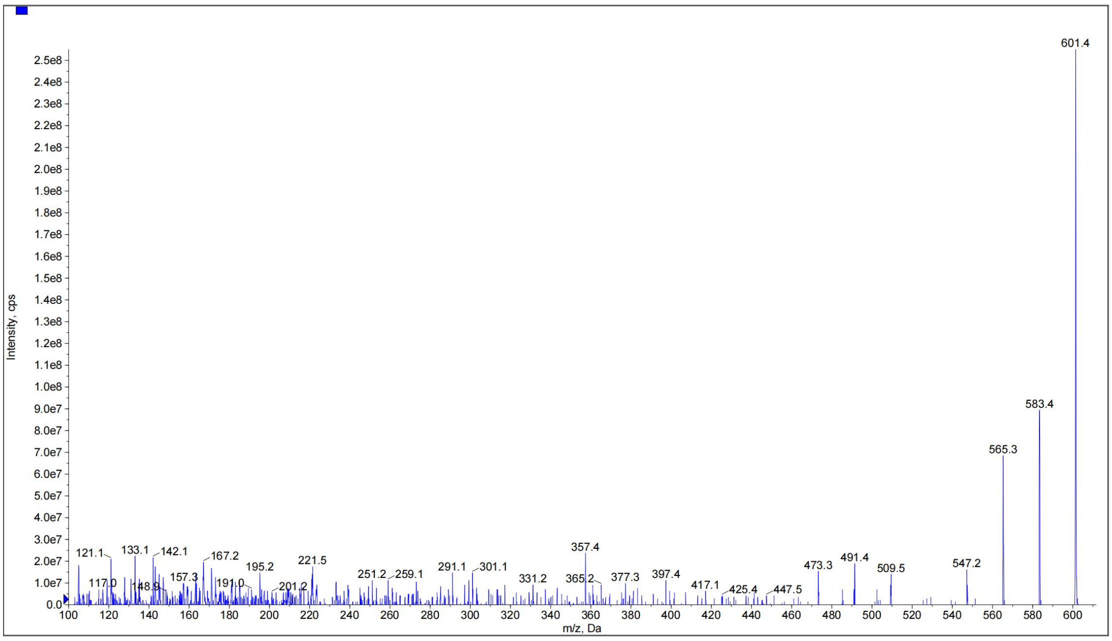

Lutein

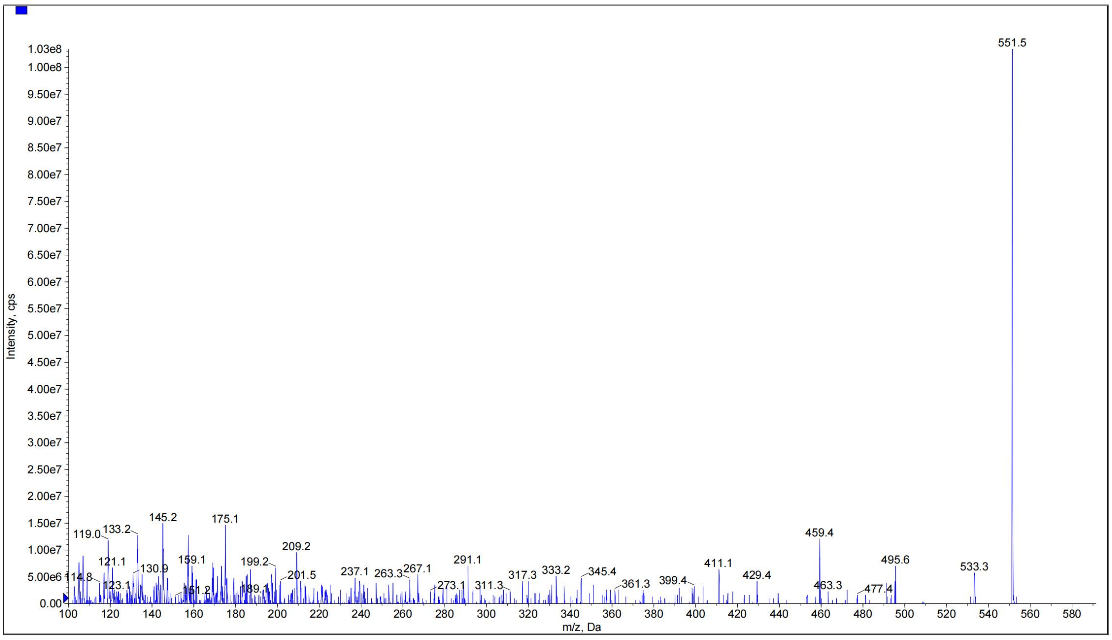


Canthaxanthin

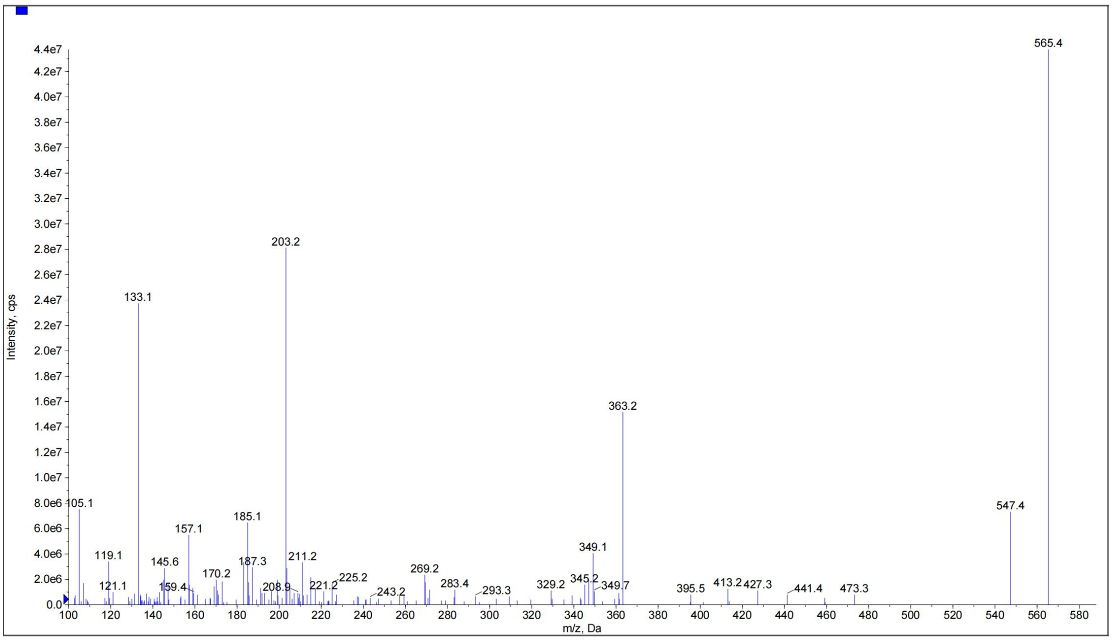

Echinenone

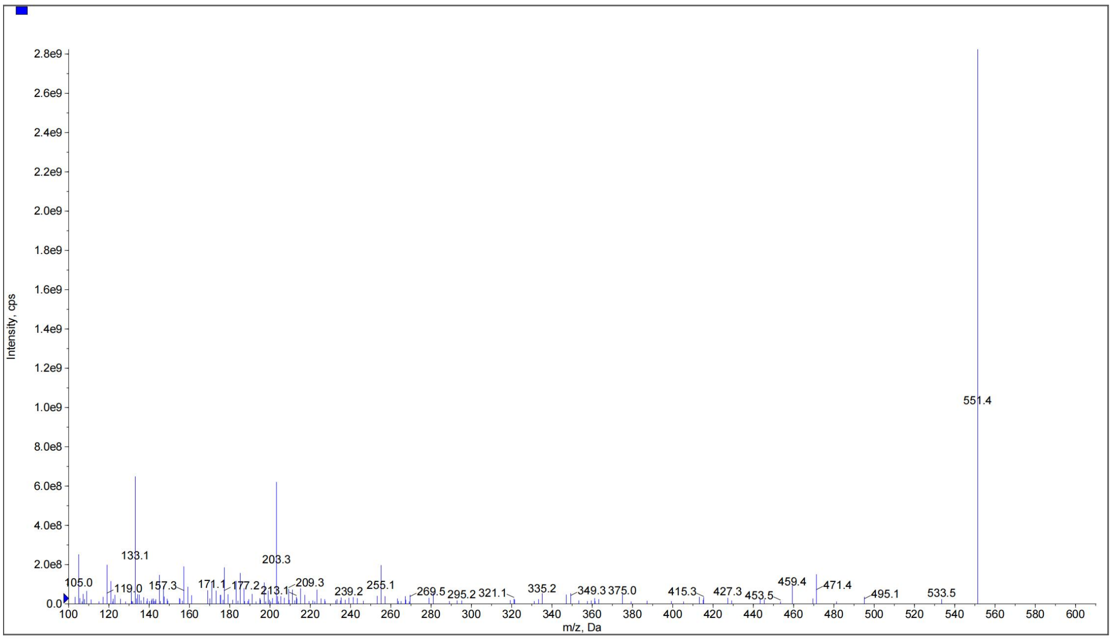

Intensity, cps
m/z, Da
551.4
133.1
203.3
105.0
119.0
157.3
171.1
177.2
213.1
209.3
239.2
255.1
269.5
295.2
321.1
335.2
349.3
375.0
415.3
427.3
453.5
459.4
471.4
495.1
533.5
