# Supplementary material for: Comparative Analysis of Individual Carotenoid Profiles in Yellow- and White-Fleshed Potatoes (Solanum tuberosum L.) During Tuber Development
Source: Foods. 2024 Nov 20;13(22):3691. doi: 10.3390/foods13223691 (PMC11593604; doi:10.3390/foods13223691)
Supplement: Supplementary file 1 [file foods-13-03691-s001.zip › Supplementary Figure S2.pdf]

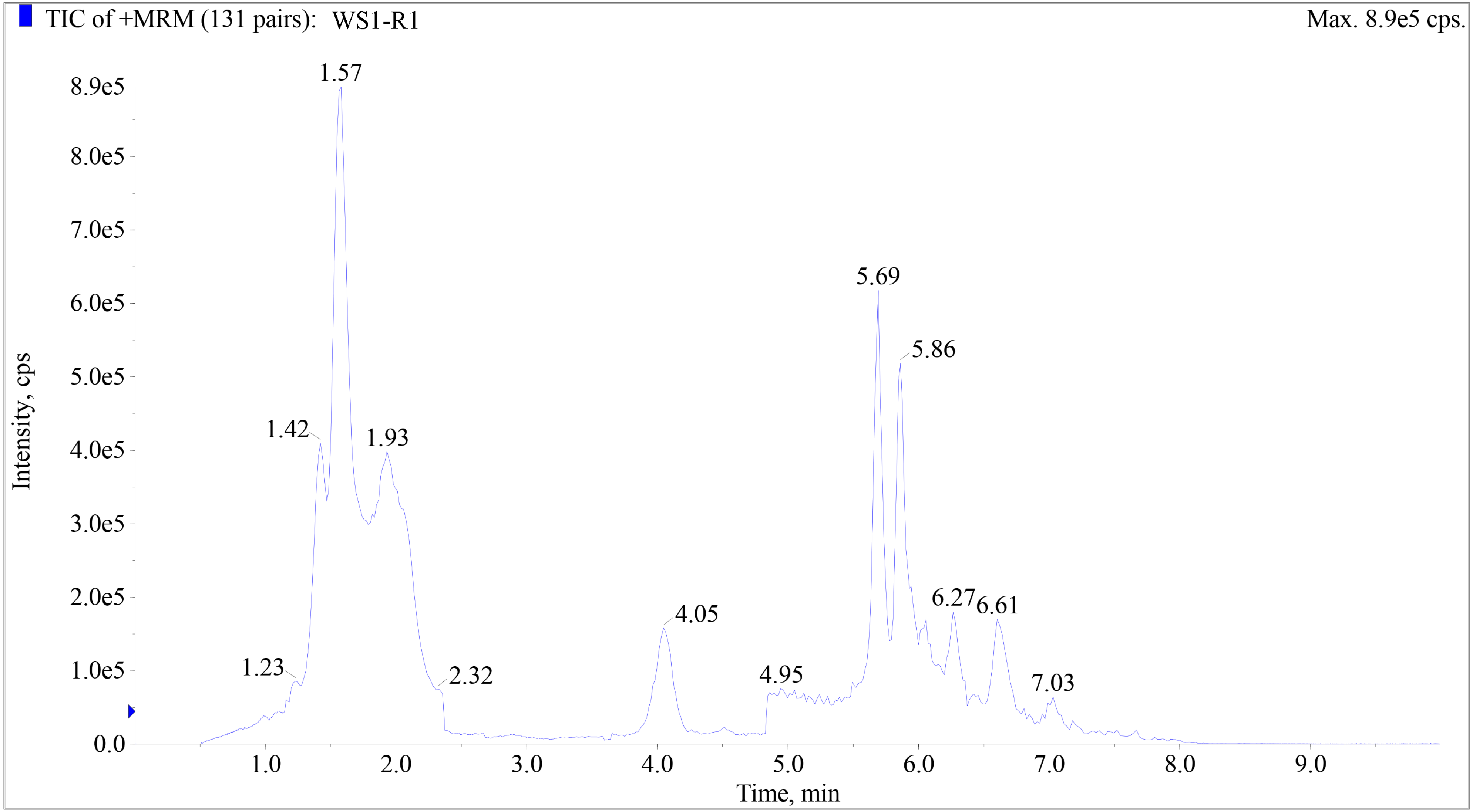
TIC of +MRM (131 pairs): WS1-R1
Max. 8.9e5 cps.
Intensity, cps
Time, min
8.9e5
8.0e5
7.0e5
6.0e5
5.0e5
4.0e5
3.0e5
2.0e5
1.0e5
0.0
1.0
2.0
3.0
4.0
5.0
6.0
7.0
8.0
9.0
1.57
1.42
1.93
1.23
2.32
4.05
4.95
5.69
5.86
6.27
6.61
7.03

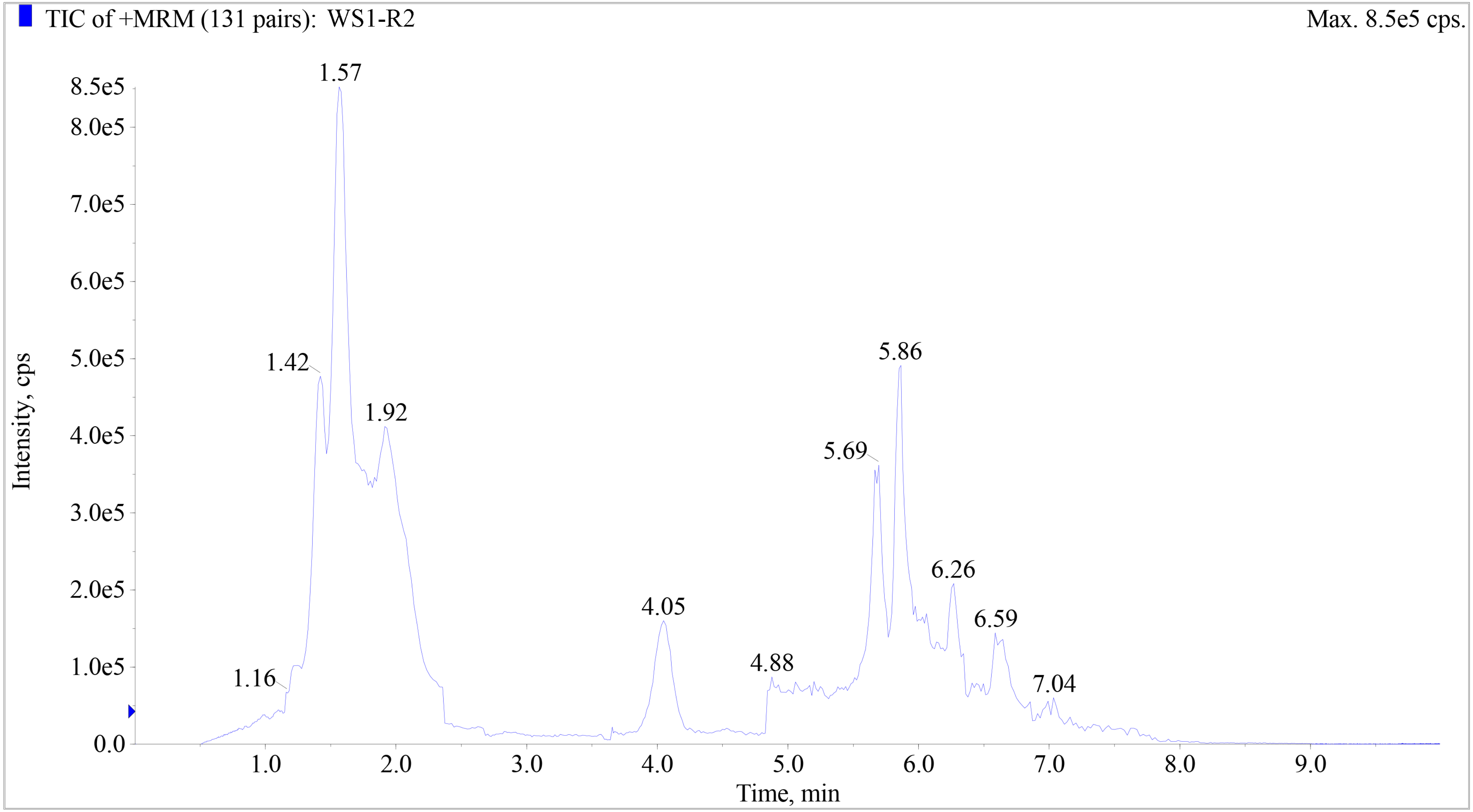

TIC of +MRM (131 pairs): WS1-R2
Max. 8.5e5 cps.
Intensity, cps
8.5e5
8.0e5
7.0e5
6.0e5
5.0e5
4.0e5
3.0e5
2.0e5
1.0e5
0.0
1.57
1.42
1.92
1.16
4.05
4.88
5.69
5.86
6.26
6.59
7.04
1.0
2.0
3.0
4.0
5.0
6.0
7.0
8.0
9.0
Time, min

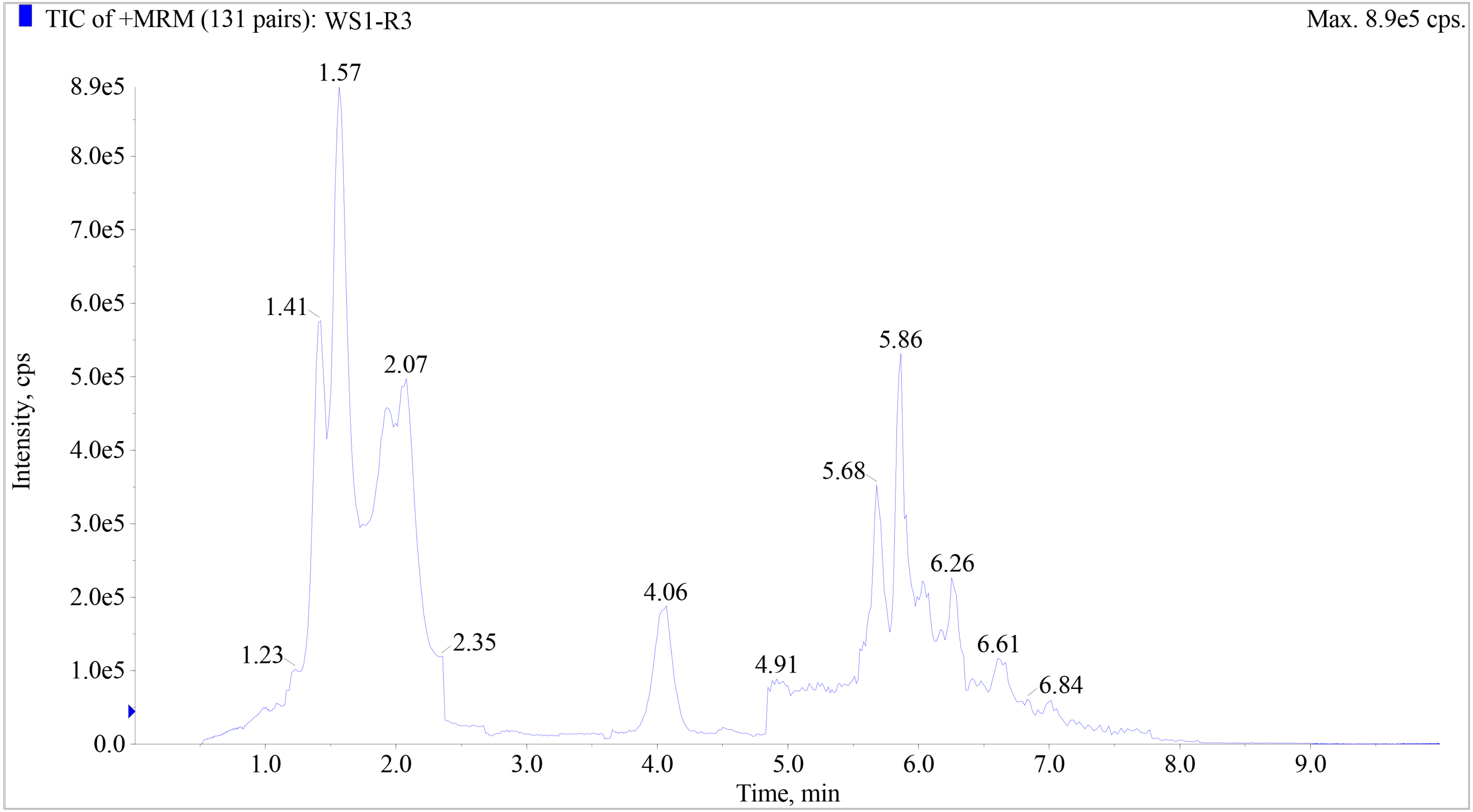

TIC of +MRM (131 pairs): WS1-R3
Max. 8.9e5 cps.
Intensity, cps
8.9e5
8.0e5
7.0e5
6.0e5
5.0e5
4.0e5
3.0e5
2.0e5
1.0e5
0.0
1.57
1.41
2.07
1.23
2.35
4.06
4.91
5.68
5.86
6.26
6.61
6.84
1.0
2.0
3.0
4.0
5.0
6.0
7.0
8.0
9.0
Time, min

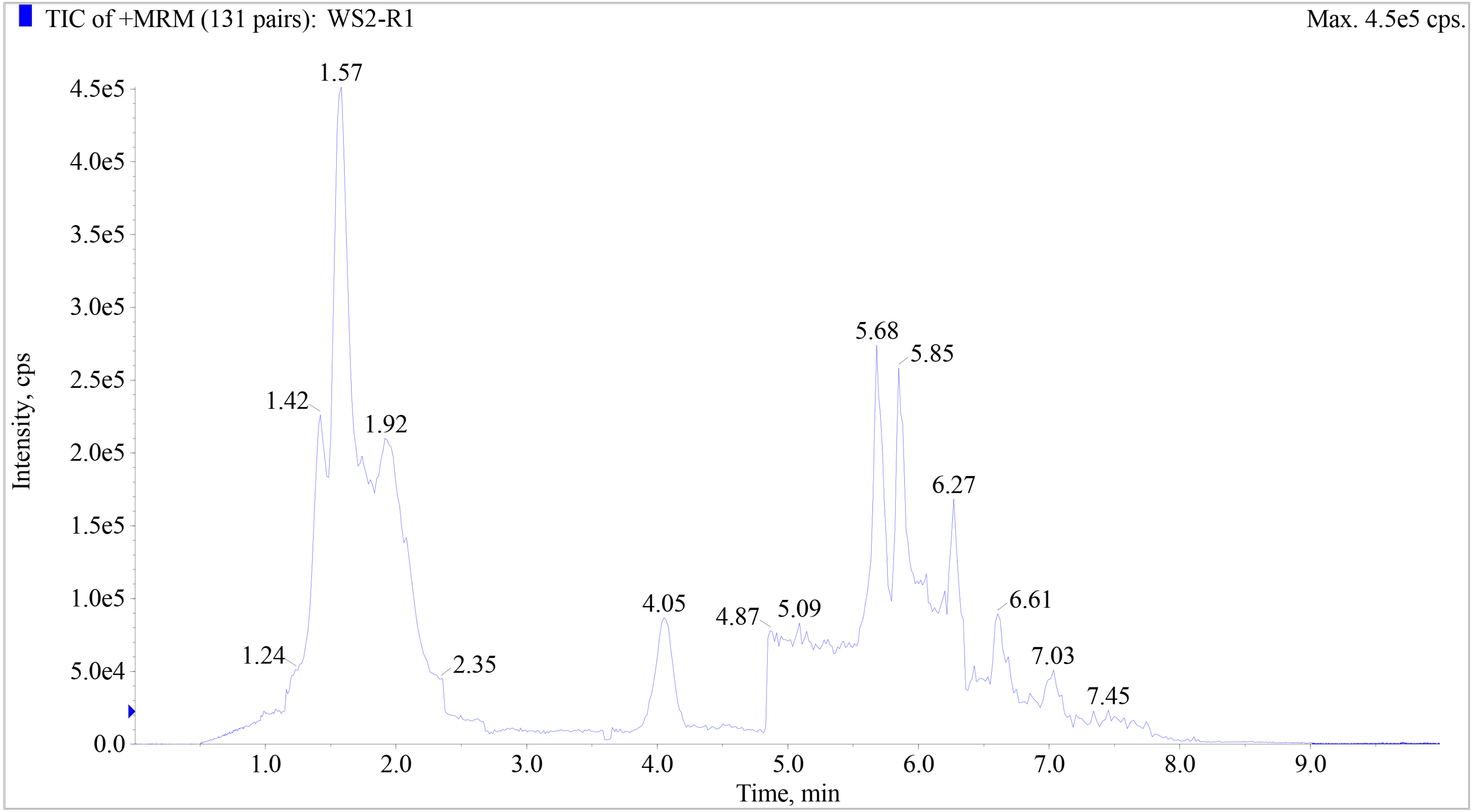

TIC of +MRM (131 pairs): WS2-R1
Max. 4.5e5 cps.
Intensity, cps
Time, min
1.57
1.42
1.92
1.24
2.35
4.05
4.87
5.09
5.68
5.85
6.27
6.61
7.03
7.45

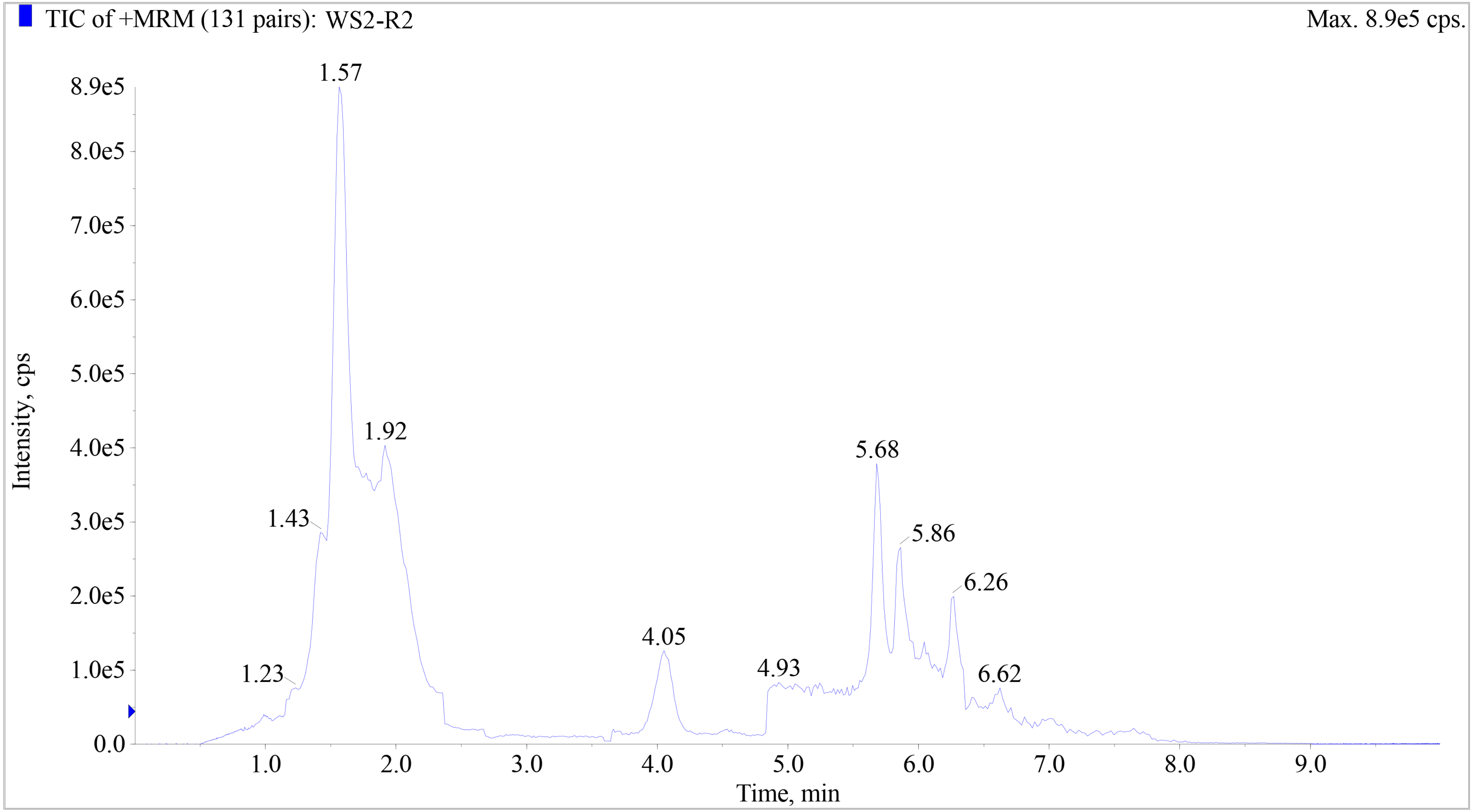
TIC of +MRM (131 pairs): WS2-R2
Max. 8.9e5 cps.
Intensity, cps
Time, min
1.57
1.92
1.43
1.23
4.05
4.93
5.68
5.86
6.26
6.62
8.9e5
8.0e5
7.0e5
6.0e5
5.0e5
4.0e5
3.0e5
2.0e5
1.0e5
0.0
1.0
2.0
3.0
4.0
5.0
6.0
7.0
8.0
9.0

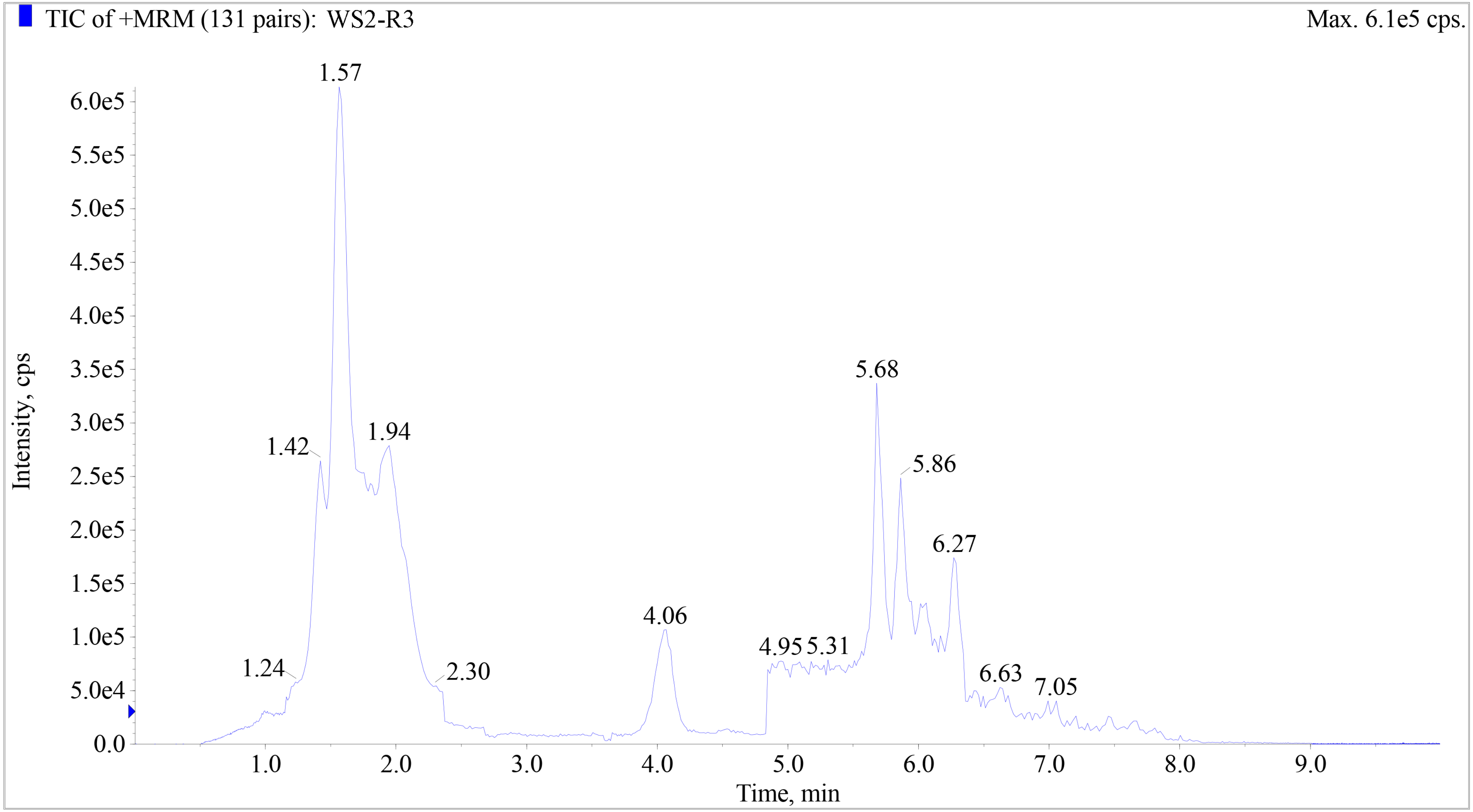

TIC of +MRM (131 pairs): WS2-R3
Max. 6.1e5 cps.
Intensity, cps
Time, min
1.57
1.42
1.94
1.24
2.30
4.06
4.95
5.31
5.68
5.86
6.27
6.63
7.05

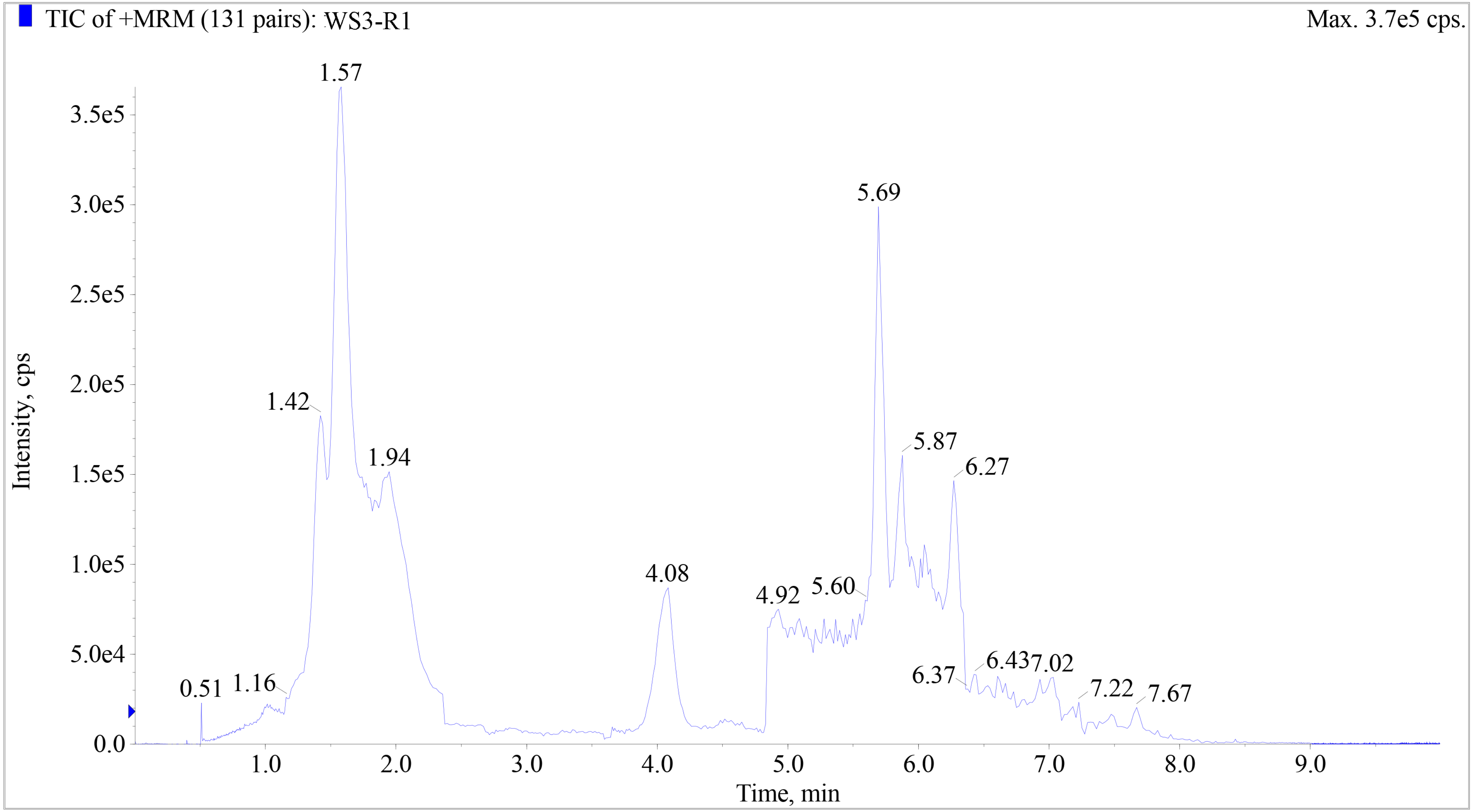

TIC of +MRM (131 pairs): WS3-R1
Max. 3.7e5 cps.
Intensity, cps
Time, min
3.5e5
3.0e5
2.5e5
2.0e5
1.5e5
1.0e5
5.0e4
0.0
1.0
2.0
3.0
4.0
5.0
6.0
7.0
8.0
9.0
1.57
1.42
1.94
0.51
1.16
4.08
4.92
5.60
5.69
5.87
6.27
6.37
6.43
7.02
7.22
7.67

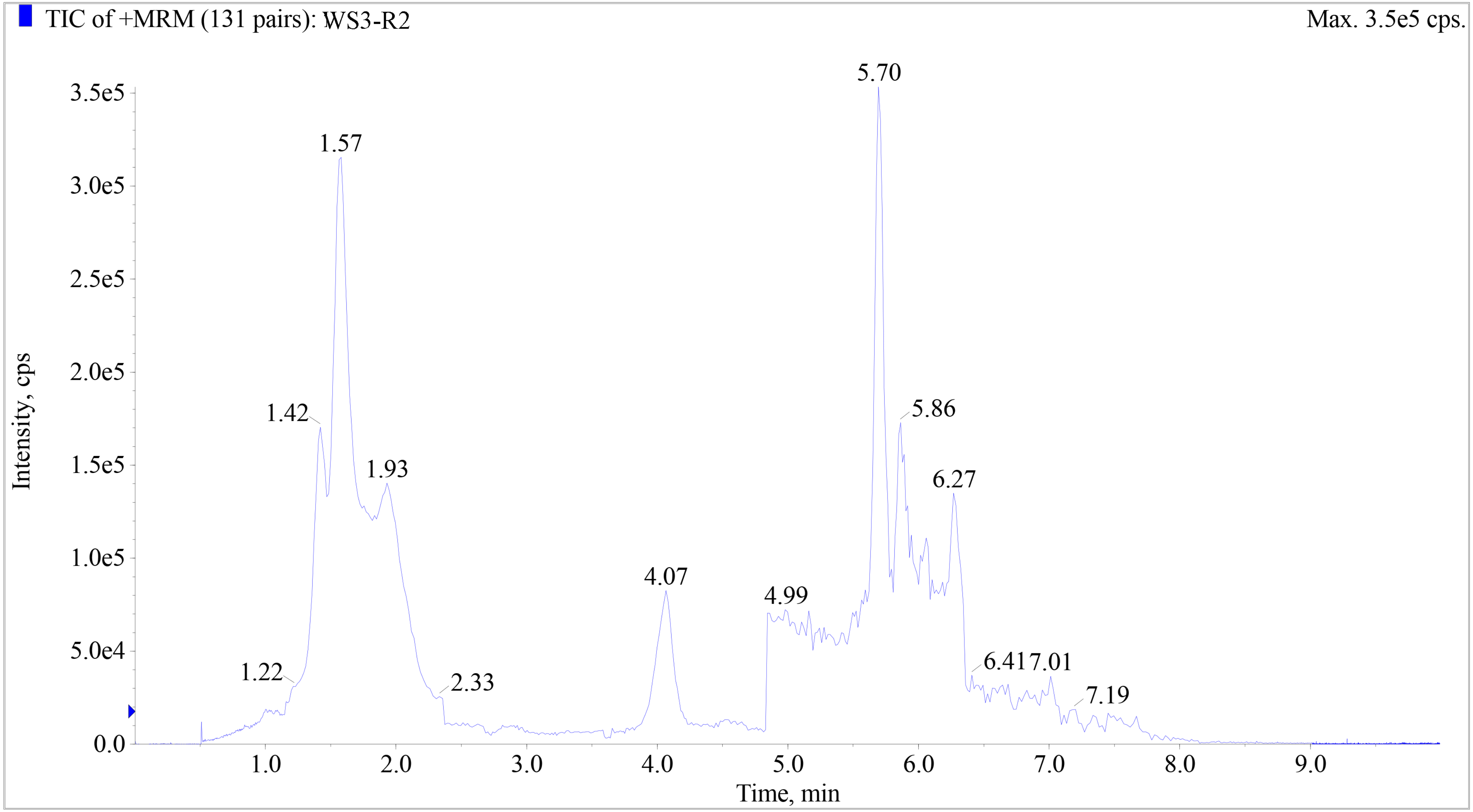

TIC of +MRM (131 pairs): WS3-R2
Max. 3.5e5 cps.
Intensity, cps
Time, min
3.5e5
3.0e5
2.5e5
2.0e5
1.5e5
1.0e5
5.0e4
0.0
1.0
2.0
3.0
4.0
5.0
6.0
7.0
8.0
9.0
1.22
1.42
1.57
1.93
2.33
4.07
4.99
5.70
5.86
6.27
6.41
7.01
7.19

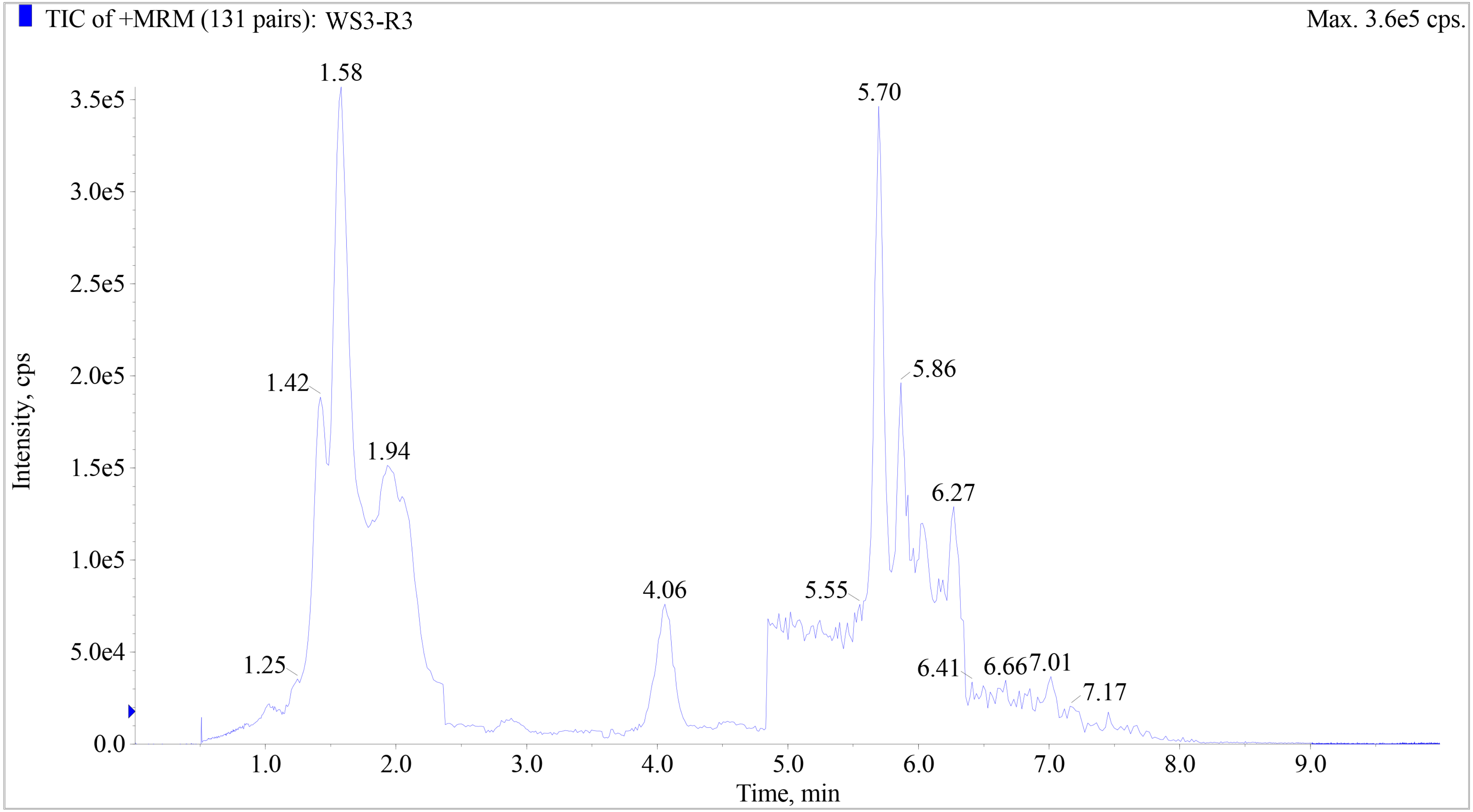

TIC of +MRM (131 pairs): WS3-R3
Max. 3.6e5 cps.
1.58
5.70
1.42
5.86
1.94
6.27
4.06
5.55
1.25
6.41
6.66
7.01
7.17
3.5e5
3.0e5
2.5e5
2.0e5
1.5e5
1.0e5
5.0e4
0.0
Intensity, cps
1.0
2.0
3.0
4.0
5.0
6.0
7.0
8.0
9.0
Time, min

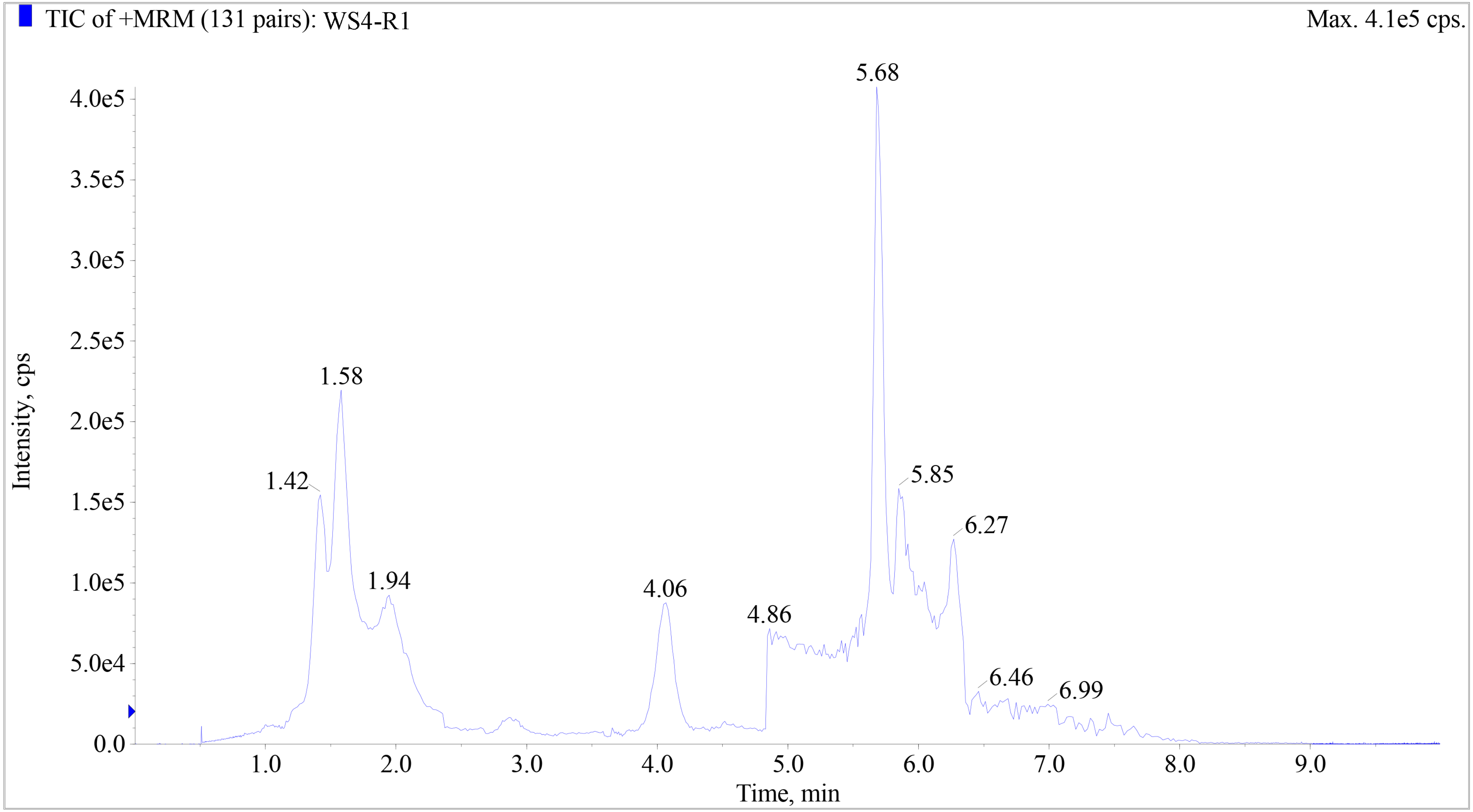

TIC of +MRM (131 pairs): WS4-R1
Max. 4.1e5 cps.
Intensity, cps
Time, min
4.0e5
3.5e5
3.0e5
2.5e5
2.0e5
1.5e5
1.0e5
5.0e4
0.0
1.0
2.0
3.0
4.0
5.0
6.0
7.0
8.0
9.0
5.68
1.58
1.42
1.94
4.06
4.86
5.85
6.27
6.46
6.99

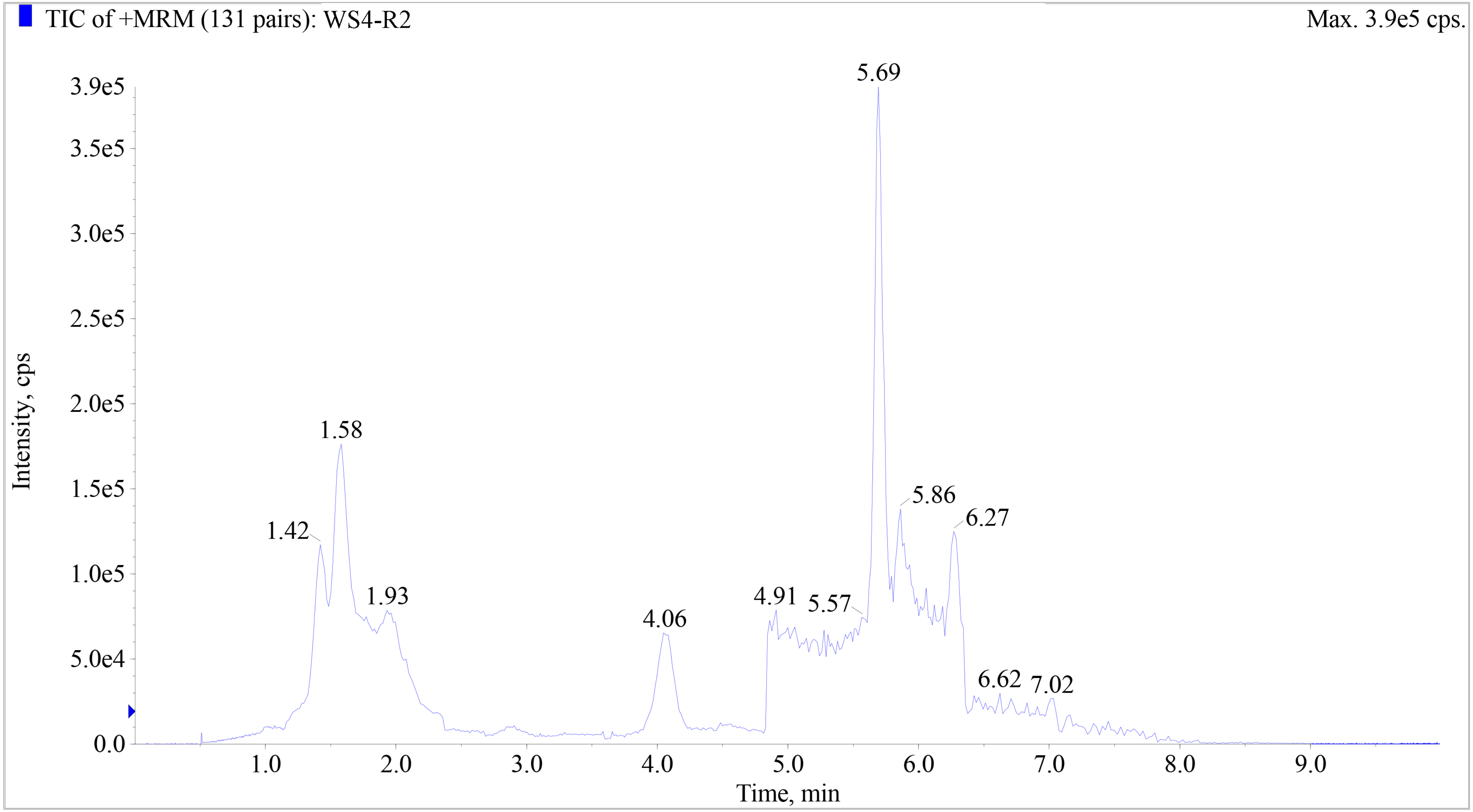

TIC of +MRM (131 pairs): WS4-R2
Max. 3.9e5 cps.
Intensity, cps
Time, min
3.9e5
3.5e5
3.0e5
2.5e5
2.0e5
1.5e5
1.0e5
5.0e4
0.0
1.0
2.0
3.0
4.0
5.0
6.0
7.0
8.0
9.0
5.69
1.58
1.42
1.93
4.06
4.91
5.57
5.86
6.27
6.62
7.02

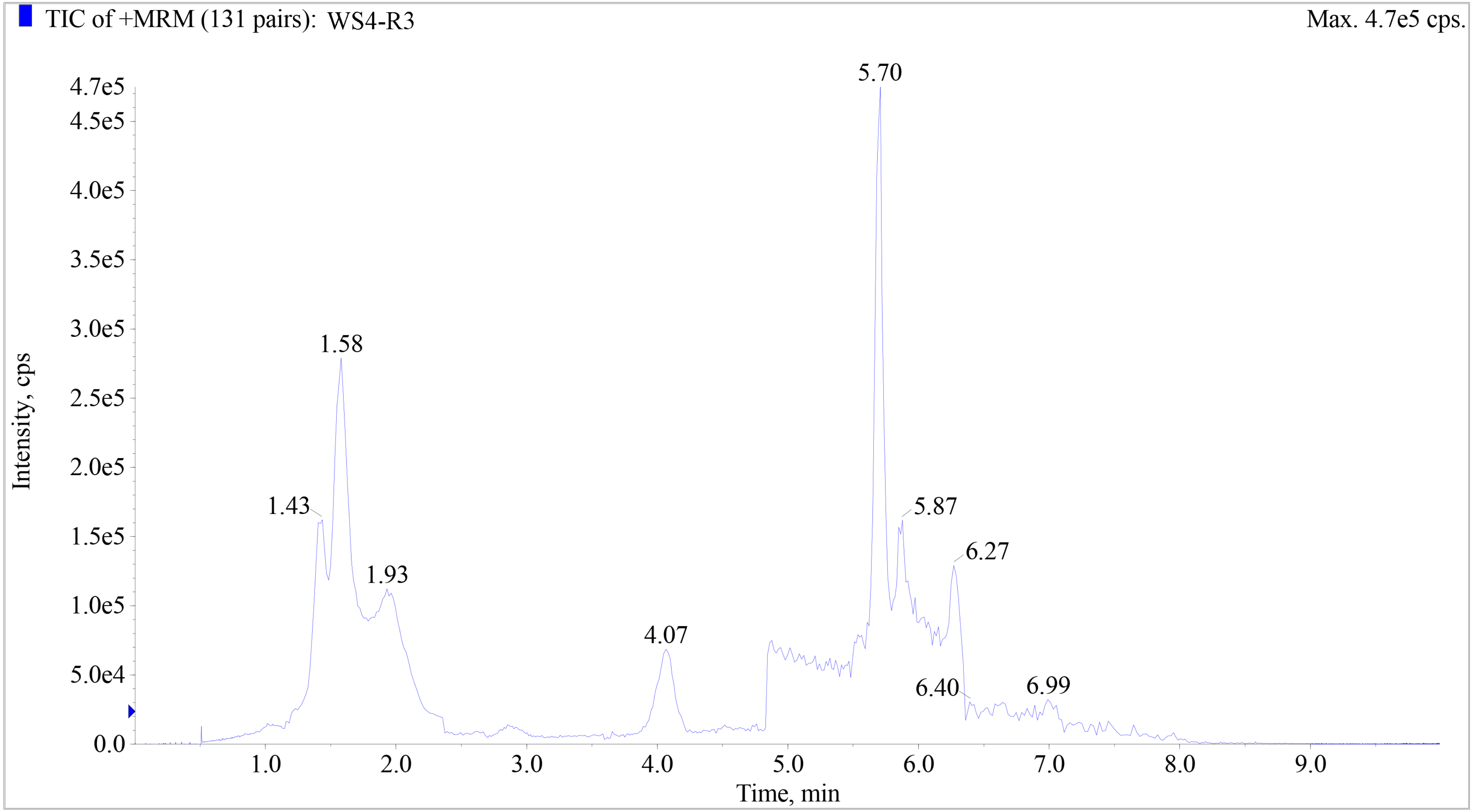
TIC of +MRM (131 pairs): WS4-R3
Max. 4.7e5 cps.
4.7e5
4.5e5
4.0e5
3.5e5
3.0e5
2.5e5
2.0e5
1.5e5
1.0e5
5.0e4
0.0
Intensity, cps
1.0
2.0
3.0
4.0
5.0
6.0
7.0
8.0
9.0
Time, min
1.43
1.58
1.93
4.07
5.70
5.87
6.27
6.40
6.99

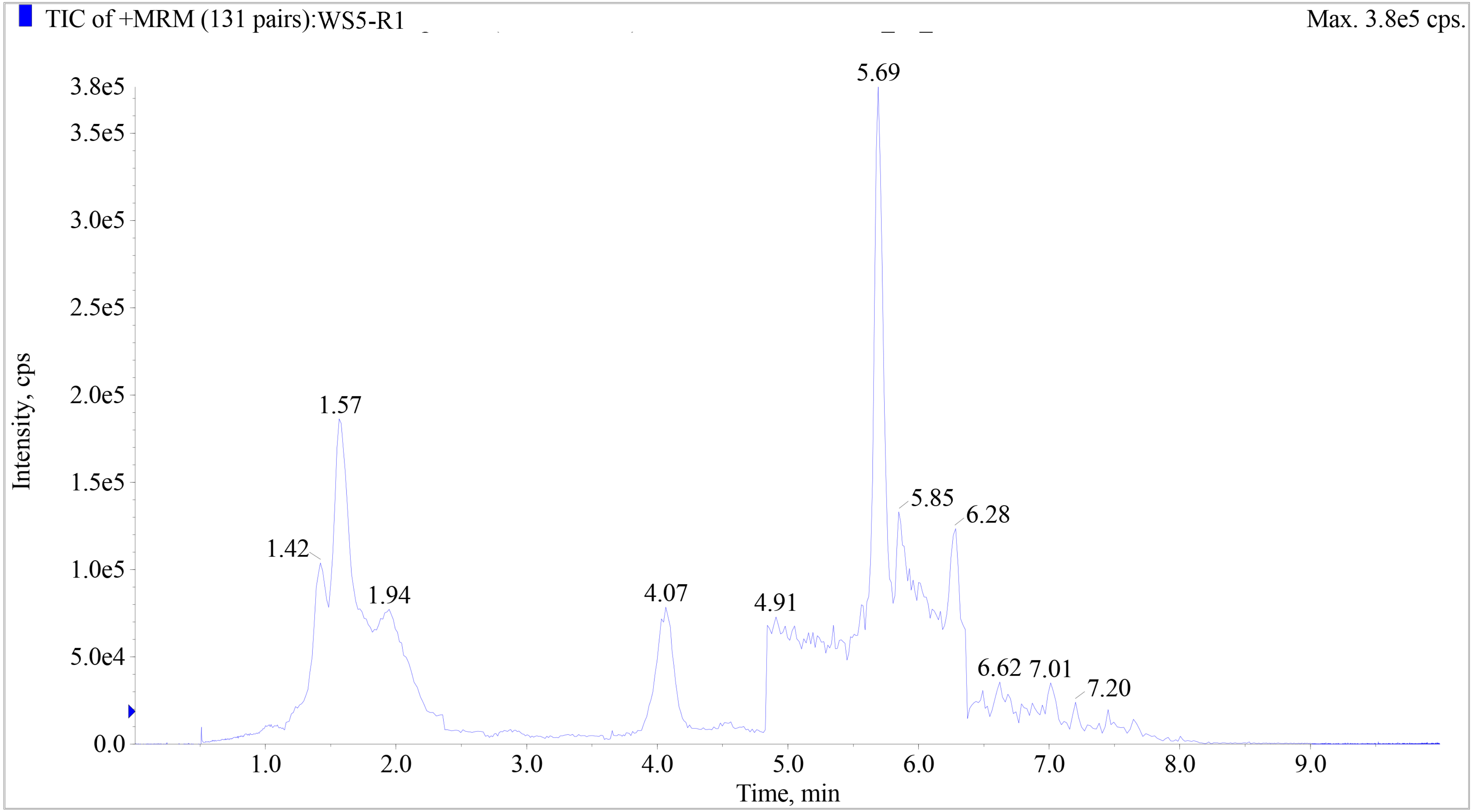
TIC of +MRM (131 pairs):WS5-R1
Max. 3.8e5 cps.
Intensity, cps
Time, min
3.8e5
3.5e5
3.0e5
2.5e5
2.0e5
1.5e5
1.0e5
5.0e4
0.0
1.0
2.0
3.0
4.0
5.0
6.0
7.0
8.0
9.0
5.69
1.57
1.42
1.94
4.07
4.91
5.85
6.28
6.62
7.01
7.20

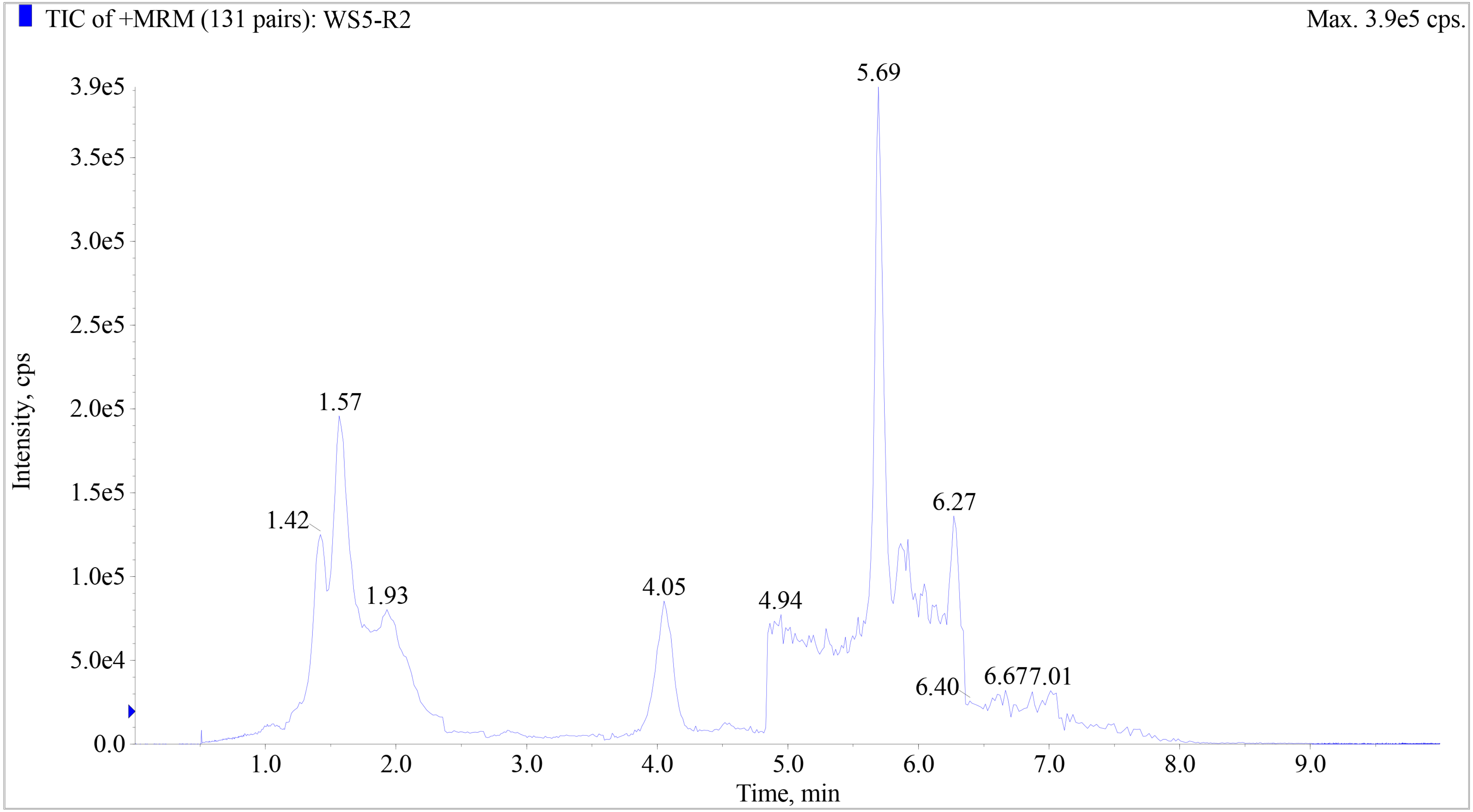
TIC of +MRM (131 pairs): WS5-R2
Max. 3.9e5 cps.
Intensity, cps
Time, min
3.9e5
3.5e5
3.0e5
2.5e5
2.0e5
1.5e5
1.0e5
5.0e4
0.0
1.0
2.0
3.0
4.0
5.0
6.0
7.0
8.0
9.0
1.42
1.57
1.93
4.05
4.94
5.69
6.27
6.40
6.67
7.01

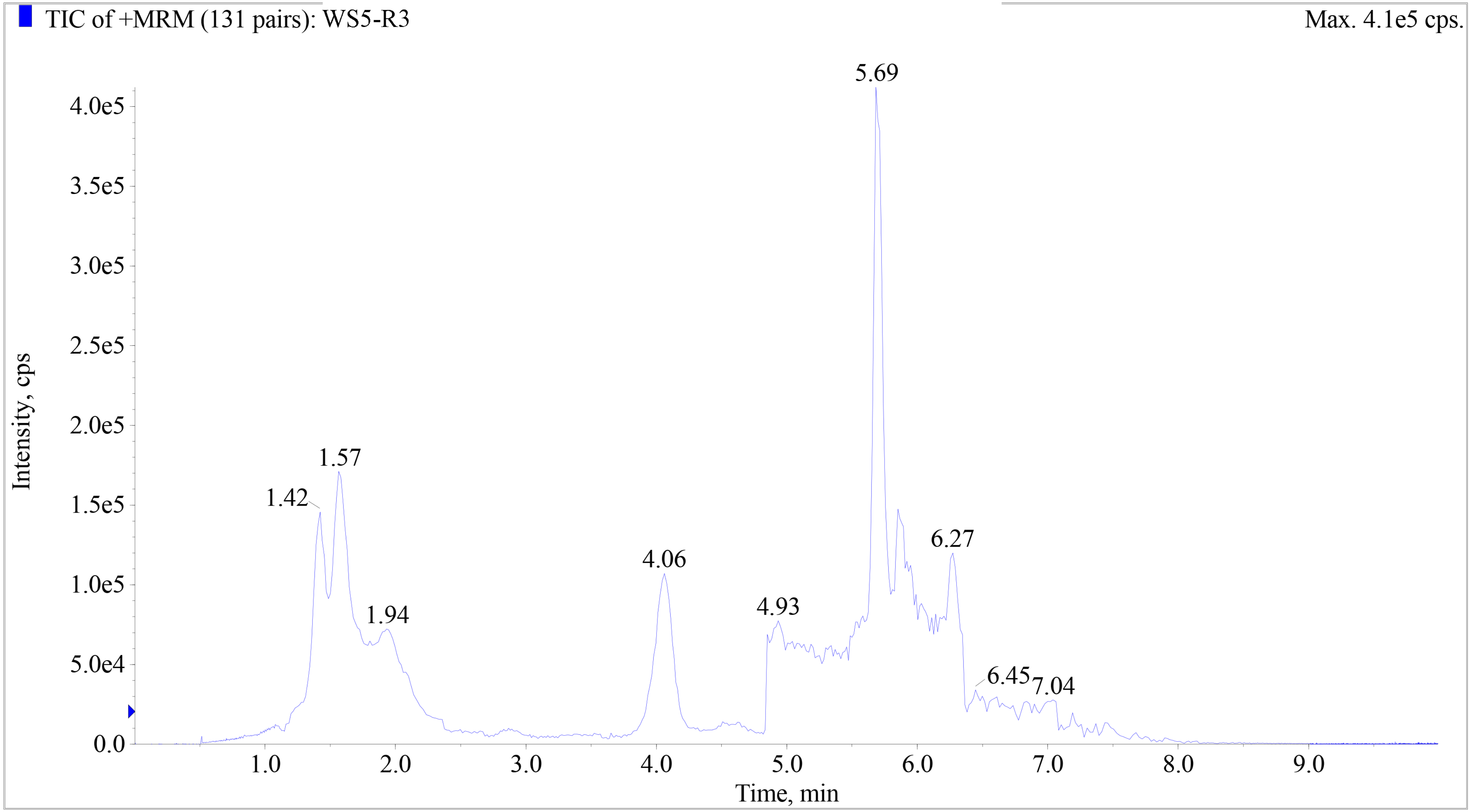

TIC of +MRM (131 pairs): WS5-R3
Max. 4.1e5 cps.
Intensity, cps
4.0e5
3.5e5
3.0e5
2.5e5
2.0e5
1.5e5
1.0e5
5.0e4
0.0
1.42
1.57
1.94
4.06
4.93
5.69
6.27
6.45
7.04
1.0
2.0
3.0
4.0
5.0
6.0
7.0
8.0
9.0
Time, min

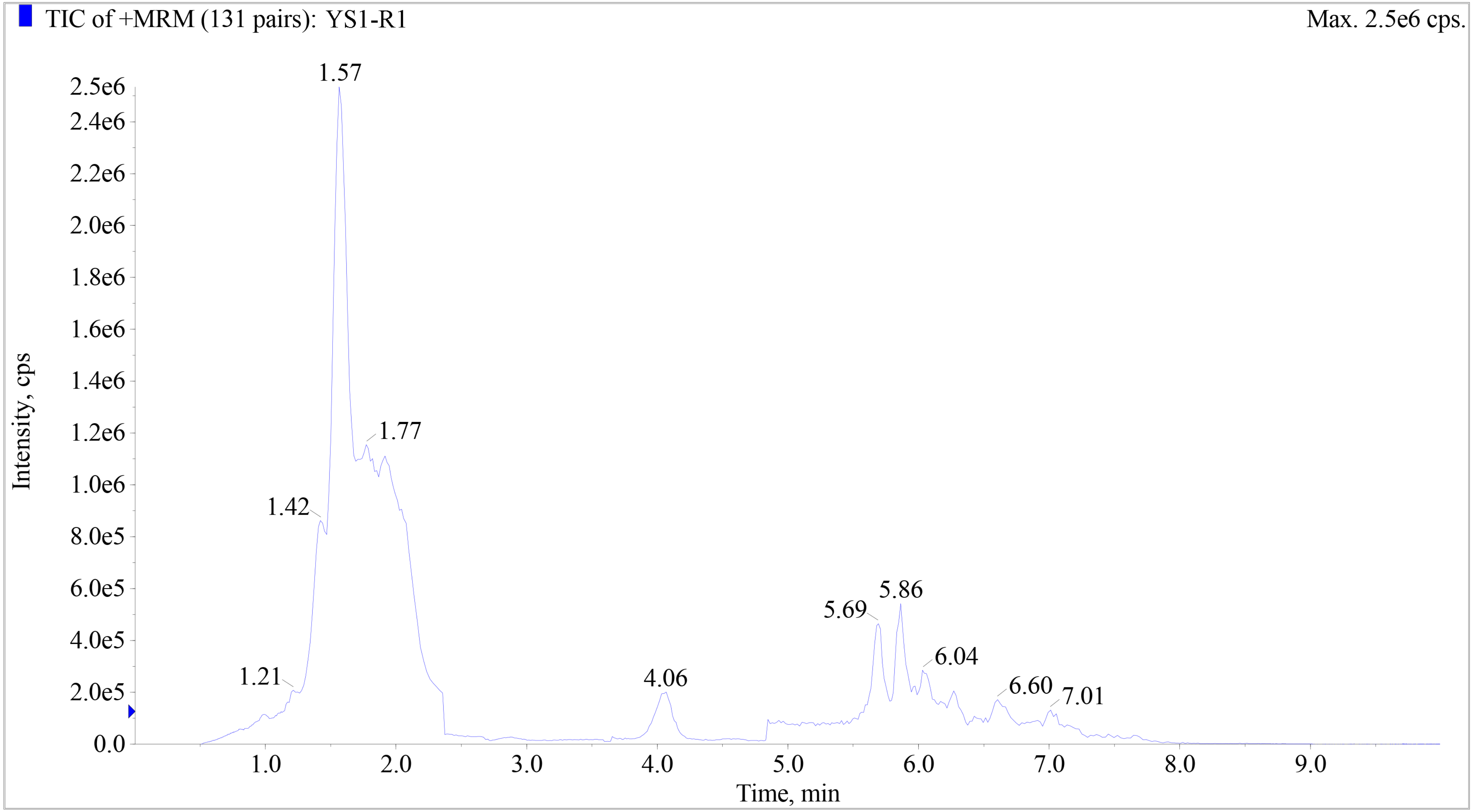

TIC of +MRM (131 pairs): YS1-R1
Max. 2.5e6 cps.
Intensity, cps
2.5e6
2.4e6
2.2e6
2.0e6
1.8e6
1.6e6
1.4e6
1.2e6
1.0e6
8.0e5
6.0e5
4.0e5
2.0e5
0.0
1.57
1.77
1.42
1.21
4.06
5.69
5.86
6.04
6.60
7.01
1.0
2.0
3.0
4.0
5.0
6.0
7.0
8.0
9.0
Time, min

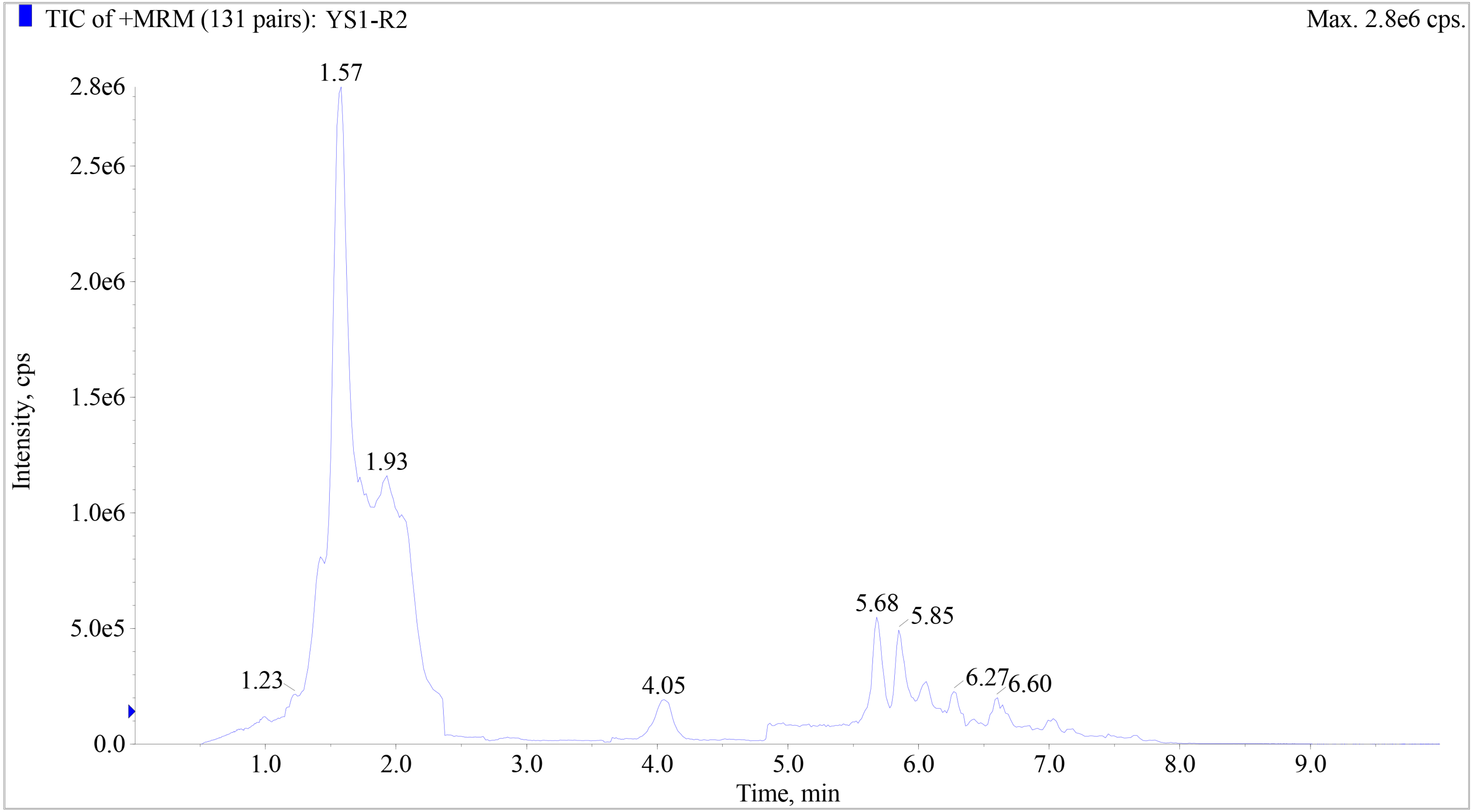
TIC of +MRM (131 pairs): YS1-R2
Max. 2.8e6 cps.
1.57
1.93
1.23
4.05
5.68
5.85
6.27
6.60
2.8e6
2.5e6
2.0e6
1.5e6
1.0e6
5.0e5
0.0
Intensity, cps
1.0
2.0
3.0
4.0
5.0
6.0
7.0
8.0
9.0
Time, min

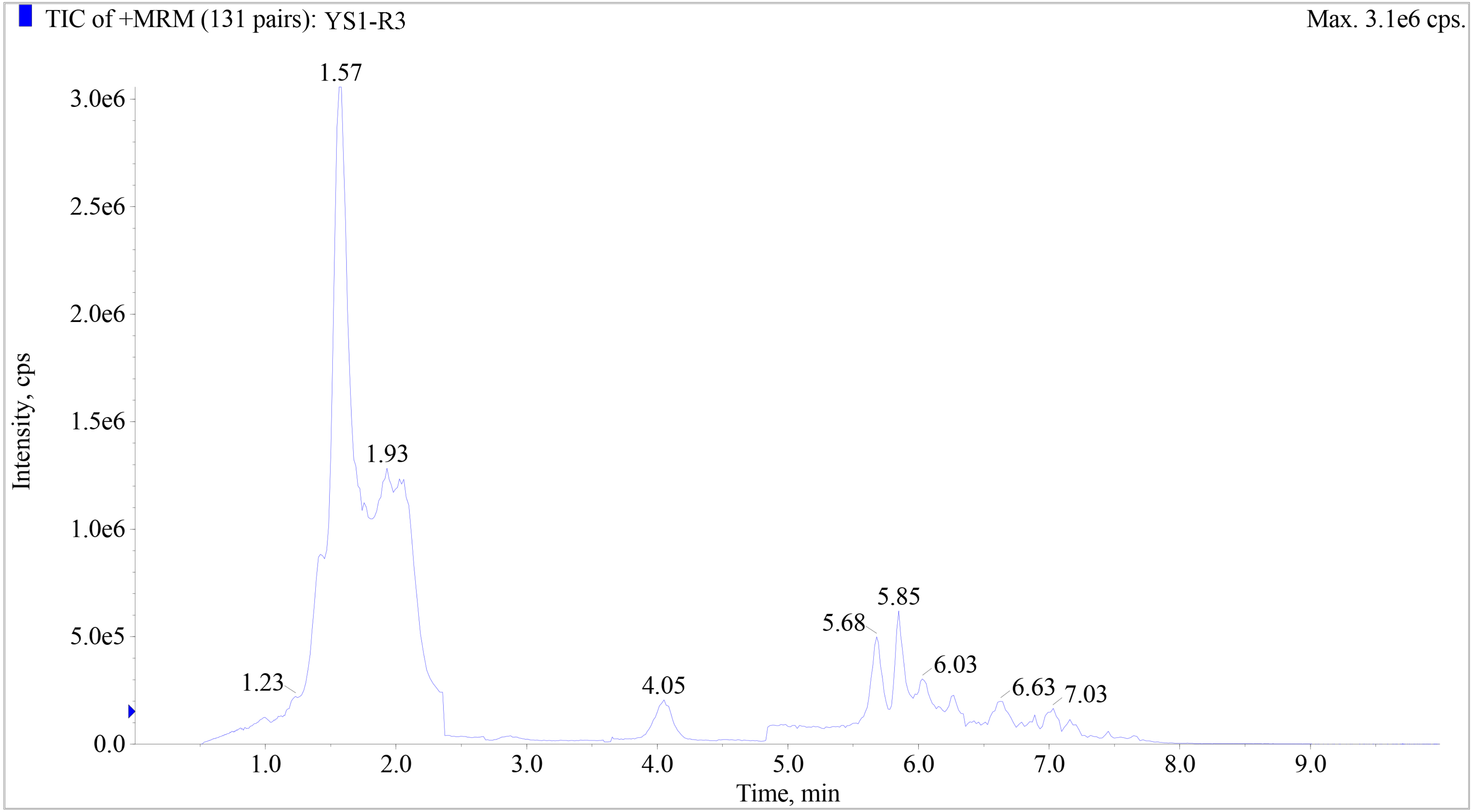

TIC of +MRM (131 pairs): YS1-R3
Max. 3.1e6 cps.
1.57
1.93
1.23
4.05
5.68
5.85
6.03
6.63
7.03
3.0e6
2.5e6
2.0e6
1.5e6
1.0e6
5.0e5
0.0
Intensity, cps
1.0
2.0
3.0
4.0
5.0
6.0
7.0
8.0
9.0
Time, min

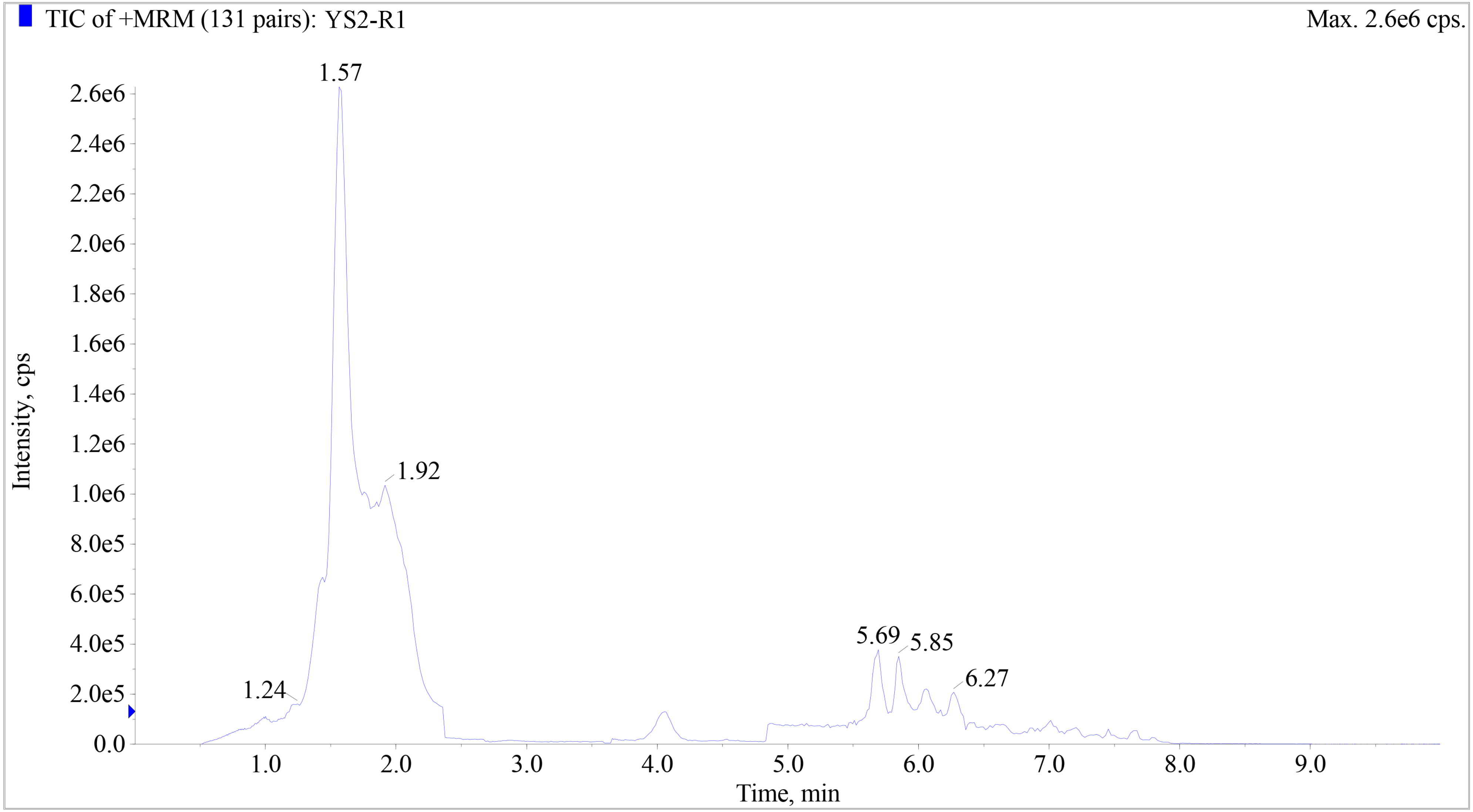

TIC of +MRM (131 pairs): YS2-R1
Max. 2.6e6 cps.
1.57
1.92
1.24
5.69
5.85
6.27
Intensity, cps
2.6e6
2.4e6
2.2e6
2.0e6
1.8e6
1.6e6
1.4e6
1.2e6
1.0e6
8.0e5
6.0e5
4.0e5
2.0e5
0.0
1.0
2.0
3.0
4.0
5.0
6.0
7.0
8.0
9.0
Time, min

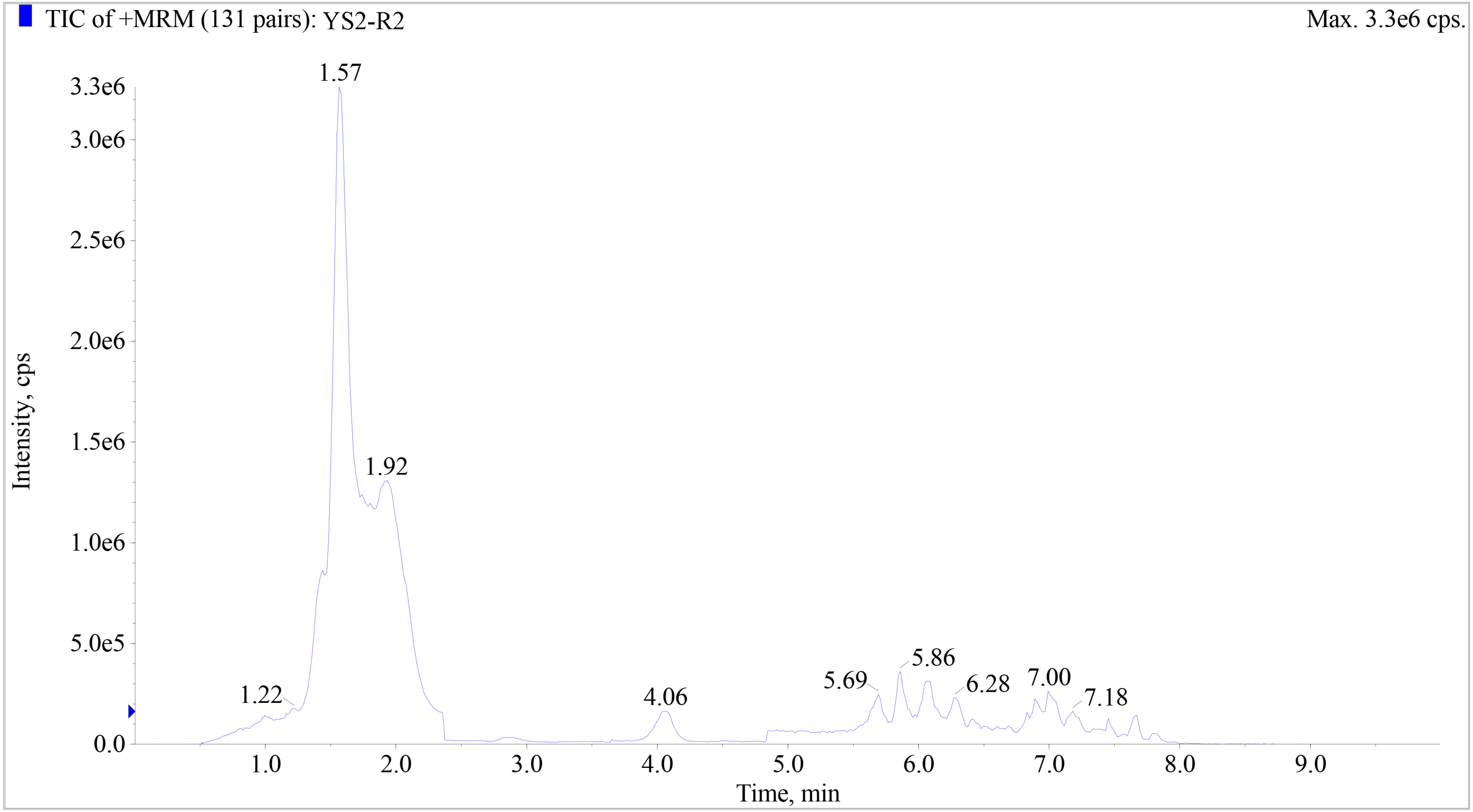

TIC of +MRM (131 pairs): YS2-R2
Max. 3.3e6 cps.
Intensity, cps
3.3e6
3.0e6
2.5e6
2.0e6
1.5e6
1.0e6
5.0e5
0.0
1.57
1.92
1.22
4.06
5.69
5.86
6.28
7.00
7.18
1.0
2.0
3.0
4.0
5.0
6.0
7.0
8.0
9.0
Time, min

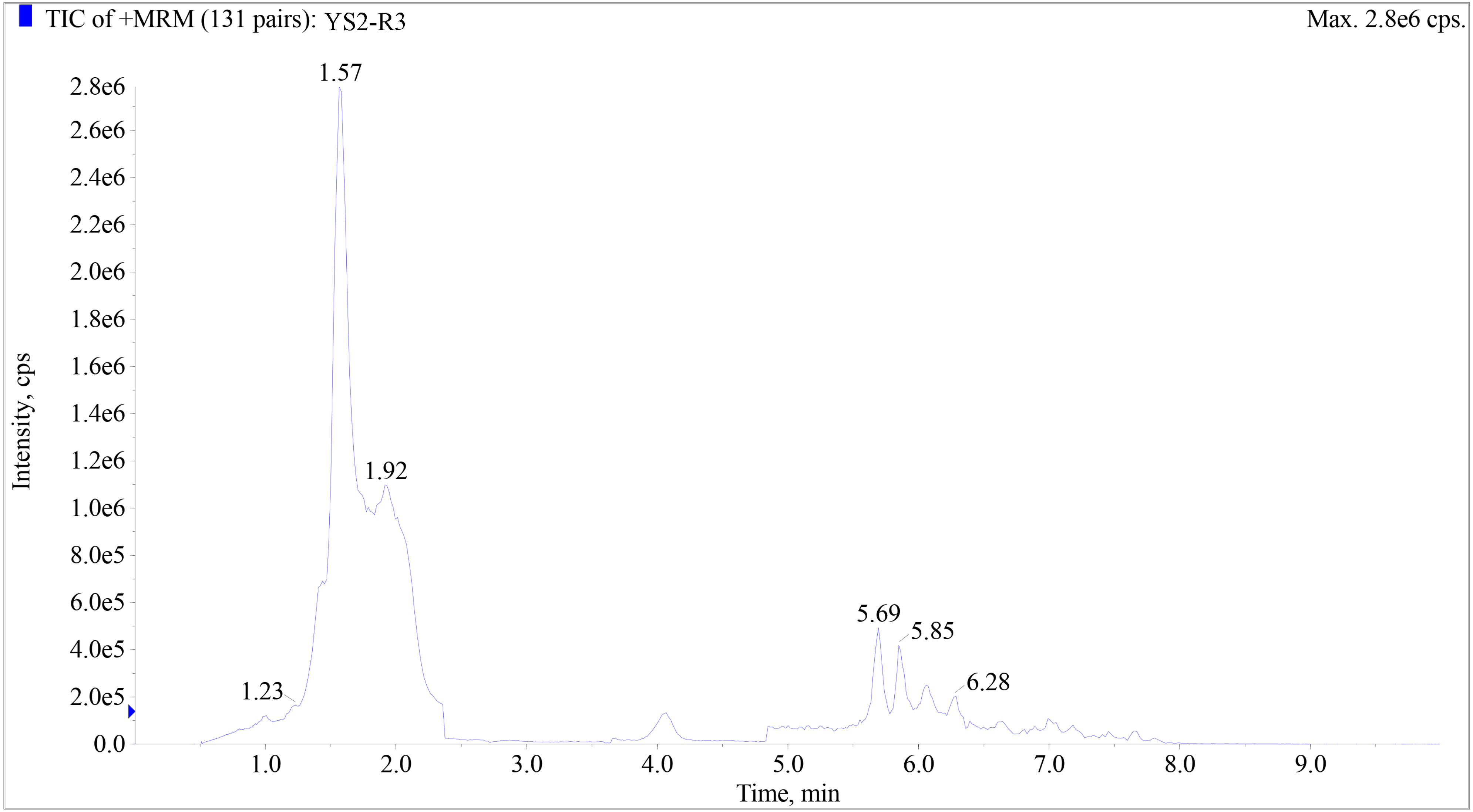

TIC of +MRM (131 pairs): YS2-R3
Max. 2.8e6 cps.
1.57
1.92
1.23
5.69
5.85
6.28
Intensity, cps
Time, min
2.8e6
2.6e6
2.4e6
2.2e6
2.0e6
1.8e6
1.6e6
1.4e6
1.2e6
1.0e6
8.0e5
6.0e5
4.0e5
2.0e5
0.0
1.0
2.0
3.0
4.0
5.0
6.0
7.0
8.0
9.0

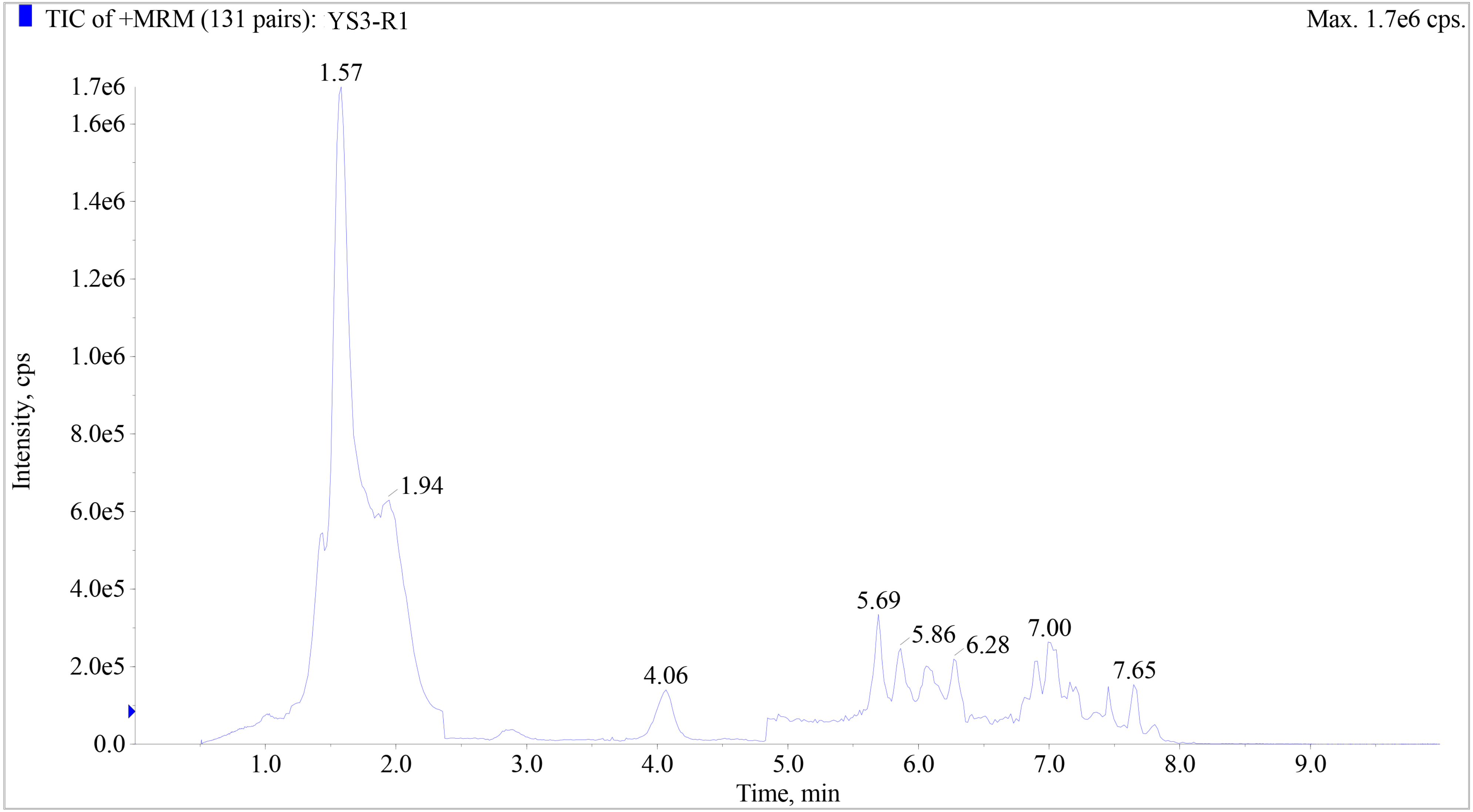

TIC of +MRM (131 pairs): YS3-R1
Max. 1.7e6 cps.
1.57
1.94
4.06
5.69
5.86
6.28
7.00
7.65
Intensity, cps
1.7e6
1.6e6
1.4e6
1.2e6
1.0e6
8.0e5
6.0e5
4.0e5
2.0e5
0.0
1.0
2.0
3.0
4.0
5.0
6.0
7.0
8.0
9.0
Time, min

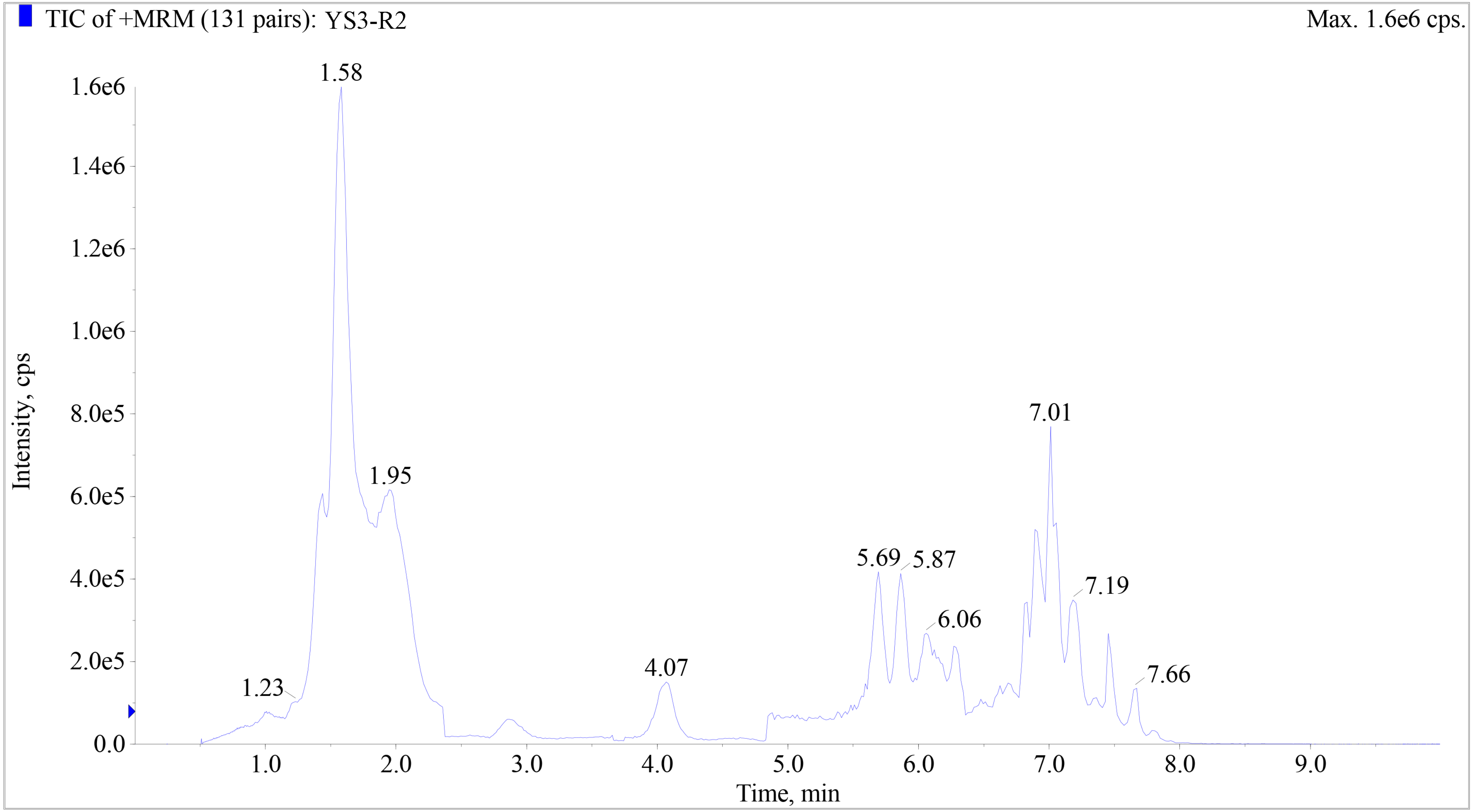
TIC of +MRM (131 pairs): YS3-R2
Max. 1.6e6 cps.
Intensity, cps
Time, min
1.6e6
1.4e6
1.2e6
1.0e6
8.0e5
6.0e5
4.0e5
2.0e5
0.0
1.0
2.0
3.0
4.0
5.0
6.0
7.0
8.0
9.0
1.58
1.95
1.23
4.07
5.69
5.87
6.06
7.01
7.19
7.66

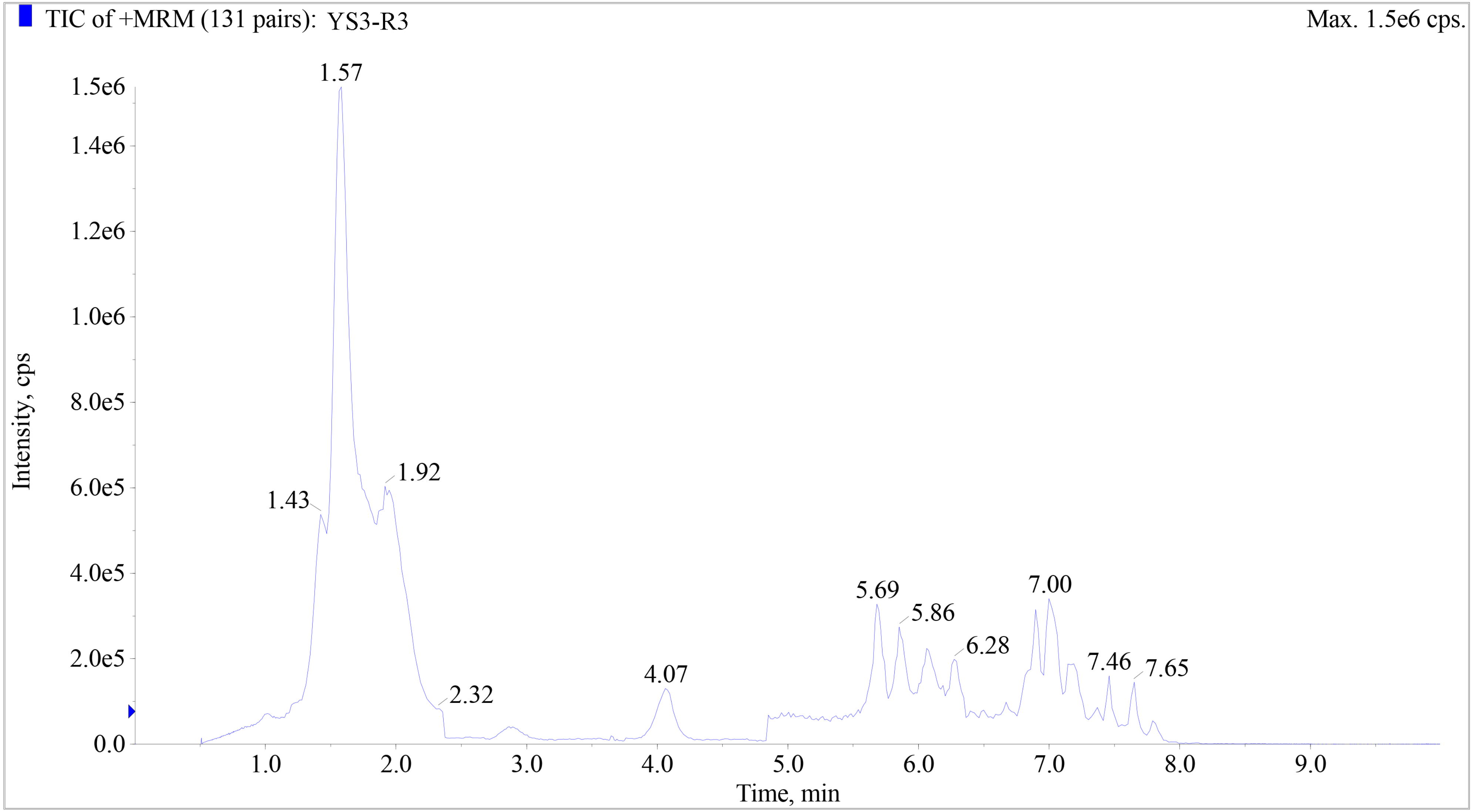

TIC of +MRM (131 pairs): YS3-R3
Max. 1.5e6 cps.
1.57
1.43
1.92
2.32
4.07
5.69
5.86
6.28
7.00
7.46
7.65
1.5e6
1.4e6
1.2e6
1.0e6
8.0e5
6.0e5
4.0e5
2.0e5
0.0
Intensity, cps
1.0
2.0
3.0
4.0
5.0
6.0
7.0
8.0
9.0
Time, min

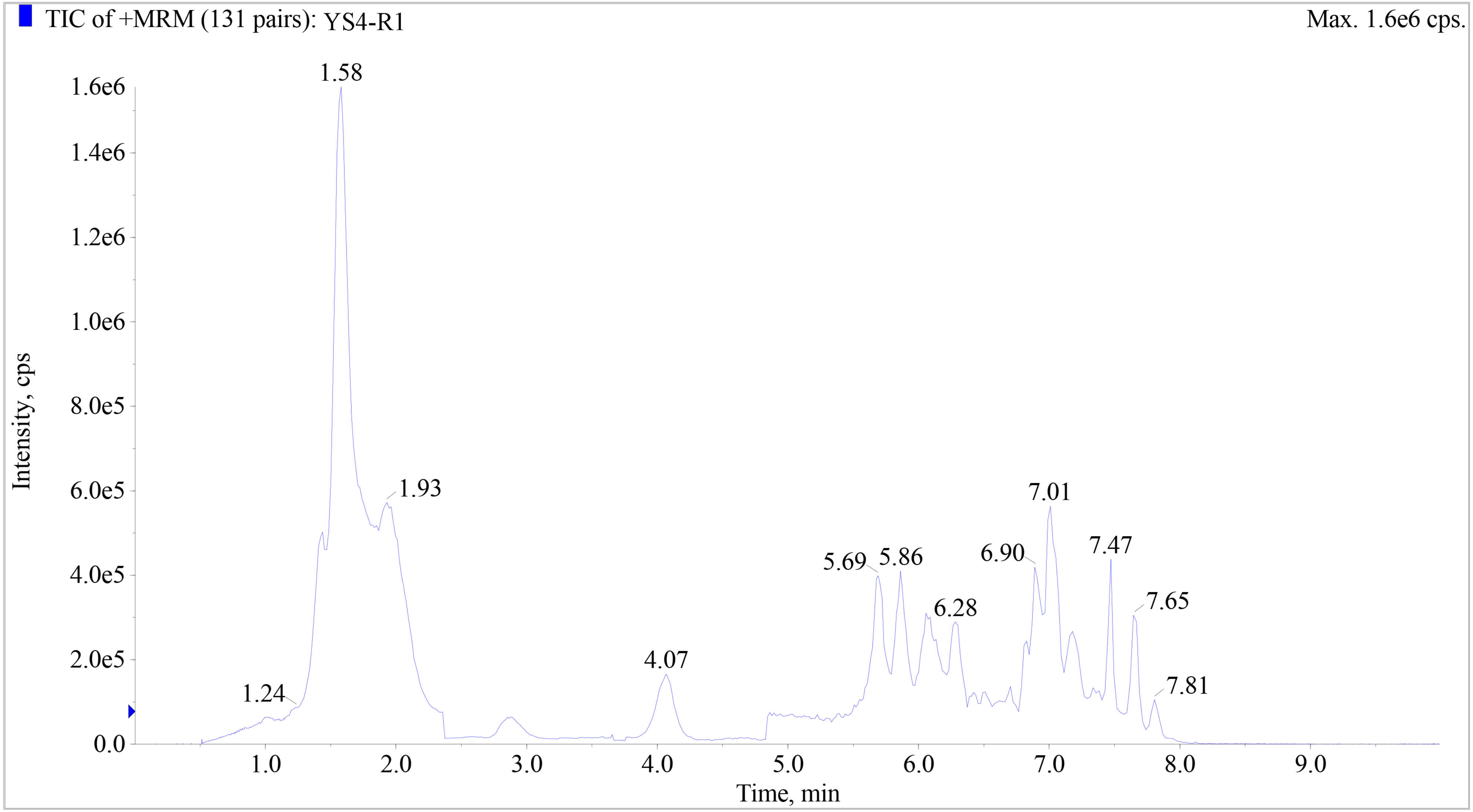

TIC of +MRM (131 pairs): YS4-R1
Max. 1.6e6 cps.
Intensity, cps
1.6e6
1.4e6
1.2e6
1.0e6
8.0e5
6.0e5
4.0e5
2.0e5
0.0
1.58
1.93
1.24
4.07
5.69
5.86
6.28
6.90
7.01
7.47
7.65
7.81
1.0
2.0
3.0
4.0
5.0
6.0
7.0
8.0
9.0
Time, min

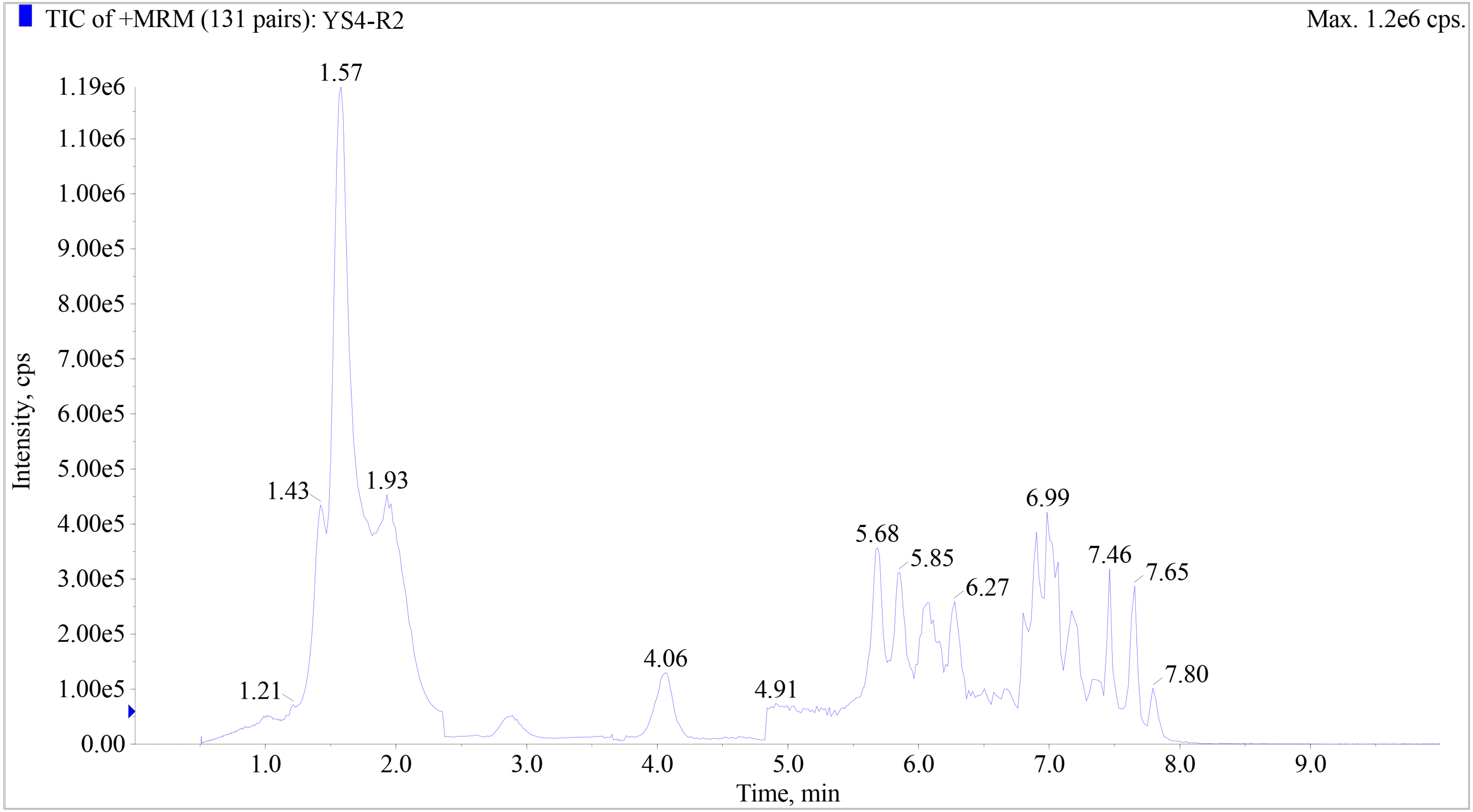
TIC of +MRM (131 pairs): YS4-R2
Max. 1.2e6 cps.
Intensity, cps
1.19e6
1.10e6
1.00e6
9.00e5
8.00e5
7.00e5
6.00e5
5.00e5
4.00e5
3.00e5
2.00e5
1.00e5
0.00
1.57
1.43
1.93
1.21
4.06
4.91
5.68
5.85
6.27
6.99
7.46
7.65
7.80
1.0
2.0
3.0
4.0
5.0
6.0
7.0
8.0
9.0
Time, min

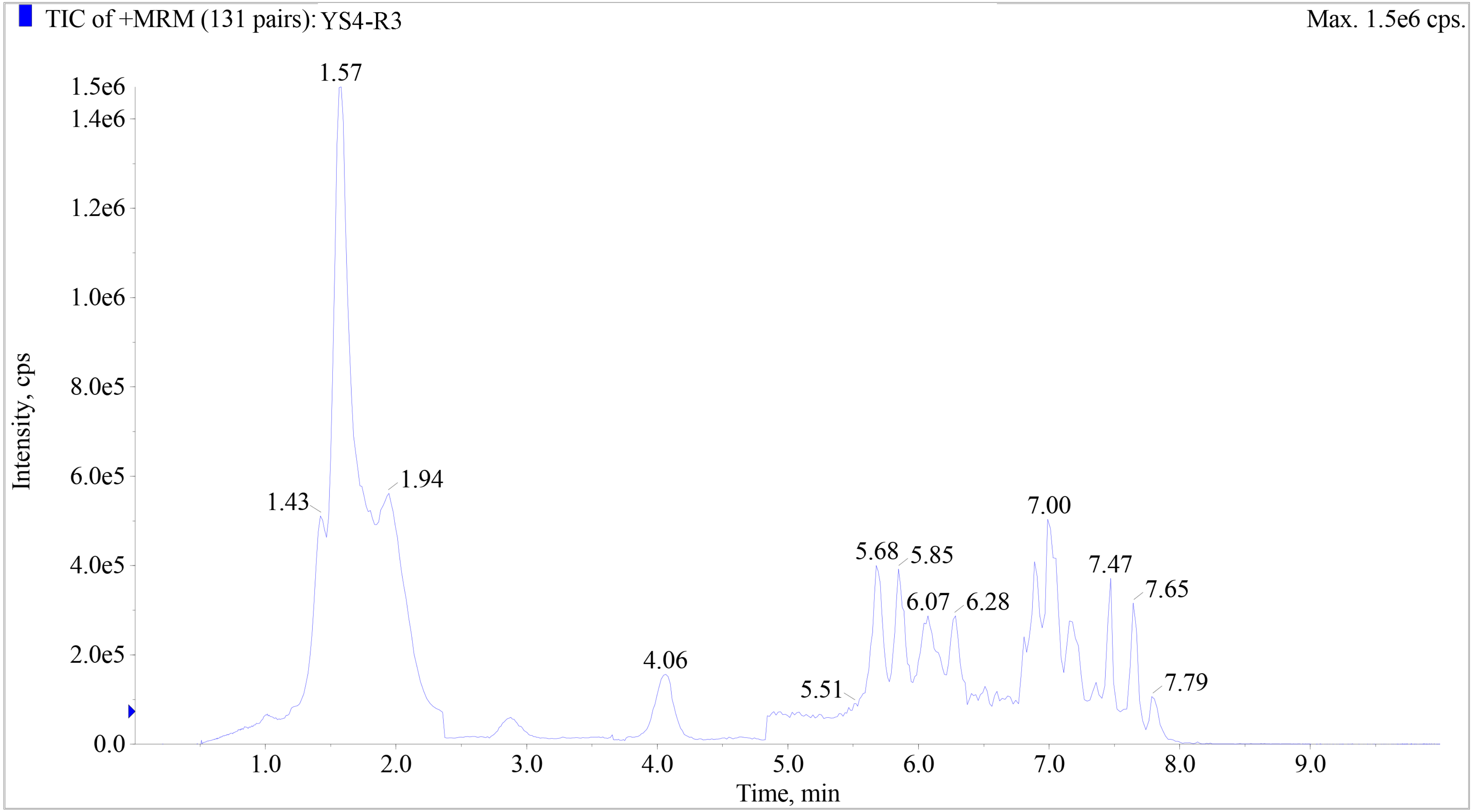

TIC of +MRM (131 pairs): YS4-R3
Max. 1.5e6 cps.
Intensity, cps
Time, min
1.5e6
1.4e6
1.2e6
1.0e6
8.0e5
6.0e5
4.0e5
2.0e5
0.0
1.0
2.0
3.0
4.0
5.0
6.0
7.0
8.0
9.0
1.57
1.43
1.94
4.06
5.51
5.68
5.85
6.07
6.28
7.00
7.47
7.65
7.79

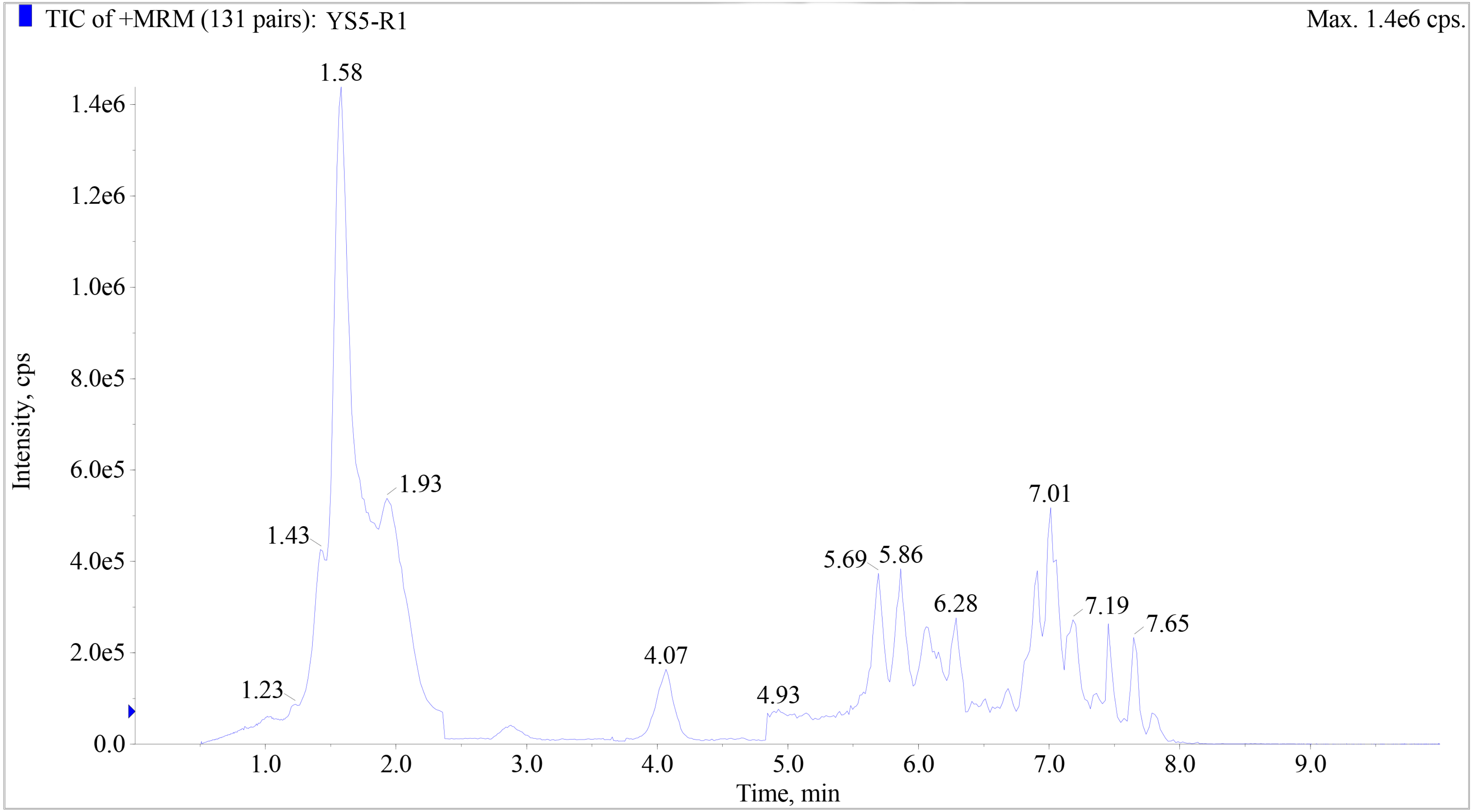

TIC of +MRM (131 pairs): YS5-R1
Max. 1.4e6 cps.
Intensity, cps
1.4e6
1.2e6
1.0e6
8.0e5
6.0e5
4.0e5
2.0e5
0.0
1.58
1.93
1.43
1.23
4.07
4.93
5.69
5.86
6.28
7.01
7.19
7.65
1.0
2.0
3.0
4.0
5.0
6.0
7.0
8.0
9.0
Time, min

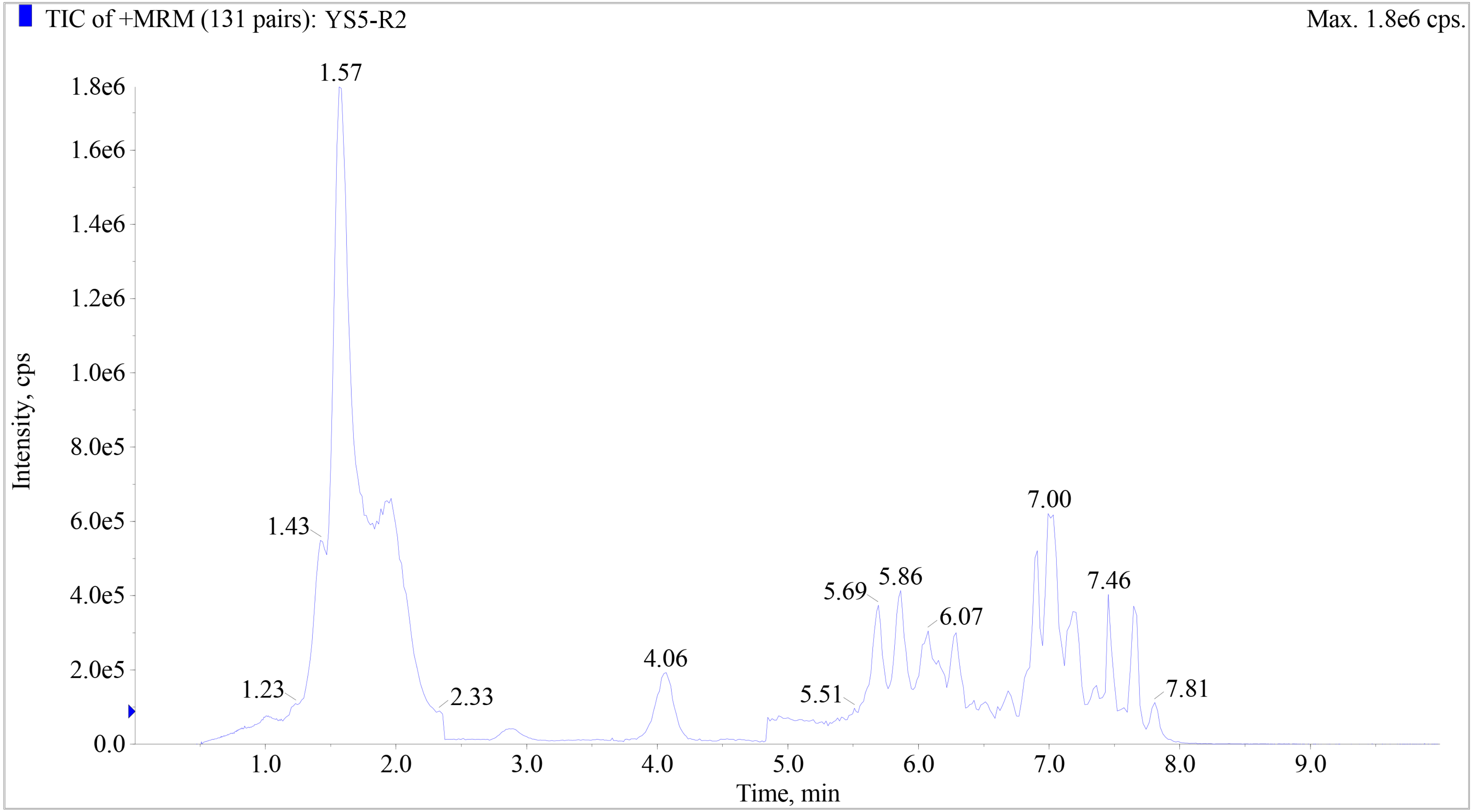

TIC of +MRM (131 pairs): YS5-R2
Max. 1.8e6 cps.
Intensity, cps
1.8e6
1.6e6
1.4e6
1.2e6
1.0e6
8.0e5
6.0e5
4.0e5
2.0e5
0.0
1.0
2.0
3.0
4.0
5.0
6.0
7.0
8.0
9.0
Time, min
1.57
1.43
1.23
2.33
4.06
5.51
5.69
5.86
6.07
7.00
7.46
7.81

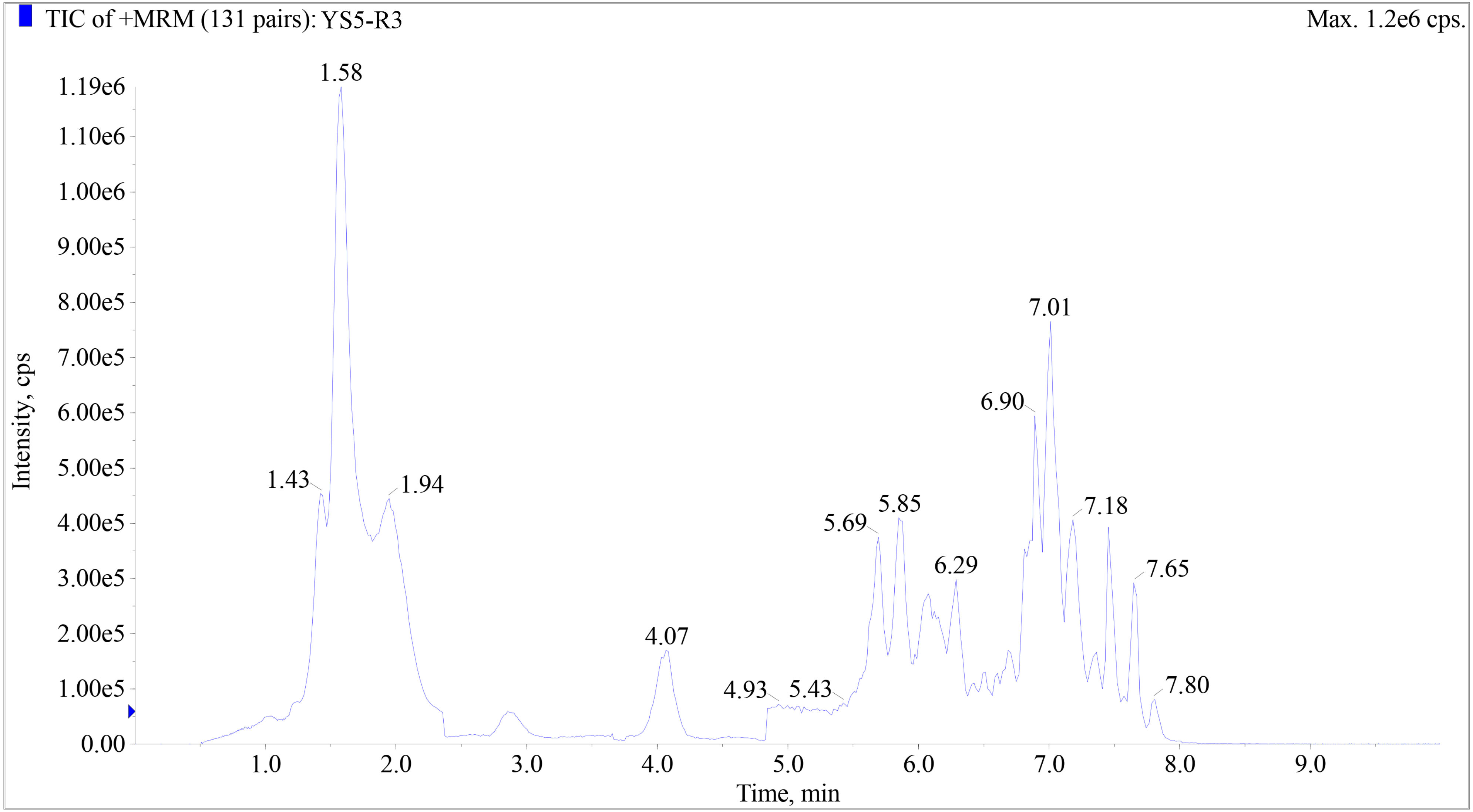
TIC of +MRM (131 pairs): YS5-R3
Max. 1.2e6 cps.
Intensity, cps
1.19e6
1.10e6
1.00e6
9.00e5
8.00e5
7.00e5
6.00e5
5.00e5
4.00e5
3.00e5
2.00e5
1.00e5
0.00
1.58
1.43
1.94
4.07
4.93
5.43
5.69
5.85
6.29
6.90
7.01
7.18
7.65
7.80
1.0
2.0
3.0
4.0
5.0
6.0
7.0
8.0
9.0
Time, min
